# De Novo Structural Elucidation of Acylglycerols by Supercritical Fluid Chromatography and Collision-Induced Dissociation of Electron-Deficient Precursor Ions

**DOI:** 10.1021/acs.analchem.4c05976

**Published:** 2025-02-07

**Authors:** Patrick Mueller, Gérard Hopfgartner

**Affiliations:** Life Sciences Mass Spectrometry, Department of Inorganic and Analytical Chemistry, University of Geneva, 24 Quai Ernest Ansermet, CH-1211 Geneva 4, Switzerland

## Abstract

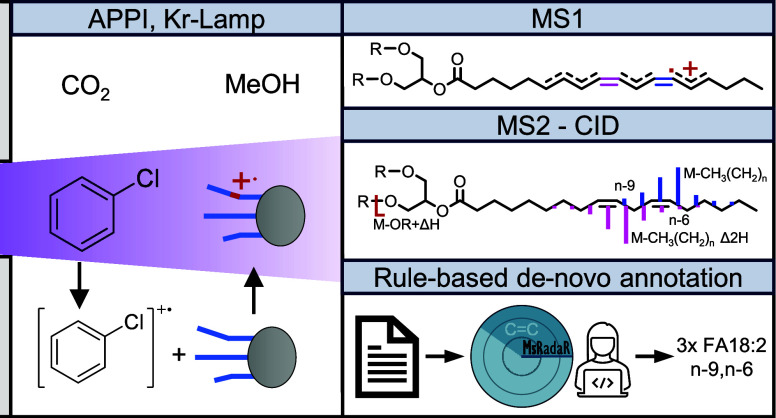

Various mass-spectrometric lipidomics approaches are described to resolve double bond (DB) positions, fatty acid attachments, and *sn*-position but require dedicated instrumentation or chemical derivatization. In this study, we demonstrate that dopant-assisted atmospheric-pressure photoionization (dAPPI) using chlorobenzene as a dopant generates radical cations (M^+.^) and [M-H]^+^ cations of acylglycerols termed Electron Deficient Precursor Ions (EDP). Observed [M-H]^+^ ions are derived from radical hydrogen abstraction from M^+.^ ions. Collision-induced dissociation of EDP ions allows rule-based de novo annotation of DB positions, fatty acid attachments, and *sn*-position. Among 33 acylglycerol standards, selective ionization for acylglycerols with ≥1 DB was observed, where acylglycerols with ≤3 DB formed mainly [M-H]^+^ ions and those with ≥4 M^+.^ ions. EDP-CID generated characteristic intensity ratios of the fragment peaks for *sn*-positions and double-bond indicative elemental formula losses (EFL) originating from cleavages along the fatty acid alkyl chain with intensity maxima adjunct to DB positions. DB annotation was performed by following EFL series, such as M-CH_3_(CH_2_)*_n_* for the first DB. For each DB lost, a Δ2H shift is observed (M-CH_3_(CH_2_)*_n_* Δ2H_DB-1_) and used for the annotation of following DBs. Additional fragmentation series, indicative of DB positions, were observed after loss of hydroxyl groups or fatty acids (M-FA-CH_3_(CH_2_)*_n_*). MsRadaR, an R package, was developed allowing to navigate through EDP-CID spectra and visualize relevant EFL series. Last, supercritical fluid chromatography coupled to dAPPI-EDP-CID was applied for the analysis of 57 acylglycerols in linseed oil with complete DB position characterization of 9 diglycerides and 24 triglycerides.

## Introduction

Acylglycerols are lipid molecules, composed of a glycerol moiety, esterified with one (monoglycerides) to three fatty acids (triglycerides).^1^ Triglycerides play a crucial role as the main storage form of fatty acids for energy homeostasis and lipid transport in Eukarya, where on-demand enzymatic hydrolysis can release fatty acids as well as di- and monoglycerides. This does entail triglycerides in the metabolism of mono and diglycerides, which are involved in various cellular signaling pathways and act as precursors for other lipid molecules, such as phospholipids and bioactive fatty acids.^2,3^ Certainly, the vast involvement in metabolic processes allows to utilize acylglycerols as biomarkers for abnormal phenotypes caused by disease, medication, and drug abuse, including metabolic storage disorders, radiation-induced fibrosis, and tetrahydrocannabinol consumption.^3,4^ Despite that, differences in the triglyceride compositions of vegetable oils can be used to detect food fraud, whereas acylglycerols and their degradation products are used to probe the composition and age of artworks, archeological objects, and fingerprints.^5^ Last, acylglycerols and other glycerolipids are increasingly used for pharmaceutical products as active substances and excipients, such as lipid nanoparticles for mRNA vaccines.^6^

Over the years, mass spectrometry (MS) has established as a standard tool for glycerolipid analysis, where soft ionization techniques, such as electrospray ionization (ESI), atmospheric-pressure chemical ionization (APCI) or atmospheric-pressure photoionization (APPI) with high-resolution mass spectrometry (HRMS) and collision-induced dissociation of even electron precursors (EE-CID) enable identification glycerolipid species and their fatty acid composition.^7,8^ In the case of ammonium adducts of triglycerides, CID fragmentation allows assigning stereospecific numbering(*sn*) positions of fatty acids at the glycerol backbone based on intensity ratios of fatty acid losses, with a preference for *sn*-1/3 losses.

In order to understand the biological significance of single glycerolipids in complex systems, more reliable and advanced MS techniques are necessary to distinguish between structural isomers, which differ in *sn*-positions, carbon branching sites within fatty acids as well as DB localizations and their corresponding stereo configuration.^9^ Various de novo structural analysis approaches were reported to spot DB positions, including ozone-induced dissociation (OzID),^10^ post-column derivatization techniques such as the coupling of the Paternò–Büchi reaction to mass spectrometry (PB-MS),^11^ electron-activated dissociation (EAD),^12,13^ and ultraviolet-photodissociation (UVPD).^14^ These techniques do generate DB-specific fragments for various classes of lipids but do not allow to retrieve *sn*-positions within single MS2 experiments, except EAD.^9^ More specifically, EAD of sodium adducts of triglycerides generates unique fragments for fatty acids at *sn-*2 and *sn-*1/*sn-*3 positions.^12^ However, MS3 experiments allow to assign *sn*-positions and *sn*-specfic DB positions of fatty acids by methods such as PB-MS3 or UVPD-MS3 of dioxolane (diacylglycerol) fragments generated by CID of sodium adducts of lipids.^15^

These techniques provide a gain in structural information but require elevated reaction times, which challenges the compatibility with fast LC analyses. OzID^16^ requires reaction times from 10 to 200 ms range (OzID at an intermediate pressure, without precursor selection) to seconds (OzID in vacuum, with precursor selection). The experiment cycle time for EAD and UVPD depends on the MS instrumentation. In the case of EAD, reaction times (typically 150 ms) and accumulation times (approximately 1s) might be required for adequate reaction yields and signal-to-noise ratios of low-abundant lipid species.^12^ However, by compromising MS2 spectra quality, shorter accumulation times can be used, which increases the number of precursors that can be monitored in a single LC-run.^17^ This is critical for the analysis of complex biological samples, where chromatographic separation of isobaric and isomeric species is mandatory for unambiguous lipid identification. Analyses by gas chromatography with electron ionization (GC-EI-MS) of mono- and diglycerides as derivatives have been reported with improved DB position-specific fragmentation along with *sn-*positions-related fragmentation but are limited to the molecular weight of the lipid.^18^ For glycerolipids, two main LC separation mechanisms are applied, either the compounds are separated by the carbon-chain length and the number and position of double bonds (lipophilicity) using reversed-phase liquid chromatography or by their headgroup (hydrophilicity) using hydrophobic-liquid-interaction-chromatography.^7^ Supercritical fluid chromatography (SFC) has also been demonstrated to be a powerful separation technique of nonpolar lipids, such as triglycerides.^19^ Only a few studies investigated the utilization of APPI for lipidomic studies, but no radical cations were observed independent from ion-source, lamp type, and MS instrument used.^8,20,21^ Instead, protonated molecules, ammonium adducts, and in-source fragments were observed. Ion speciation in dopant-assisted APPI depends on analyte properties and ionization conditions, such as dopant molecule, mobile phase choices as well as their corresponding flow rates.^22^ Certainly, solvent-dopant combinations with typical LC solvents play the most prominent rule and can prevent ionization, allow radical cation formation, or lead to predominant analyte protonation. Methanol as the mobile phase in combination with halogenated benzenes, such as chlorobenzene, has proven as superior combination for radical cation formation.^23,24^ We recently demonstrated that under well-controlled μLC conditions with post-column addition of methanol, radical cations could be generated by chlorobenzene-assisted atmospheric-pressure photoionization (APPI) of diverse compound classes including steroids, isoprenoids, and polyketides. In addition, CID fragmentation of radical cations generate EI like fragmentation spectra which can be utilized for EI library searches.^24^ Similar to μLC with post-column addition, SFC with methanol as the polar mobile phase additive is a suitable separation technique that allows to maintain radical cations generated by chlorobenzene-assisted APPI.^25^

In the present work, we investigate the application of APPI-MS with chlorobenzene as a dopant for the formation of EDP ions (M^+.^ and [M-H]^+^) of acylglycerol and subsequent fragmentation by CID. Based on the rational CID fragmentation of EDP, an R package for rule-based de novo annotation of DB positions was developed. Finally, the applicability of APPI-CID is demonstrated for the analysis of acylglycerols by SFC-MS in linseed oil using data-dependent acquisition (DDA).

## Experimental Section

### Chemicals

Standard compounds were obtained from Cayman, Larodan, Sigma-Aldrich, and Supelco (Table S1). Stock solutions were prepared in methanol, ethanol, toluene, chloroform, or chlorobenzene. Methanol, ethanol, and chloroform were from Fisher Chemical, chlorobenzene was from Sigma-Aldrich, toluene was from Carl Roth, ammonium formate was from Honeywell Fluka, and CO_2_ 4.5 was from PanGas. Linseed oil was from a Swiss supermarket (Alnatura Leinöl nativ, Migros, Geneva, Switzerland) aliquoted and stored at −20 °C until use.

### Supercritical Fluid Chromatography

SFC was performed by using a Nexera UC system (Shimadzu, Kyoto, Japan). Acylglycerol standards and linseed oil lipids were separated on a Viridis HSS C18 SB Column (Waters, 100 Å, 1.8 μm, 3 mm × 100 mm) at 40 °C using a gradient from 5% MeOH to 25% MeOH in 5.25 min and to 100% in 6 min with a washing step until 9 min for standards and adjusted for linseed oil with 10 to 25% MeOH in 5.25 min. The back pressure regulator was set to 10 MPa and 50 °C. The total flow rate was set to 1 mL/min with a makeup flow rate of 200 μL/min for APPI and 50 μL/min for ESI. Ammonium formate 0.1% (w/v) was added to MeOH for ESI analysis.

### Mass Spectrometry

All samples were analyzed on a QqTOF (6600 TripleTOF, SCIEX, Concord, ON, Canada, resolution = ) equipped with an APPI source (PhotoSpray, SCIEX, Concord, ON, Canada) using chlorobenzene as dopant (20 μL/min using a Hamilton syringe) or an ESI DuoSpray ion-source (SCIEX, Concord, ON, Canada). The ion-source settings for APPI and ESI are given in Table S2. All of the TOF-MS experiments were performed with an accumulation time of 100 ms. CID spectra of acylglycerol standards were acquired from 10 to 70 eV in steps of 5 eV with accumulation times of 50 ms. For Linseed oil analysis, data-dependent acquisition of the 10 highest ions was performed using a collision energy spread from 10 to 70 eV with accumulation times of 85 ms. Detailed information about mass range and isolation width are available in Table S3.

### Linseed Oil Analysis

Features in MS1 were detected using MarkerView (SCIEX, Concord, ON, Canada), elemental formulas, and lipid species assigned based on exact mass and validated by MS/MS fragmentation. Retention times and intensities were retrieved using MasterView (SCIEX, Concord, ON, Canada) and were averaged across all 3 replicates.

### Data Analysis Using MsRadaR

MS2 spectra were exported as centroided text files from PeakView (SCIEX, Concord, ON, Canada) and subsequently processed and analyzed using the MsRadaR R package.^26^

## Results

### Ionization of Acylglycerols by Chlorobenzene-Assisted APPI

**Figure 1:**
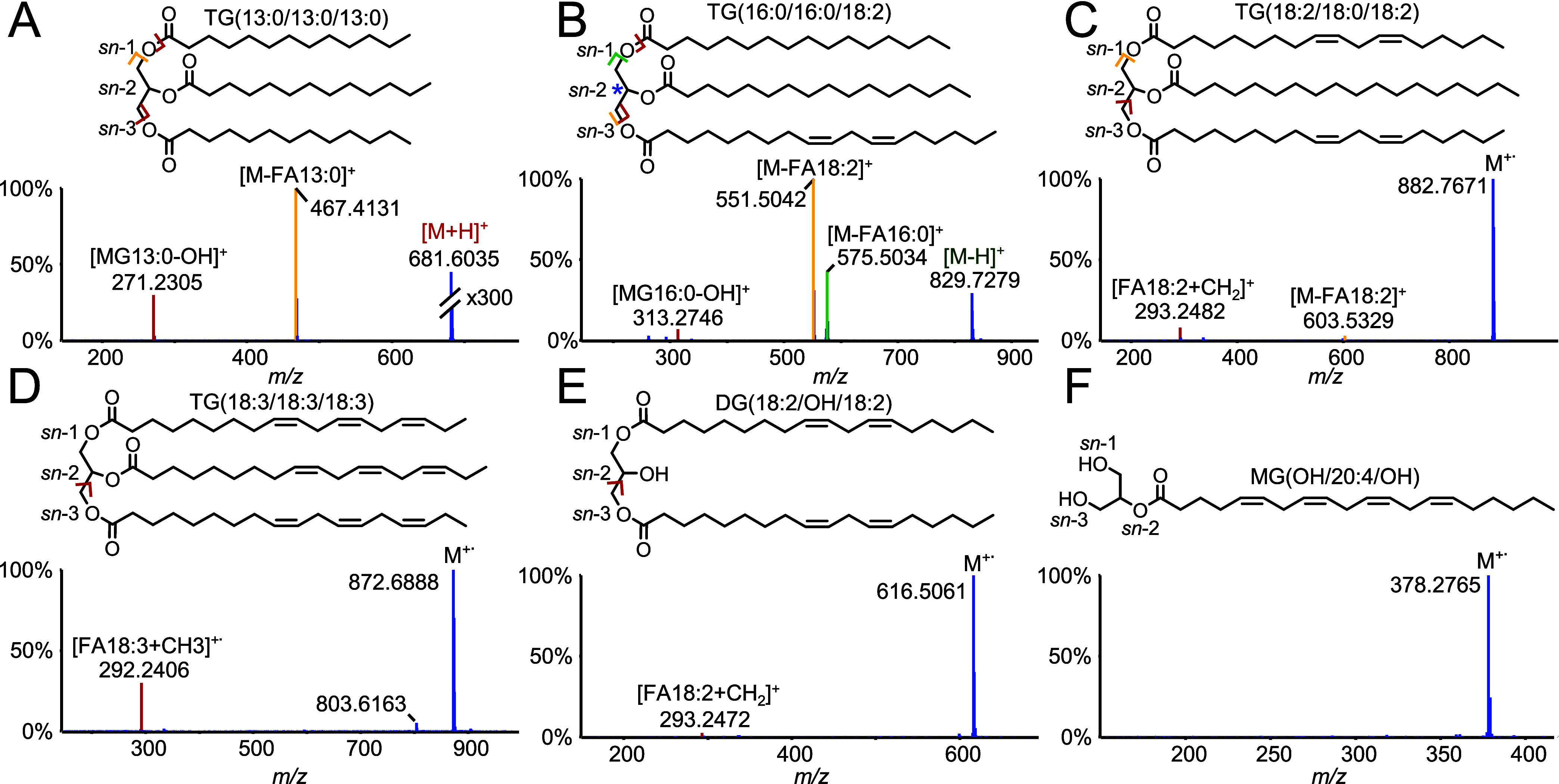
SFC-APPI-MS1 spectra of 6 acylglycerol standards with (A) TdTdTd, (B) PPL, (C) LSL, (D) LnLnLn, (E) 1,3-LL, and (F) 2-Ar. Td= tridecylic acid, *P* = palmitic acid, L = linoleic acid, S = steric acid, Ln = α-linolenic acid, Ar = arachidonylglycerol.

**Figure 2:**
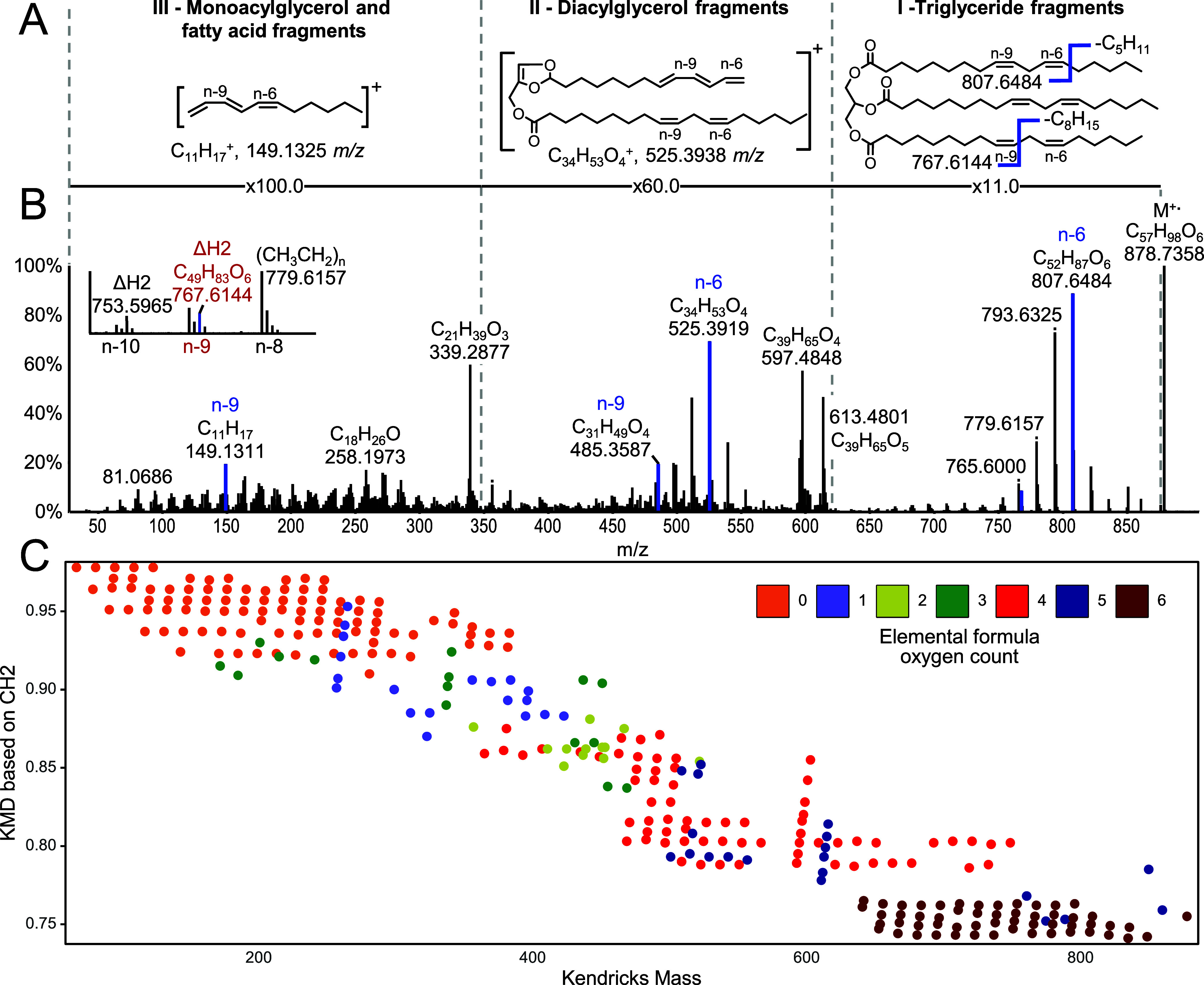
EDP-CID of the LLL radical cation with (A) exemplary diagnostic fragments and (B) 35 eV EDP-CID spectra of the LLL radical cation. The inset of (B) shows a zoom onto the DB-specific EFL series of position n-9. Blue lines indicate diagnostic fragment ions for DB positions and (C) Kendrick plot with elemental formula assignment of each ion from the EDP-CID spectrum, which does have at least 2 counts and a relative intensity of 0.05%. Colors represent the number of oxygen within the elemental formula of each ion.

SFC-APPI-MS with chlorobenzene as a dopant undergoes structural informative ionization and ion speciation of acylglycerols based on the number of DBs. Acylglycerols without DBs predominantly dissociate into diacylglycerol, monoacylglycerol, and fatty acid fragment ions with almost no protonated analyte ion present (Figures 1A and S1–S3). The observed in-source fragmentation pattern is similar to that observed in previous studies from others.^21^ With an increasing number of DBs and distribution across all fatty acids, the intensity of intact ED analyte ions increases (Figures 1B–F and S1–S8). Acylglycerols with ≤3 DBs form mainly [M-H]^+^ ions by hydrogen radical abstraction as well as in-source fragments (Figures 2B, S1, S4–S6). With an increase in the number of DBs, the contribution of M^+.^ increases and does dominate for acylglycerols with ≥4 DBs (Figure 1C–F, Figures S1, S7, S8). In the case of 2-arachidoniyl glycerol, a dominant radical cation is formed when using APPI (Figure 1F), while for (+)ESI, the analyte signals are distributed across 6 different ions, including the protonated analyte, sodium, and ammonium adducts as well as in-source fragments (Figure S7E).

### EDP-CID Fragmentation and Annotation of Unsaturated Acylglycerols

**Figure 3:**
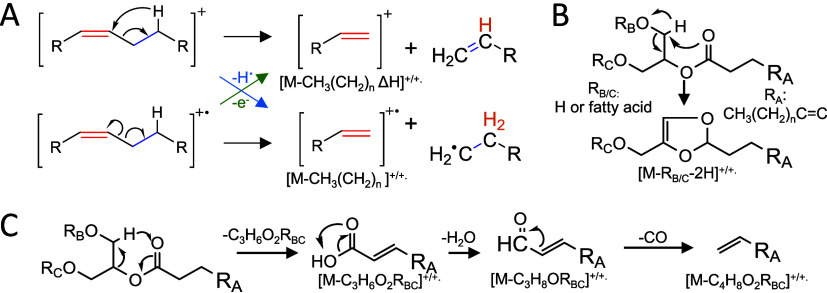
Putative EDP-CID fragmentation scheme of M^+.^ and [M-H]^+^ ions with (A) neutral and radical loss leading to diagnostic DB fragments, (B) Acylglycerol cyclization acting as intermediate starting point for additional DB series, and (C) Generation of fatty acid like fragments, which can act as starting point for additional DB fragments.

CID fragmentation of EE acylglycerols precursors formed by ESI generally yields abundant fragment ions which allow to retrieve the fatty acid composition but fail to identify DB positions.^27^ Unsaturated acylglycerol (M^+.^ and [M-H]^+^) ions formed by APPI, on the other hand, yield information-rich fragmentation spectra, with multiple diagnostic fragment ions related to the fatty acid composition and corresponding DB positions (Figure 2). For the EDP-CID spectrum of the trilinoleoyl glycerol (LLL) radical cation, the most prominent fragment ions represent alkyl cleavages along the fatty acid chain (ΔCH2, Δ14.016) originating from radical losses, which can be represented as M-CH_3_(CH_2_)*_n_*. DB positions can be assigned by spotting the intensity maxima of these alkyl cleavages, reflecting bond cleavages in front of a DB position. For the first DB positions, this represents a radical loss of C_5_H_11_ or M-CH_3_(CH_2_)_4_ leading to a *m*/*z* of 807.6484 (Figure 2AB). For the second DB positions, the loss of the first DB (CH=CH) induces a Δ*2H* shift, which indicates a neutral loss of C_8_H_15_ or M-CH_3_(CH_2_)_7_ Δ*2H* leading to a *m*/*z* of 767.6144 (Figure 2B inset). Important to note is that elemental formula loss (EFL) series do not stop after the occurrence of a DB and are hypothesized to originate from charge and DB migrations within the ion-source similar to observations made in EI.^28^ Additional diagnostic ions with similar fragmentation series are observed from diacylglycerol (M-FA-3H-CH_3_(CH_2_)*_n_*) or fatty acid fragments, which allow cross-validation of the DB positions (Figure 2AB). In addition, the fatty acid composition can be evaluated using diagnostic diacylglycerol fragment ions (613.4801 and 597.4848 *m*/*z*), monoacylglycerol fragment ions (339.2877 *m*/*z*), and fatty acid fragment ions (258.1973 *m*/*z*) (Figure 2B). This allows to perform de novo structural elucidation of fatty acid composition and DB positions of TG(18:2/18:2/18:2) with three times n-6 for the first and n-9 for the second DB. For interpretation, it is important to identify the start of alkyl chain cleave series, such as the precursor ion, fatty acid losses, or glycerol losses. This is facilitated by assigning the elemental composition of each single fragment ion and highlighting fragment ions by the number of oxygen atoms. As illustrated in the Kendrick plot of LLL, a high variety of EFL series are formed, which differ only by the number of hydrogen atoms (Figure 2C). These can be explained by the presence of DB positions as well as the occurrence of both radical and neutral losses, leading to even or odd hydrogen losses (Figure 3A). These findings allow for predicting diagnostic fragment ions, which do have intensity maxima depending on specific DB positions (Tables 1 and S4).

**Table 1:** List of Representative Diagnostic EFLs and Corresponding *m*/*z* Values

double bond information		1st double bond		2nd double bond		3rd double bond	
DB position	carbon losses	radical loss	neutral loss (Δ*H*)	radical loss (Δ2H)	neutral loss (Δ3H)	radical loss (Δ4H)	neutral loss (Δ5H)
n-3	2	-C_2_H_5_	-C_2_H_4_				
		29.0386	28.0313				
n-6	5	-C_5_H_11_	-C_5_H_10_	-C_5_H_9_	-C_5_H_8_	-C_5_H_7_	-C_5_H_6_
		71.0855	70.0783	69.0699	68.0626	67.0542	66.0470
n-9	8	-C_8_H_17_	-C_8_H_16_	-C_8_H_15_	-C_8_H_14_	-C_8_H_13_	-C_8_H_12_
		113.1325	112.1252	111.1168	110.1096	109.1012	108.0939
n-12	11	-C_11_H_21_	-C_11_H_20_	-C_11_H_19_	-C_11_H_18_	-C_11_H_17_	-C_11_H_16_
		153.1638	152.1565	151.1481	150.1409	149.1325	148.1252

Depending on the number of hydroxyl groups (acylglycerol subclass), fatty acid composition, collision energy, and precursor ion species competitive fragmentation reactions can favor preceding losses of functional groups, such as hydroxyl groups, glycerol headgroup or fatty acids (Figure 3BC). Acylglycerols with more than one unsaturated fatty acid typically do have prominent M-CH_3_(CH_2_)*_n_* Δ2*H*_DB-1_ series, which can be used for DB annotation but are less pronounced for acylglycerols with only one unsaturated fatty acid and might be insufficient to annotate all DB positions. For instance, a triglyceride with two FA18:1 does generate diagnostic ions for DBs from the M-CH_3_(CH_2_)*_n_* Δ2*H*_DB-1_ series (Figures S9–S10), while a triglyceride with one FA18:2 does not (Figure S11). Instead, diagnostic ions originating from the diacylglycerol (M-FA-CH_3_(CH_2_)*_n_*) or fatty acid series can be used for annotation (Figure S12). This implies that saturated fatty acids and hydroxyl groups are good leaving groups and that some isobaric acylglycerols (e.g., SLS and OSO) produce different fragmentation spectra due to competitive fragmentation reactions with structurally related kinetics. This effect is more pronounced for monoacylglycerols, which also require lower (20 eV) collision energies than diacyl and triacylglycerols (35 eV) for good intensities of DB-related fragment ions (Figures S9–S78).

### Double Bond Position Assignment Using MsRadaR

**Figure 4:**
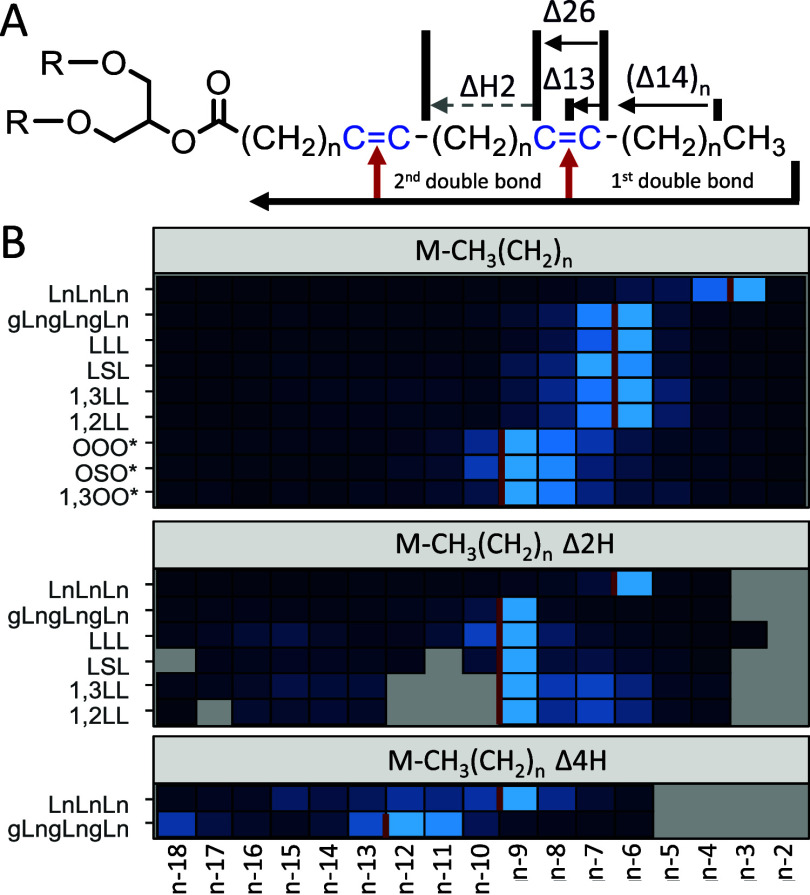
Annotation of DB positions using the MsRadaR R package with (A) an acylglycerol scheme explaining the double bond annotation and (B) a graphical summary of MsRadaR results for a set of 9 acylglycerols. Colorscale of the heatmap represents the relative intensity per DB series and lipid with 0% (dark blue) to 100% (light blue). Gray cells represent missing values and red lines DB positions. L: linoleic acid, S: stearic acid, Ln: α-linolenic acid, gLn: γ-linolenic acid, O: oleic acid.

To facilitate time-consuming manual annotation of fragment-rich spectra generated by EDP-CID, we developed MsRadaR an R package with a de novo structural elucidation pipeline for acylglycerols.^26^ MsRadaR processes centroided mass spectra and extracts relevant fragment ion series to annotate putative double bond positions based on intensity maxima peak picking algorithms. Relevant fragment ion series and putative double bond positions are visualized to facilitate interpretation and validation by the user. In more detail, MsRadaR processes centroided mass spectra and annotates EFLs from defined *m*/*z* values such as the precursor mass. First, *m*/*z* losses are calculated by subtracting all fragment ions from the user-defined *m*/*z* values. Second, EFLs are annotated based on user-defined constrains such as mass error (20 mmu), double bond equivalent (≥−0.5), and maximal elemental composition (elemental formula of the longest fatty acid), where only elemental formulas with the lowest error are retained. Third, the hydrogen count of each experimentally determined EFL is compared to the hydrogen count of a saturated alkyl chain M-CH_3_(CH_2_)*_n_* and used to calculate the hydrogen differences (M-CH_3_(CH_2_)*_n_* Δ*xH*) required for double bond annotation (Figure 4A). Last, relevant M-CH_3_(CH_2_)*_n_* Δ*xH* series are extracted, intensities normalized, slopes calculated, and intensity maxima labeled by intensity maxima peak picking algorithms. Ultimately, this allows performing de novo annotation of double bonds for a wide variety of tri- and diglycerols independent from the selected EDP ion (M^+.^ and [M-H]^+^) (Figure 4B). False annotation can be minimized by including additional measures for DB annotation, considering the intensity slope (Figures S9–S78) and/or additional double-bond specific fragmentation series such as M-FA-CH_3_(CH_2_)*_n_* Δ*xH* (Figures S44, S45, S49, S52, and S56). The intensity slope should continuously increase until a double bond is reached and is, in most cases, maximal at the DB position. After the maximum, a sign change should occur with a minima, 1–2 carbons after the DB position. As in the case of 1,3-dilinoleoyl-2-stearoyl glycerol (LSL), the intensity slope shows that the first DB is at position n-6 and minimal at position n-9, alike the fatty acid fragmentation series (Figures S40–S41).

### EDP-CID Fragmentation and Annotation of Acylglycerol with Mixed Fatty Acid Chains

**Figure 5:**
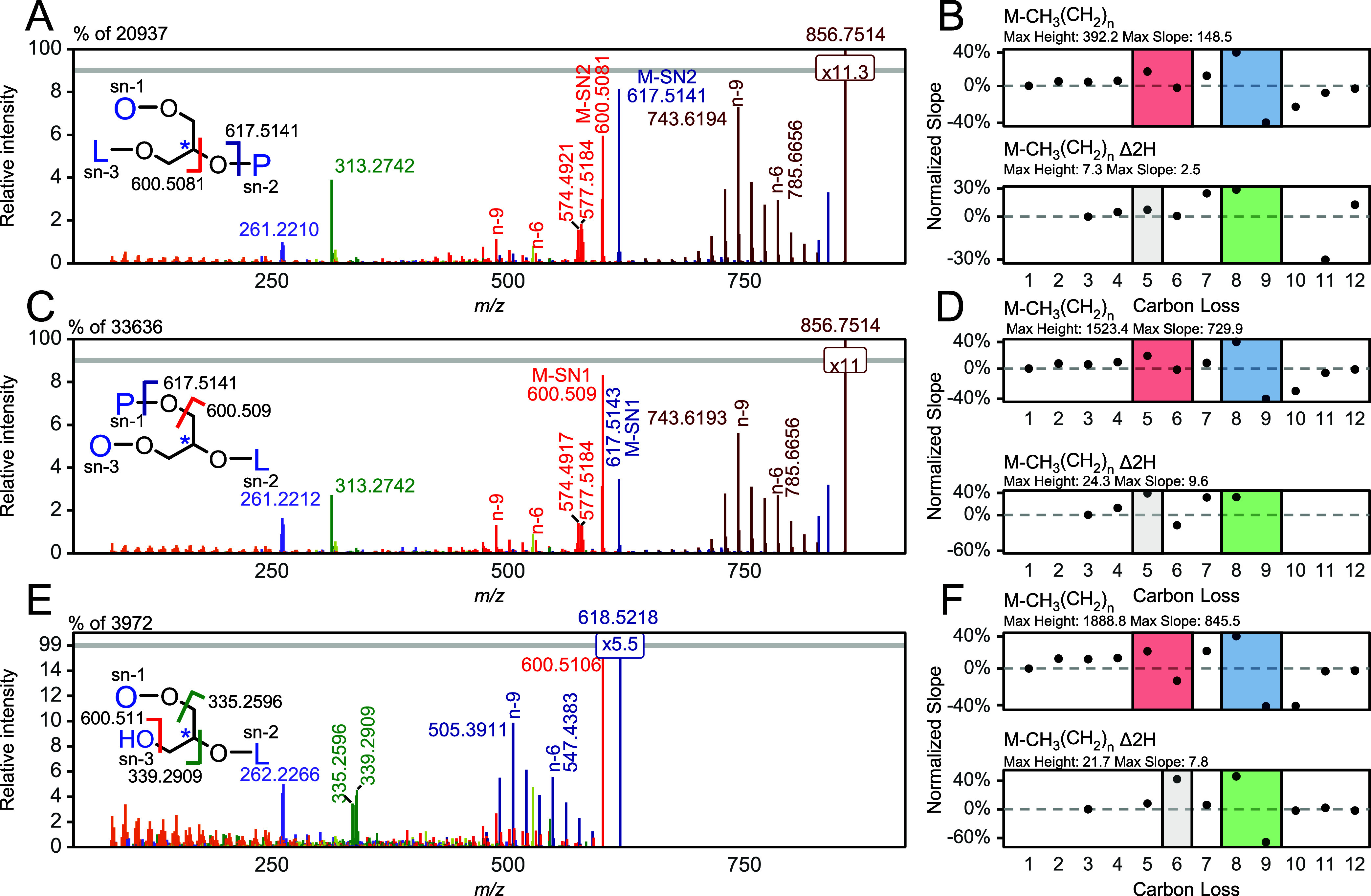
Annotated 35 eV CID spectra using MsRadaR of (A) OPL with (B) extracted EFL series, (C) PLO with (D) extracted EFL series, and (E) 1,3OL with (F) extracted EFL series. Red boxes indicate DB position n-6 of FA18:2, blue DB position n-9 of FA18:1, green DB position n-9 of FA 18:2, and gray the isotopic contribution from M-CH_3_(CH_2_)*_n_* Δ*H* of the CID fragmentation of [M-H]^+^. For mass spectra, gray lines indicate breakpoint of the *y*-axis. Normalized slopes are calculated by dividing the slope by the maximal height of a given series. MG = Monoacylglycerol, DG = Diacylglycerol, TG = Triacylglycerol.

Acylglycerols with 2 or more unsaturated fatty acids with varying DB positions generate overlapping fragmentation series. In that case, DBs can be assigned based on the occurrence of one maxima per unique DB position. For the regioisomeric triglycerides 1-oleoyl-2-palmitoyl-3-linoleoyl-glycerol (OPL) and 1-palmitoyl-2-linoleoy-3-oleoyl-glycerol (PLO) and diglyceride 1-oleoyl-2-linoleoyl-glycerol (OL), the first DB of FA18:2 generates a small maxima indicating position n-6 followed by a second maxima from fatty acid FA18:1 at carbon position n-9 (Figure 5A–C). The use of intensity slopes allows mitigation of the masking of overlapping fragmentation series (Figure 5D–F). For all three acylglycerols, the intensity slope shows two maxima after 5 and 8 carbon losses, followed by two sign changes alongside 2 minima after 6 and 9 carbon losses. This indicates that the first DB positions as n-6 for FA18:2 and n-9 for FA18:1. The second DB with carbon position n-9 of FA18:2 generates a slope maxima after 8 carbon losses, followed by either missing signals for the triglycerides or a sign change with a minima after 9 carbon losses for the diglyceride. The slope maxima show normalized slopes of over 15% and thus, can be accounted as significant. In addition to the intensity slope maxima related to the DB position n-9, another intensity maxima after 5 carbon losses for the triglycerides and 6 carbon losses for the diglyceride OL are observed. These are most likely caused by the isotopic contribution of the M-CH_3_(CH_2_)*_n_* Δ*H* series from almost equally intense [M-H]^+^ ions of the same analyte (Figure S6D–G). This does not interfere with the interpretation of the spectra, as the second DB positions indicated by the M-CH_3_(CH_2_)*_n_* Δ2*H* series cannot occur before the first DB positions indicated by the M-CH_3_(CH_2_)*_n_* series. Moreover, after the loss of FA18:0 from a triglyceride and OH loss in the case of a diglyceride, a second DB fragmentation series appears with a fragmentation pattern very similar to that of the M-CH_3_(CH_2_)*_n_* Δx*H* loss series from the triglyceride or diglyceride precursors that can be used for DB position validation. Despite DB-related fragment ions, pronounced diacylglycerol, monoacylglycerol, and fatty acid fragments can be observed. It is to expect that the isotopic contribution of [M-H]^+^ ions is the major origin of these fragment ions, which leads to lower abundant fatty acid fragment ions for highly unsaturated acylglycerols.(Figures S9–S78) In general, intensity ratio changes are observed for regioisomeric acylglycerols. If a saturated fatty acid is situated on *sn*-position 2, a dominant cleavage between the α carbon of the fatty acid and the oxygen of the glycerol backbone is observed (*m*/*z* 617.5141). If situated at the *sn*-position 1/3, the fatty acid loss predominantly leads to a cleavage between the glycerol moiety and the carboxy group of the fatty acid (*m*/*z* 600.509). This suggests that the ratio of *m*/*z* 617/600 can be used to assign saturated fatty acids either to *sn-*2 or *sn-*1/3 positions. In addition, we observed that diacylglycerol fragments with *sn-*1/3 losses are preferred over *sn-*2 losses, creating dominant *sn-*2 positional monoacylglycerol fragments (Figures S9–S78). However, these findings are affected by the stoichiometry of attached fatty acids.

### General Screening of Acylglycerol in Linseed Oil by SFC-APPI-CID

**Figure 6:**
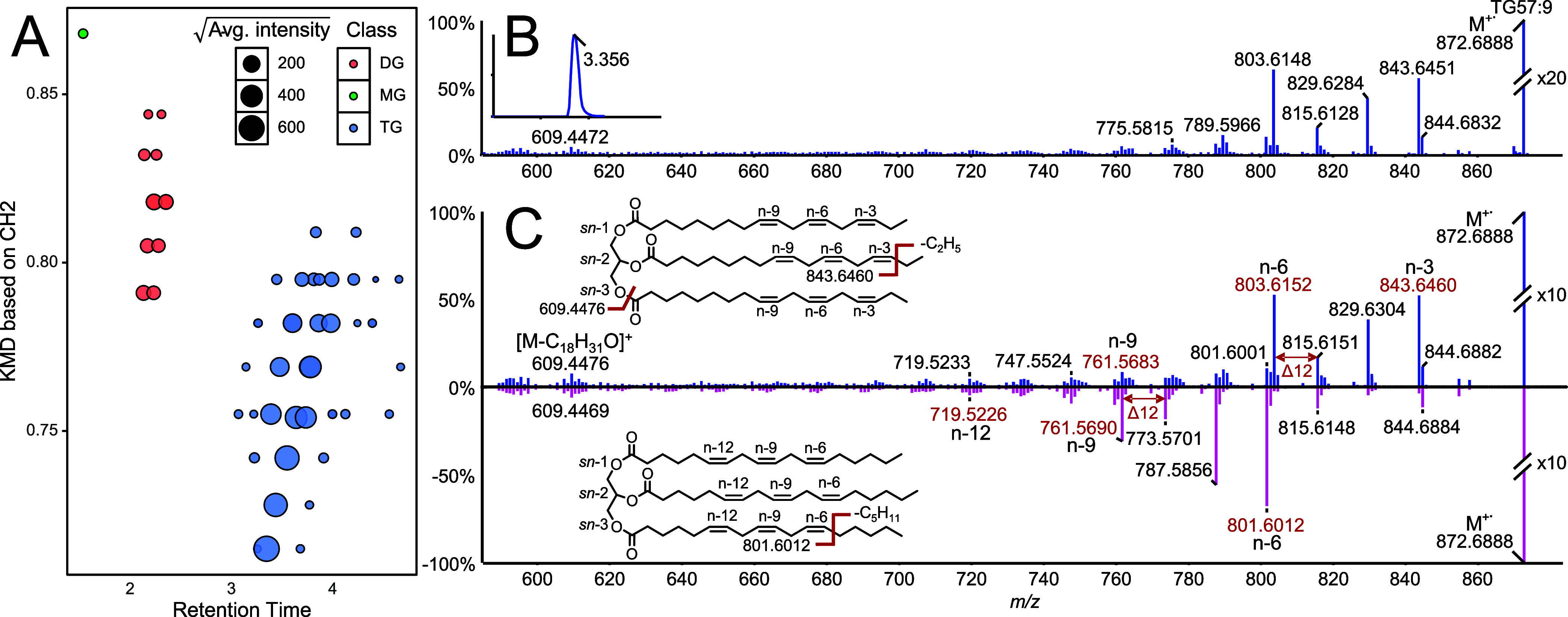
(A) Kendricks mass defect plot of acylglycerols (*n* = 57*) found in linseed oil and (B) 10–70 eV CID spectra of LnLnLn in linseed oil compared to (C) two 40 eV spectra of isomeric triglycerides with LnLnLn (blue) and gLngLngLn (pink).*47 out of 57 acylglycerols were sufficiently separated by chromatography to evaluate retention times.

Linseed oil consists of average of about 90% unsaturated fatty acids^29^ and is ideal for benchmarking the capability of EDP-CID for the analysis of acylglycerols with a focus on DB annotation and SFC-ESI-CID for the confirmation of lipid class and fatty acid attachment. Major fatty acid species reported after saponification and esterification of acylglycerol species are unsaturated fatty acids FA18:3 (57.2%), FA18:1 (17.7%), FA18:2 (14.5%), as well as saturated fatty acids FA18:0 (4.0%) and FA16:0 (5.6%).^29^ GC-EI analysis and reference standard matching indicated that the most common DB positions for FA18:3 are n-3,n-6,n-9, for FA18:2 n-6,n-9 and for FA18:1 n-9.^30^ Using SFC-HRMS along with APPI (Figure S79), 47 putative acylglycerols in linseed oil are identified based on retention time and exact mass of ED ions and with the equivalent carbon number chromatographic behavior (Figure 6A, Table S5, Figure S80). SFC-ESI-MS/MS experiments of these 47 putative acylglycerols demonstrated the presence of 10 additional acylglycerol isomers, which were not completely separated by chromatography. Among the 57 acylglycerols, 44 are triglycerides, 12 are diglycerides, and one is a monoglyceride. Correct lipid class annotation was confirmed based on fatty acid fragments and water losses of SFC-ESI-MS/MS experiments. For SFC-APPI/MS/MS with DDA, product ion spectra were recorded for 47 acylglycerols. Isomeric acylglycerols, which were not sufficiently separated by chromatography (10) yielded composite spectra. Full DB annotation was performed on 33 acylglycerols and partial annotation on 6 acylglycerols (Table S5). Incomplete or missing assignments are related to low-intensity DDA product ion spectra, complex composite spectra of coeluting isomeric acylglycerols, or dominant isotopic contributions from other acylglycerols. The de novo annotation results of the linseed oil sample shows that the relative distribution of fatty acids attached to acylglycerols is 39% for FA18(n-3,n-6,n-9), followed by FA18:1(n-9) (22%), FA18:2(n-6,n-9) (18%), FA16:0 (12%) and less than 3% for the remaining fatty acids with the most abundant acylglycerol being LnLnLn. These findings are in alignment with the distribution of fatty acids found in linseed oil by using GC-EI of acylglycerols after saponification and derivatization but do not represent quantitative results.^29,30^ Confidence in acylglycerol identification can be increased by comparing denovo annotated EDP-CID spectra, such as LnLnLn (Figure 6B) to EDP-CID spectra of isomeric reference standards (Figure 6C). The two isomers gLngLngLn and LnLnLn (Figure 6C) show EDP-CID spectra that allow differentiation of DB positions n-3,n-6,n-9 from Ln and with positions n-6,n-9,n-12 from gLn and thereby validate the presence of LnLnLn in the linseed oil sample.

## Conclusions

Chlorobenzene-assisted APPI-MS selectively ionizes unsaturated acylglycerols as electron-deficient ions (M^+.^ and [M-H]^+^), whereas saturated acylglycerols dissociate in-source to fatty acid, monoacylglycerol, and diacylglycerol fragments. EDP-CID of acylglycerols yields information-rich fragmentation spectra with distinct intensity ratios for *sn*-positional isomers and multiple fragmentation series related to the DB position of acylglycerols. The most common double-bond related EFL series starts from the precursor with successive losses of CH_3_(CH_2_)*_n_* units for the first DB and additional Δ2*H* for each following DB (M-CH_3_(CH_2_)*_n_* Δ2H_DB-1_). For additional fragmentation series, DB positions can be spotted by defining the start of the series (e.g., after fatty acid loss, M-FA-CH_3_(CH_2_)*_n_*) and spotting the corresponding intensity maxima. Acylglycerols with mixed unsaturated fatty acid chains do generate overlapping fragmentation series, with one intensity maxima per unique DB position. To facilitate de novo structural elucidation and overcome time-consuming manual annotation of DBs, we developed MsRadaR and R package with integrated data-processing workflows for data interpretation and visualization. Based on EFLs, relevant series can be extracted, and DB positions are assigned using intensity and intensity slope maxima peak picking algorithms. Last, SFC-APPI-EDP-CID was integrated into standard SFC-ESI workflows for the general screening, structural elucidation, and separation of acylglycerol isomers in linseed oil in less than 5 min. This allowed the complete DB annotation of 33 putative acylglycerols. Certainly, the compatibility of SFC with ESI and APPI enables the characterization and quantification of acylglycerols on almost any type of mass analyzer. Furthermore, due to limited availability of reference analytes, chromatographic retention (equivalent carbon number) was applied to eliminate false positives and could be further used for structural identification of low-abundant analytes. While, data-dependent acquisition is regularly used for the general screening of samples, the limited availability of data points challenges the S/N ratio required for reliable annotation of double bonds for low-abundant and coeluting lipids using EDP-CID, alike other double-bond resolving techniques. For better coverage, it is recommended to perform preceding ESI-CID experiments to distinguish the fatty acid composition followed by targeted EDP-CID experiments for double bond annotation. This will enable performing dynamic background subtraction in MS2 to mitigate the contribution of coeluting lipids. Ongoing work explores the potential of EDP-CID for other lipid classes, untargeted screening methods, and fully automated processing workflows for EDP-CID data.

## Data Availability

Supporting files are available at 10.26037/yareta:k4kzc7xp4bdfrhlhjtoflmb5au.

## Supporting Information Available

List of analytes, MS-Source conditions, MS settings, APPI-MS, APPI-MS/MS spectra and corresponding extracted series, and linseed oil chromatograms and selected MS examples from linseed oil (PDF)
