# Supplementary material for: De Novo Structural Elucidation of Acylglycerols by Supercritical Fluid Chromatography and Collision-Induced Dissociation of Electron-Deficient Precursor Ions
Source: Anal Chem. 2025 Feb 7;97(6):3600–7. doi: 10.1021/acs.analchem.4c05976 (PMC11840804; doi:10.1021/acs.analchem.4c05976)
Supplement: Supplementary file 1 — ac4c05976_si_001.pdf [file ac4c05976_si_001.pdf]

Supporting Information

De-Novo Structural Elucidation of Acylglycerols by Supercritical Fluid Chromatography and Collision-Induced Dissociation of Electron Deficient Precursor Ions

Patrick Mueller and Gérard Hopfgartner*

Life Sciences Mass Spectrometry, Department of Inorganic and Analytical Chemistry, University of Geneva, 24 Quai Ernest Ansermet, CH-1211 Geneva 4, Switzerland

*corresponding author at e-mail:gerard.hopfgartner@unige.ch

Orcid: Patrick Mueller 0000-0003-1597-0299

Orcid: Gérard Hopfgartner 0000-0002-9087-606X

Short Title: Structural elucidation of double bonds position in acylglycerols

Keywords: SFC-MS, atmospheric pressure photoionization, radical cation, collision induced dissociation, acylglycerols

Table of Content

Table S1: List of acylglycerol standards analyzed.

Table S2: ESI and APPI ion source settings and APPI probe parameters.

Table S3: De-novo structural elucidation results of acylglycerols in linseed oil.

Figure S1: log2 Ratio of Radical Cation ($M^{+\cdot}$) vs $[M-H]^+$ of acylglycerols with at least 1 double bond using SFC-APPI. Deisotoping was performed for $M^{+\cdot}$ intensities.

Figure S2: Contribution of radical cations and $[M-H]^+$ to the total ion current using SFC-APPI. Deisotoping was performed for $M^{+\cdot}$ intensities.

Figure S3-S8: SFC-APPI-MS1 and SFC-ESI-MS1 spectra of acylglycerol standards.

Figure S9-S78: Annotated EDP-CID results of acylglycerol standards using MsRadaR.

Figure S79: Total ion chromatograms of the 3 linseed oil replicates.

Figure S80: Extracted ion chromatograms of selected di-and triglycerides found in linseed oil (replicate 1).

Table S1: List of acylglycerol standards analyzed.

Analyte	Class	Elemental Formula	Composition	Double Bond Position	Supplier
Tritridecanoin	Triacylglycerol	C42H80O6	TG(13:0/13:0/13:0)	-	Sigma-Aldrich
Trinonadecanoin	Triacylglycerol	C60H116O6	TG(19:0/19:0/19:0)	-	Sigma-Aldrich
Tricaprin	Triacylglycerol	C33H62O6	TG(10:0/10:0/10:0)	-	Sigma-Aldrich
Trilinolein	Triacylglycerol	C57H98O6	TG(18:2/18:2/18:2)	n-6,n-9	Larodan
Triolein	Triacylglycerol	C57H104O6	TG(18:1/18:1/18:1)	n-9	Supelco
1,2-Dipalmitoyl-3-Linoleoyl-rac-glycerol	Triacylglycerol	C53H98O6	TG(16:0/16:0/18:2)	n-6,n-9	Cayman Chemical
1,3-Dipalmitoyl-2-Linoleoyl-rac-glycerol	Triacylglycerol	C53H98O6	TG(16:0/18:2/16:0)	n-6,n-9	Cayman Chemical
1,2-Distearoyl-3-Linoleoyl-rac-glycerol	Triacylglycerol	C57H106O6	TG(18:0/18:0/18:2)	n-6,n-9	Cayman Chemical
1,3-Distearoyl-2-Linoleoyl-rac-glycerol	Triacylglycerol	C57H106O6	TG(18:0/18:2/18:0)	n-6,n-9	Cayman Chemical
1,2-Dilinoleoyl-3-γ-Linolenoyl-rac-glycerol	Triacylglycerol	C57H96O6	TG(18:2/18:2/18:3)	n-6,n-9,n-12	Cayman Chemical
1,2-Dilinoleoyl-3-α-Linolenoyl-rac-glycerol	Triacylglycerol	C57H96O6	TG(18:2/18:2/18:3)	n-3,n-6,n-9,n-15	Cayman Chemical
Glycerol Tri-α-Linolenoyl	Triacylglycerol	C57H92O6	TG(18:3/18:3/18:3)	n-3,n-6,n-9	Cayman Chemical
Glycerol Tri-γ-Linolenoyl	Triacylglycerol	C57H92O6	TG(18:3/18:3/18:3)	n-6,n-9,n-12	Cayman Chemical
1,3-Dilinoleoyl-2-Stearoyl Glycerol	Triacylglycerol	C57H106O6	TG(18:2/18:0/18:2)	n-6,n-9	Cayman
1-Oleoyl-2-Palmitoyl-3-Linoleoyl-rac-glycerol	Triacylglycerol	C55H100O6	TG(18:1/16:0/18:2)	n-6,n-9	Cayman
1-Palmitin-2-Linolein-3-Olein,	Triacylglycerol	C55H100O6	TG(16:0/18:2/18:1)	n-6,n-9	Larodan Lipids
1-Palmitoyl-2-oleoyl-3-linoleoyl-rac-glycerol	Triacylglycerol	C55H100O6	TG(16:0/18:1/18:2)	n-6,n-9	Cayman Chemical
1,3-Palmitolein-2-Olein	Triacylglycerol	C53H96O6	TG(16:1/18:1/16:1)	n-7,n-9	Larodan Lipids
1,2-Palmitolein-3-Olein	Triacylglycerol	C53H96O6	TG(16:1/16:1/18:1)	n-7,n-9	Larodan Lipids
1,3-Dioleoyl-2-Stearoyl Glycerol	Triacylglycerol	C57H106O6	TG(18:1/18:0/18:1)	n-9	Cayman
1,3-Dipalmitoyl-2-Arachidonoyl-sn-glycerol	Triacylglycerol	C55H98O6	TG(16:0/20:4/16:0)	n-6,n-9,n-12,n-15	Cayman Chemical
1,3-Dilinoleoyl Glycerol	Diacylglycerol	C39H68O5	DG(18:2/OH/18:2)	n-6,n-9	Cayman
1,2-Dilinoleoyl-sn-glycerol	Diacylglycerol	C39H68O5	DG(18:2/18:2/OH)	n-6,n-9	Cayman
1,3-Diarachidonoyl Glycerol	Diacylglycerol	C43H68O5	DG(20:4/OH/20:4)	n-6,n-9,n-12,n-15	Cayman
1,3-Distearoyl Glycerol	Diacylglycerol	C39H76O5	DG(18:0/OH/18:0)	-	Cayman
Diolein	Diacylglycerol	C39H72O5	DG(18:1/OH/18:1)	n-9	Cayman Chemical
1-Oleoyl-2-Linoleoyl-rac-glycerol	Diacylglycerol	C39H70O5	DG(18:1/18:2/OH)	n-6,n-9	Cayman Chemical
1-Stearoyl-3-Linoleoyl-rac-glycerol	Diacylglycerol	C39H72O5	DG(18:0/OH/18:2)	n-6,n-9	Cayman Chemical
2-Arachidonyl glycerol	Monoacylglycerol	C23H38O4	MG(OH/20:4/OH)	n-6,n-9,n-12,n-15	Cayman Chemical
2-Oleoyl Glycerol	Monoacylglycerol	C21H40O4	MG(OH/18:1/OH)	n-9	Cayman Chemical
1-Oleoyl Glycerol	Monoacylglycerol	C21H40O4	MG(18:1/OH/OH)	n-9	Sigma-Aldrich
1-Stearoyl-rac-glycerol	Monoacylglycerol	C21H42O4	MG(18:0/OH/OH)	-	Cayman Chemical
2-Linoleoyl Glycerol	monoacylglycerol	C21H38O4	MG(OH/18:2/OH)	n-6,n-9	Cayman Chemical

Table S2: ESI and APPI ion source settings and APPI probe parameters.

	SFC-ESI		SFC-APPI
**CUR**	25	25	25
**GS1**	30	30	70
**GS2**	35	35	40
**ISVF**	4500 V	5000 V	1600 V
**TEM**	50°C	50°C	400°C

**APPI: probe and lamp positions**	
**UV lamp vertical position**	0.27 µm
**APCI probe vertical position**	11.521 µm
**APCI probe horizontal position**	4.51 µm

Table S3: MS1 and MS2 experiment conditions.

Experimental Conditions - Standards				
	MS1		MS2	
**Mass Range**	150	1000	30	1000
**Resolution**	30.000		30.000	
**MS/MS Isolation width**	-		1 unit	
**Experimental conditions – Linseed oil**				
	MS1		MS2	
**Mass Range**	150	1500	30	1500
**Resolution**	30.000	30.000	30.000	
**MS/MS Isolation width**	-		1 unit	

Table S4. Predicted and characteristic fragment ions for the annotation of double bonds. Cells marked in light blue are relevant for the interpretation of acylglycerol standards. Additional elemental formula losses can be calculated based on the following formulas: C= #C_{loss} and H = #C_{loss}*2+1-2*(#DB-1).

**Double Bond Information**		**1st Double Bond:**		**2nd Double Bond:**		**3rd Double Bond:**		**4th Double Bond:**	
DB Position	Carbon losses	Radical loss	Neutral loss (ΔH)	Radical loss (Δ2H)	Neutral loss (Δ3H)	Radical loss (Δ4H)	Neutral loss (Δ5H)	Radical loss (Δ6H)	Neutral loss (Δ 7H)
n-3	2	-C_2H_5 29.0386	-C_2H_4 28.0313	-	-	-	-	-	-
n-6	5	-C_5H_{11} 71.0855	-C_5H_{10} 70.0783	-C_5H_9 69.0699	-C_5H_8 68.0626	-C_5H_7 67.0542	-C_5H_6 66.0470	-	-
n-7	6	-C_6H_{13} 85.1017	-C_6H_{12} 84.0939	-C_6H_{11} 83.0866	-C_6H_{10} 82.0793	-C_6H_9 81.07207	-C_6H_8 80.0648	-C_6H_7 79.0575	-C_6H_6 78.0502
n-9	8	-C_8H_{17} 113.1325	-C_8H_{16} 112.1252	-C_8H_{15} 111.1168	-C_8H_{14} 110.1096	-C_8H_{13} 109.1012	-C_8H_{12} 108.0939	-C_8H_{13} 107.0866	-C_8H_{12} 106.0793
n-12	11	-$C_{11}H_{21}$ 153.1638	-$C_{11}H_{20}$ 152.1565	-$C_{11}H_{19}$ 151.1481	-$C_{11}H_{18}$ 150.1409	-$C_{11}H_{17}$ 149.1325	-$C_{11}H_{16}$ 148.1252	-$C_{11}H_{17}$ 147.1179	-$C_{11}H_{16}$ 146.1107
n-15	14	-$C_{14}H_{29}$ 197.2269	-$C_{14}H_{28}$ 196.2191	-$C_{14}H_{27}$ 195.2113	-$C_{14}H_{26}$ 194.2035	-$C_{14}H_{25}$ 193.1956	-$C_{14}H_{24}$ 192.1878	-$C_{14}H_{23}$ 191.1800	-$C_{14}H_{22}$ 190.1722

Table S5. De-novo structural elucidation results of acylglycerols in linseed oil.

No	Species	*m/z*	Elemental Formula	Average Retention time	Average Intensity	Dominant Lipid	Double bond positions	Coeluting Lipid
1	MG18:3	352.2614	C21H36O4	1.54	1290	MG18:3	Poor MS/MS Quality	-
2	DG34:3	590.4910	C37H66O5	2.14	4671	DG18:3_16:0	FA18:3: n-3,n-6,n-9	-
3	DG34:3	590.4910	C37H66O5	2.26	2917	DG18:3_16:0	FA18:3: n-3,?,?	-
4	DG34:2	592.5056	C37H68O5	2.18	972	DG(18:2_16:0)	FA18:2: n-6,n-9	-
5	DG34:2	592.5056	C37H68O5	2.31	800	DG(18:2_16:0)	FA18:2: n-6,n-9	-
6	DG36:6	612.4754	C39H64O5	2.13	20810	DG18:3_18:3	FA18:3: n-3,n-6,n-9	-
7	DG36:6	612.4754	C39H64O5	2.23	14016	DG18:3_18:3	FA18:3: n-3,n-6,n-9	-
8	DG36:5	614.4910	C39H66O5	2.17	15565	DG18:3_18:2	FA18:3: n-3,n-6,n-9, FA18:2: n-6,n-9	-
9	DG36:5	614.4910	C39H66O5	2.28	11740	DG18:3_18:2	No MS/MS for APPI	-
10	DG36:4	616.5067	C39H68O5	2.35	19851	DG18:3_18:1	FA18:3: n-3,n-6,n-9, FA18:1: n-9	DG18:2_18:2
11	DG36:4	616.5067	C39H68O5	2.24	29274	DG18:3_18:1	FA18:3: n-3,n-6,?, FA18:1: n-9?	DG18:2_18:2
12	DG36:4	616.5067	C39H68O5	2.24	29274	DG18:2_18:2	FA18:2: n-6,n-9	DG18:3_18:1
13	DG36:4	616.5067	C39H68O5	2.35	19851	DG18:2_18:2	FA18:2: n-6,n-9	DG18:3_18:1
14	TG50:6	822.6737	C53H90O6	3.07	807	TG18:3_18:3_14:0	FA18:3: n-3,n-6,n-9	-
15	TG50:5	824.6894	C53H92O6	3.14	514	TG18:3_18:2_14:0	No MS/MS for APPI	-
16	TG50:4	826.7050	C53H94O6	3.26	684	TG18:3_16:1_16:0	FA18:3: n-3,n-6,?, FA16:1: n-7	TG18:3_14:0_18:1
17	TG50:4	826.7050	C53H94O6	3.26	684	TG18:3_14:0_18:1	FA18:3: ?, FA18:1: n-9	TG18:3_16:1_16:0
18	TG50:3	828.7207	C53H96O6	3.45	2183	TG18:3_16:0_16:0	FA18:3: n-3,n-6,n-9	-
19	TG51:6	836.6894	C54H92O6	3.22	461	TG18:3_18:3_15:0	FA18:3: n-3,n-6,n-9	-
20	TG52:7	848.6894	C55H92O6	3.23	1966	TG18:3_18:3_16:1	FA18:3: n-3,n-6,n-9, FA16:1: n-9	-
21	TG52:6	850.7050	C55H94O6	3.39	124334	TG18:3_18:3_16:0	FA18:3: n-3,n-6,n-9	-
22	TG52:5	852.7207	C55H96O6	3.48	84303	TG18:3_18:2_16:0	FA18:3: n-3,n-6,n-9, FA18:2: n-6,n-9	-
23	TG52:4	854.7363	C55H98O6	3.60	78055	TG18:3_18:1_16:0	FA18:3: n-3,n-6,n-9, FA18:1: n-9	TG18:2_18:2_16:0
24	TG52:4	854.7363	C55H98O6	3.60	78055	TG18:2_18:2_16:0	FA18:2: n-6,n-9	TG18:3_18:1_16:0
25	TG52:3	856.7520	C55H100O6	3.81	8302	TG18:1_18:1_18:1	FA18:1: n-9	-
26	TG52:3	856.7520	C55H100O6	3.70	13537	TG18:2_18:1_16:0	FA18:2: n-6,n-9, FA18:1: n-9	-
27	TG52:2	858.7676	C55H102O6	3.83	2880	TG18:1_18:1_16:0	FA18:1: n-9	-
28	TG53:9	858.6737	C56H90O6	3.26	433	TG53:9	No MS/MS for APPI	-
29	TG54:9	872.6894	C57H92O6	3.35	389638	TG18:3_18:3_18:3	FA18:3: n-3,n-6,n-9	-
30	TG54:8	874.7050	C57H94O6	3.44	255978	TG18:3_18:3_18:2	FA18:3: n-3,n-6,n-9, FA18:2: n-6,n-9	-
31	TG54:7	876.7207	C57H96O6	3.55	303105	TG18:3_18:3_18:1	FA18:3: n-3,n-6,n-9, FA18:1: n-9	-
32	TG54:6	878.7352	C57H98O6	3.64	186475	TG18:3_18:2_18:1	FA18:3: n-3,n-6,n-9, FA18:2: n-6,n-9, FA18:1: n-9	-
33	TG54:6	878.7352	C57H98O6	3.73	148055	TG18:3_18:3_18:0	No MS/MS for APPI	-

34	TG54:5	880.7520	C57H100O6	3.78	171620	TG18:2_18:2_18:1	FA18:2: n-6,n-9, FA18:1: n-9	TG(18:3_18:1_18:1)
35	TG54:5	880.7520	C57H100O6	3.78	171620	TG(18:3_18:1_18:1)	FA18:3: n-3,n-6,n-9, FA18:1: n-9	TG18:2_18:2_18:1
36	TG54:4	882.7676	C57H102O6	3.86	48280	TG18:2_18:1_18:1	FA18:2: n-6,n-9, FA18:1: n-9	-
37	TG54:4	882.7676	C57H102O6	3.98	71682	TG(18:3_18:1_18:0)	FA18:3: n-3,n-6,n-9, FA18:1: n-9	-
38	TG54:3	884.7833	C57H104O6	3.87	4341	TG(18:1/18:1/18:1)	FA18:1: n-9	-
39	TG54:3	884.7833	C57H104O6	3.99	16067	TG(18:1/18:2/18:0)	Poor MS/MS Quality	-
40	TG54:3	884.7833	C57H104O6	4.21	4683	TG(18:3_18:0_18:0)	Poor MS/MS Quality	-
41	TG54:2	886.7989	C57H106O6	4.23	1923	TG(18:1_18:1_18:0)	FA18:1: n-9	-
42	TG56:9	900.7207	C59H96O6	3.68	641	TG(18:3_18:3_20:3)	Complex Composite Spectra	Unknown
43	TG56:8	902.7363	C59H98O6	3.77	581	TG(18:3_18:2_20:3)?	Complex Composite Spectra	Unknown
44	TG56:7	904.7520	C59H100O6	3.91	1874	TG(18:3_18:3_20:1)	FA18:3: n-3,n-6,n-9, FA20:1: n-10	-
45	TG56:6	906.7676	C59H102O6	4.00	814	TG(18:2_18:3_20:1)	FA18:3: n-3,n-6,n-9, FA18:2: n-6,n-9, FA20:1: ?	TG(18:2_18:1_20:3)
46	TG56:6	906.7676	C59H102O6	4.00	814	TG(18:2_18:1_20:3)	FA20:3: n-3,n-6,n-9, FA18:2: n-6,n-9, FA18:1: ?	TG(18:2_18:3_20:1)
47	TG56:6	906.7676	C59H102O6	4.12	1153	TG(18:3_18:3_20:0)	FA18:3: n-3,n-6,n-9	-
48	TG56:4	910.7989	C59H106O6	4.39	667	TG(18:3_18:1_20:0)	Poor MS/MS Quality	TG(18:3_20:1_18:0)
49	TG56:4	910.7989	C59H106O6	4.24	277	TG(18:2_18:1_20:1)	Poor MS/MS Quality	-
50	TG56:4	910.7989	C59H106O6	4.39	667	TG(18:3_20:1_18:0)	Poor MS/MS Quality	TG(18:3_18:1_20:0)
51	TG56:3	912.8146	C59H108O6	4.42	185	TG(20:0_18:2_18:1)	No MS/MS for APPI	TG(20:0_18:2_18:1)<br>TG(20:2_18:1_18:0)<br>TG(20:1_18:2_18:0)<br>TG(22:0_18:3_16:0)
52	TG56:3	912.8146	C59H108O6	4.42	185	TG(20:2_18:1_18:0)	No MS/MS for APPI	
53	TG56:3	912.8146	C59H108O6	4.42	185	TG(20:1_18:2_18:0)	No MS/MS for APPI	
54	TG56:3	912.8146	C59H108O6	4.42	185	TG(22:0_18:3_16:0)	No MS/MS for APPI	
55	TG56:3	912.8146	C59H108O6	4.65	417	TG(20:0_18:3_18:0)	No MS/MS for APPI	-
56	TG58:6	934.7989	C61H106O6	4.56	650	TG(18:3_18:3_22:0)	FA18:3: n-3,n-6,n-9	-
57	TG58:5	936.8146	C61H108O6	4.67	430	TG(18:3_18:2_22:0)	No MS/MS for APPI	-

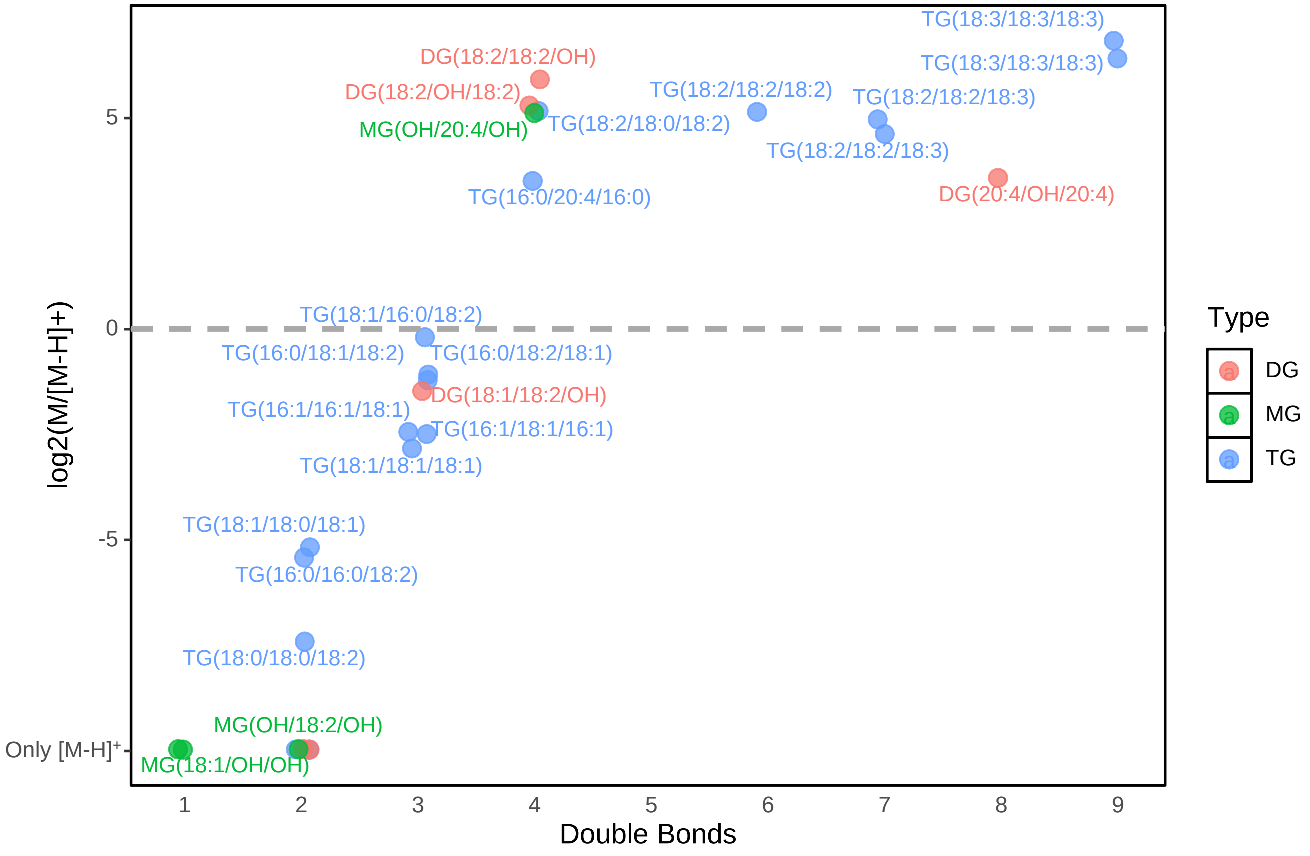

Figure S1: log2 Ratio of radical cation ($M^{+\cdot}$) vs $[M\text{-}H]^+$ of acylglycerols with at least 1 double bond using SFC-APPI. Deisotoping was performed for $M^{+\cdot}$ intensities.

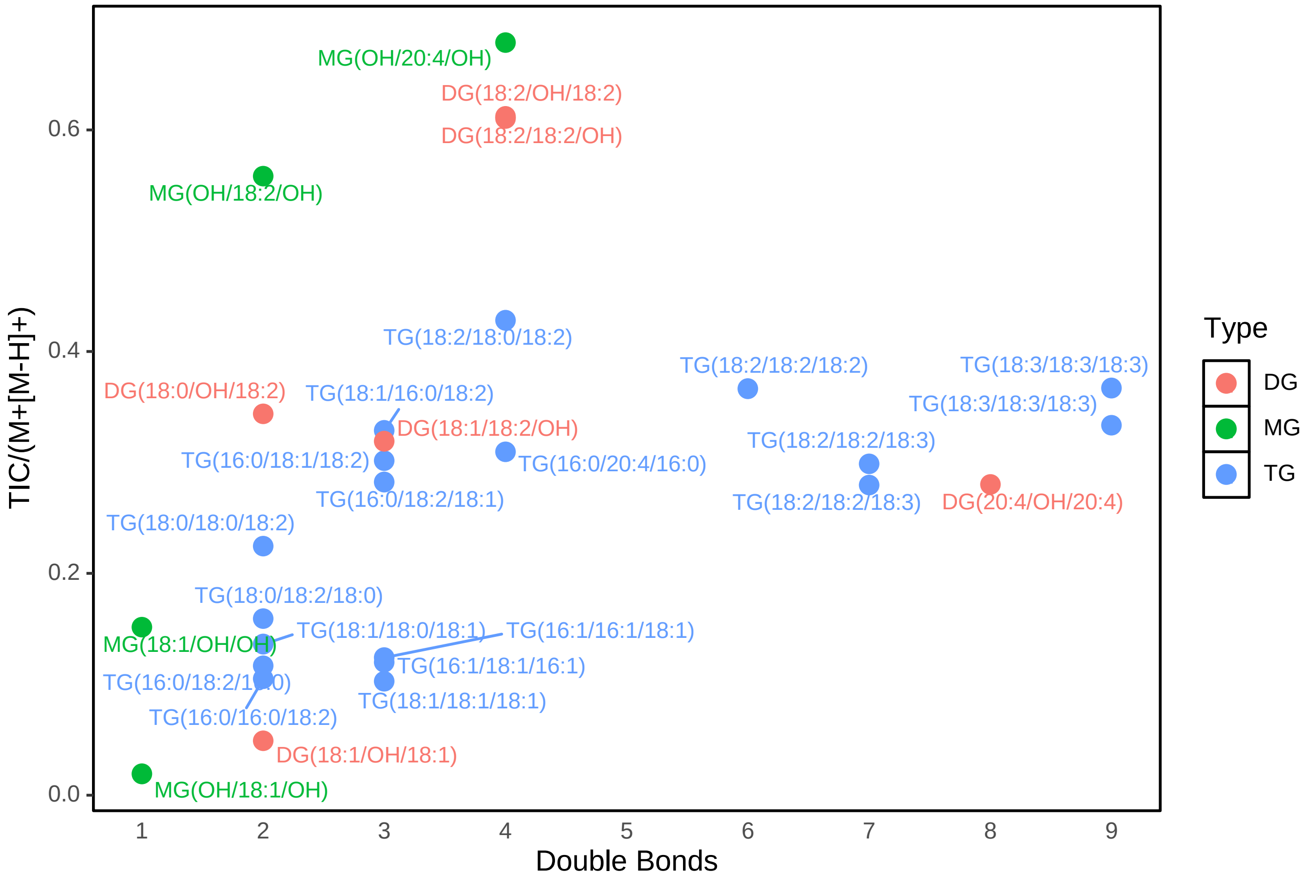

Figure S2: Contribution of radical cations and $[M-H]^+$ to the total ion current using SFC-APPI. Deisotoping was performed for $M^{+\cdot}$ intensities.

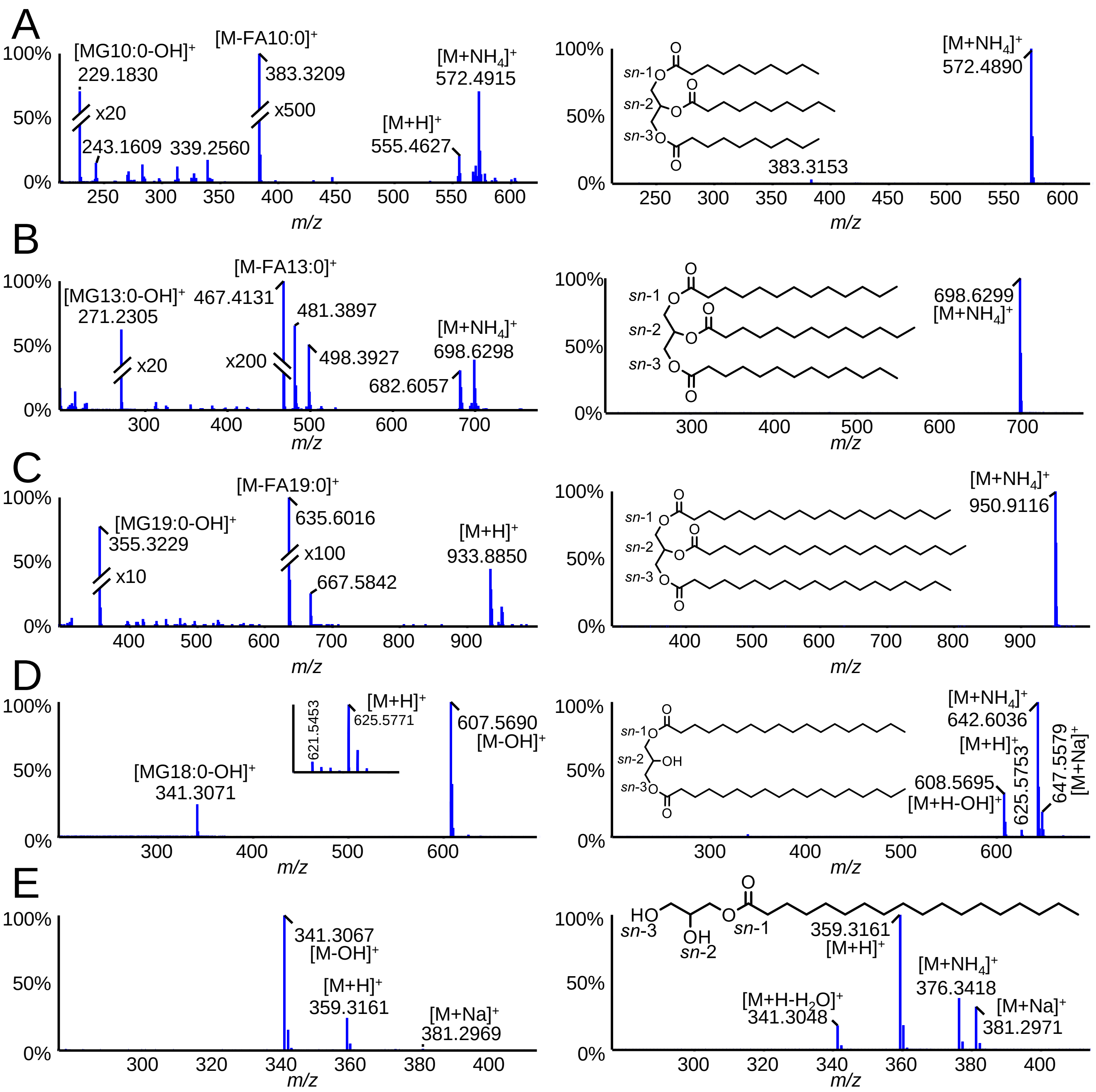

Figure S3: SFC-APPI (left panel) and SFC-ESI (right panel) MS1 spectra of saturated acylglycerols with A TG(10:0/10:0/10:0), B TG(13:0/13:0/13:0), C TG(19:0/19:0/19:0), D DG(18:0/OH/18:0) and E MG(18:0/OH/OH).

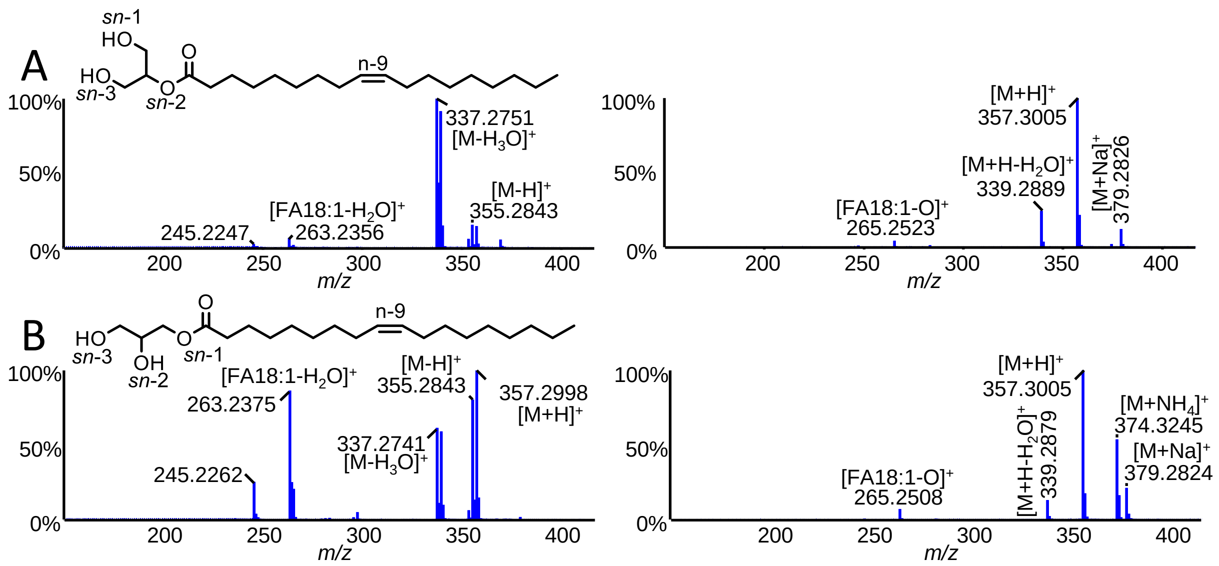

Figure S4: SFC-APPI (left panel) and SFC-ESI (right panel) MS1 spectra of monounsaturated monoglycerides with MG(OH/18:1/OH) and B MG(18:1/OH/OH).

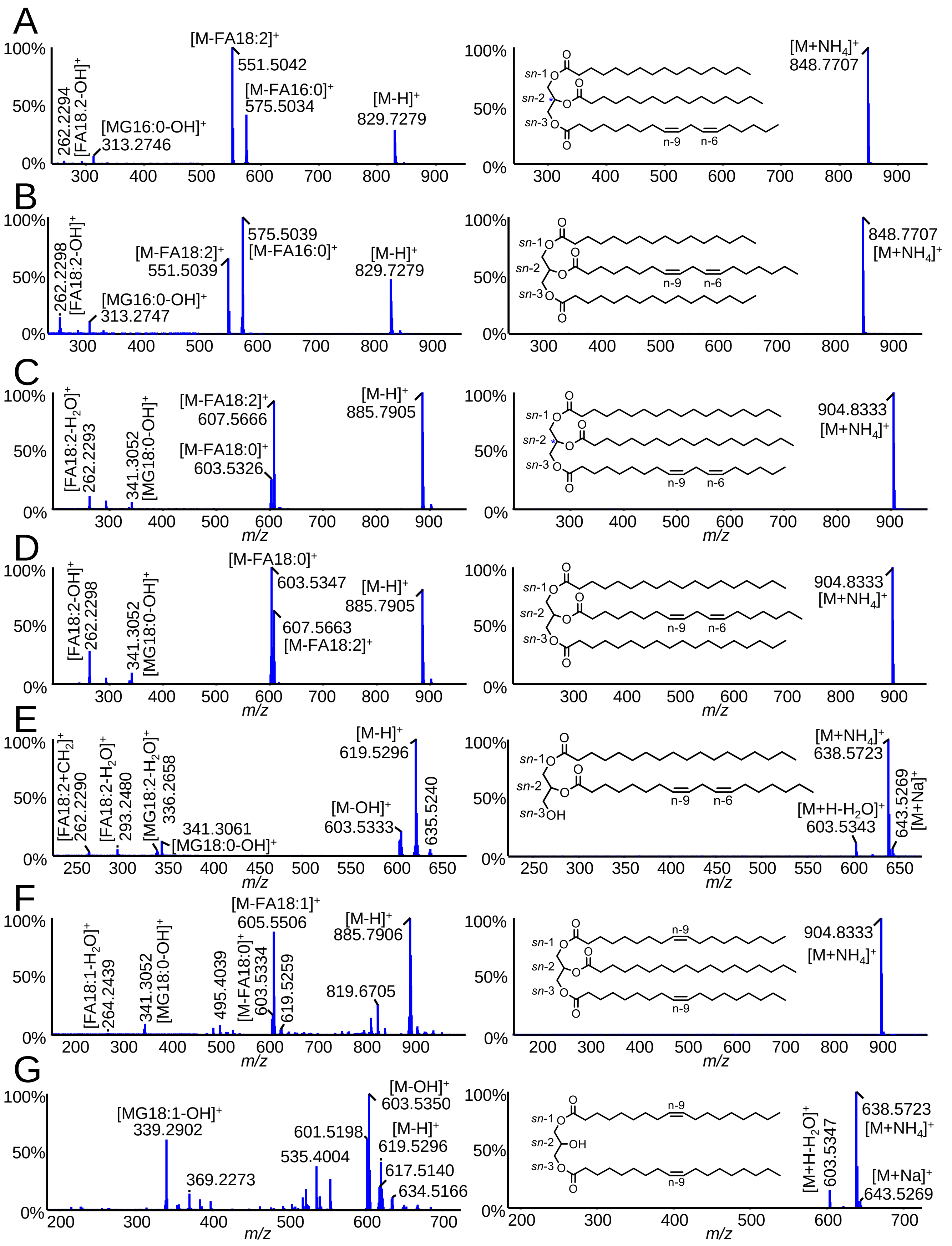

Figure S5: SFC-APPI (left panel) and SFC-ESI (right panel) MS1 spectra of acylglycerols carrying 2 double bonds with A TG(16:0/16:0/18:2), B TG(16:0/18:2/16:0), C TG(18:0/18:0/18:2), D TG(18:0/18:2/18:0), E DG(18:0/18:2/OH), F TG(18:1/18:0/18:1) and G DG(18:1/OH/18:1).

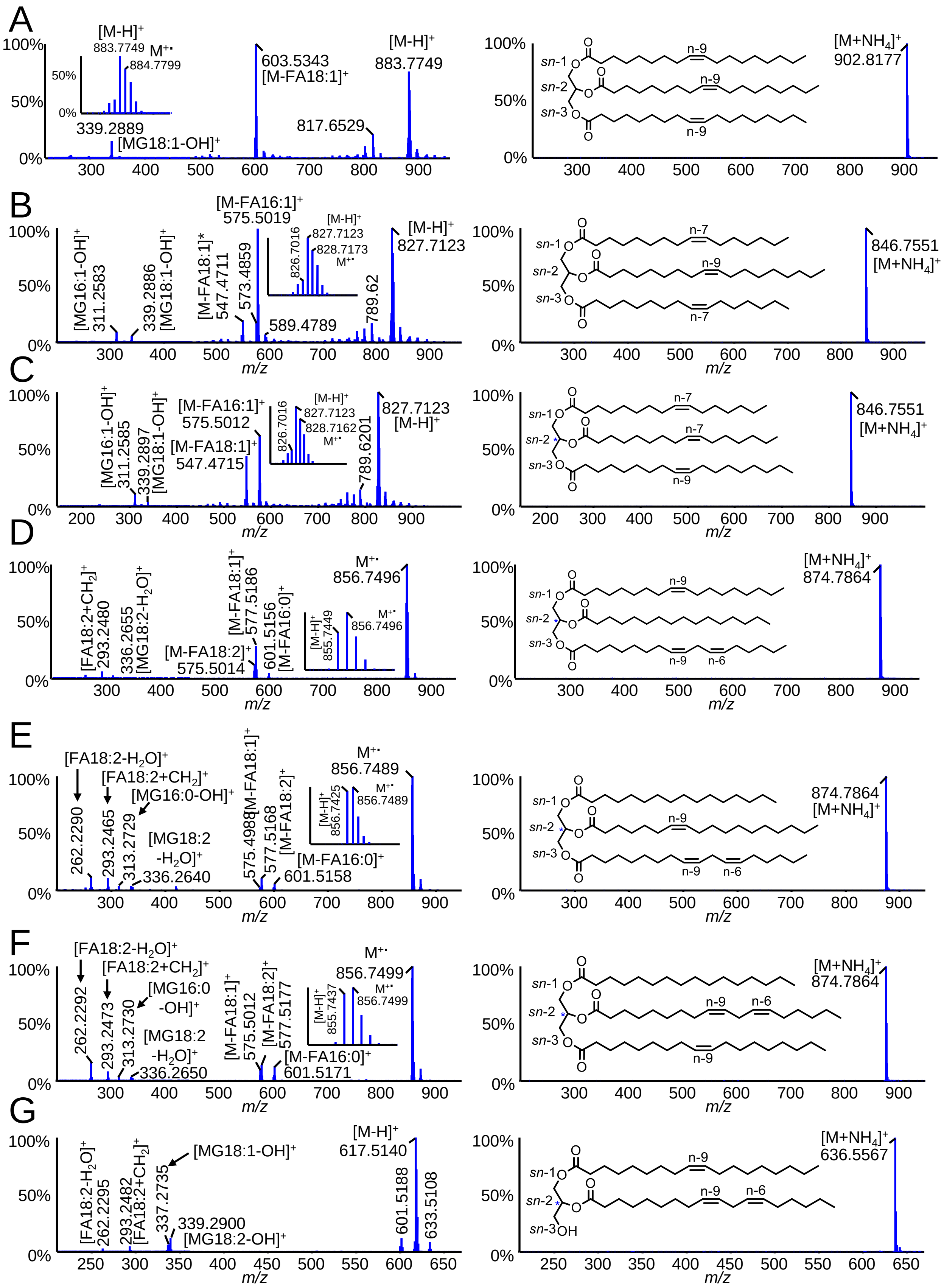

Figure S6: SFC-APPI (left panel) and SFC-ESI (right panel) MS1 spectra of acylglycerols carrying 3 double bonds with A TG(18:1/18:1/18:1), B TG(16:1/18:1/16:1), C TG(16:1/16:1/18:1), D TG(18:1/16:0/18:2), E TG(16:0/18:1/18:2), F TG(16:0/18:2/18:1) and G DG(18:1/18:2/OH).Insets are zoomed analyte ion regions from MS1 spectra.

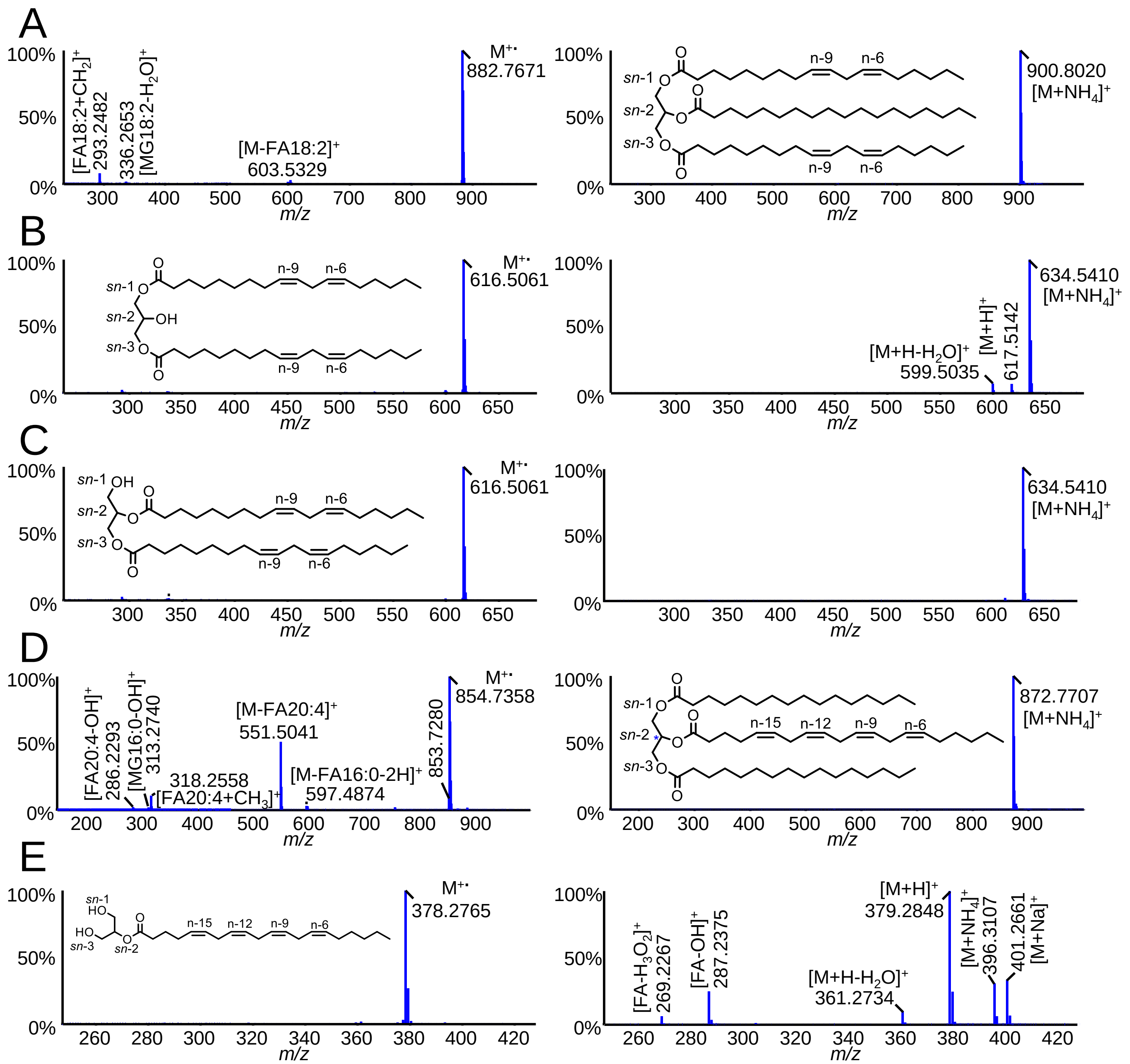

Figure S7: SFC-APPI (left panel) and SFC-ESI (right panel) MS1 spectra of acylglycerols carrying 4 double bonds with A TG(18:2/18:0/18:2), B DG(18:2/OH/18:2), C DG(OH/18:2/18:2), D TG(16:0/20:4/16:0) and E MG(OH/20:4/OH).

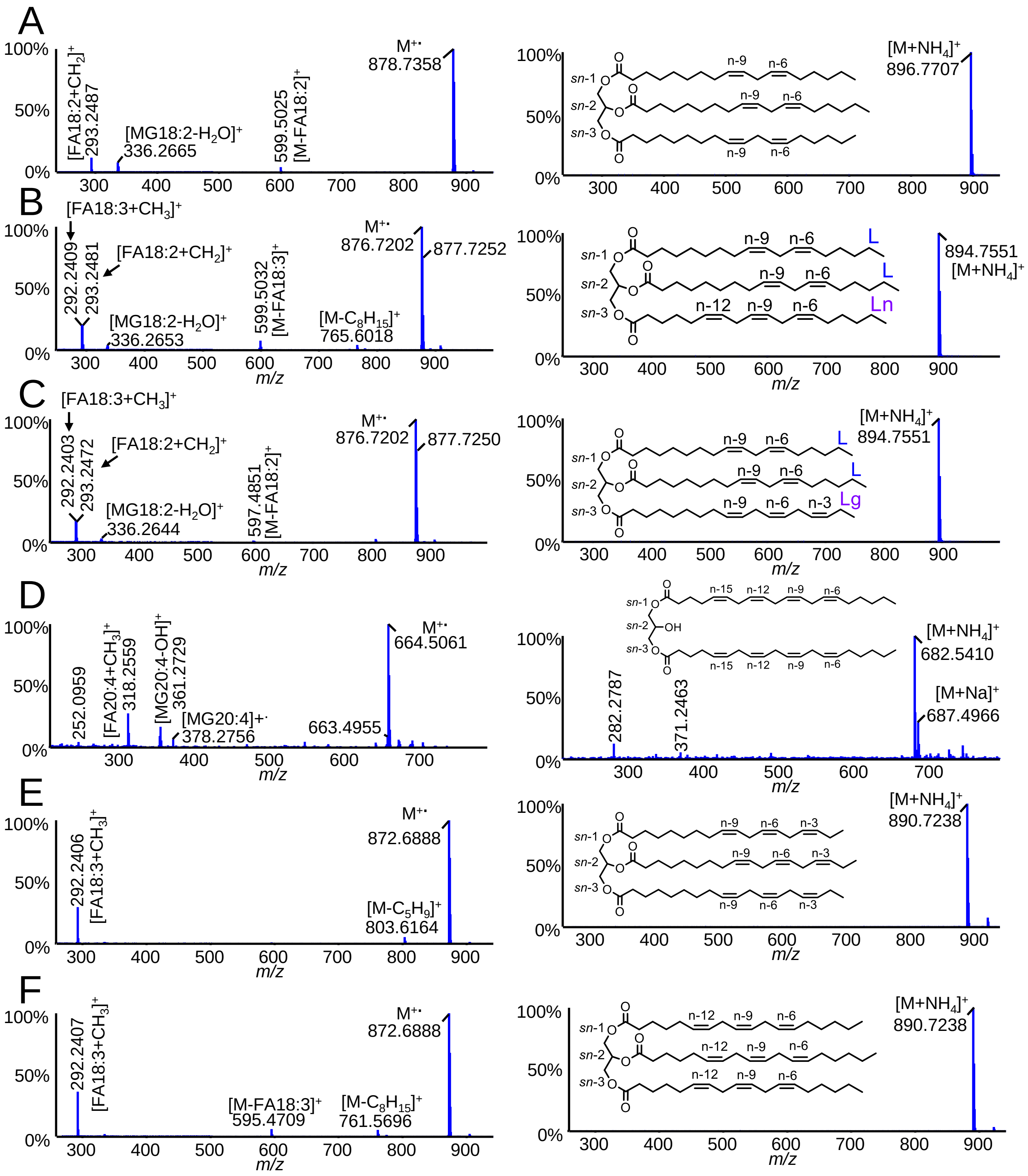

Figure S8: SFC-APPI (left panel) and SFC-ESI (right panel) MS1 spectra of acylglycerols carrying 6 to 9 double bonds with A TG(18:2/18:2/18:2), B TG(18:2/18:2/18:3), C TG(18:2/18:2/18:3), D DG(20:4/2OH/20:4), E TG(18:3/18:3/18:3) and F TG(18:3/18:3/18:3).

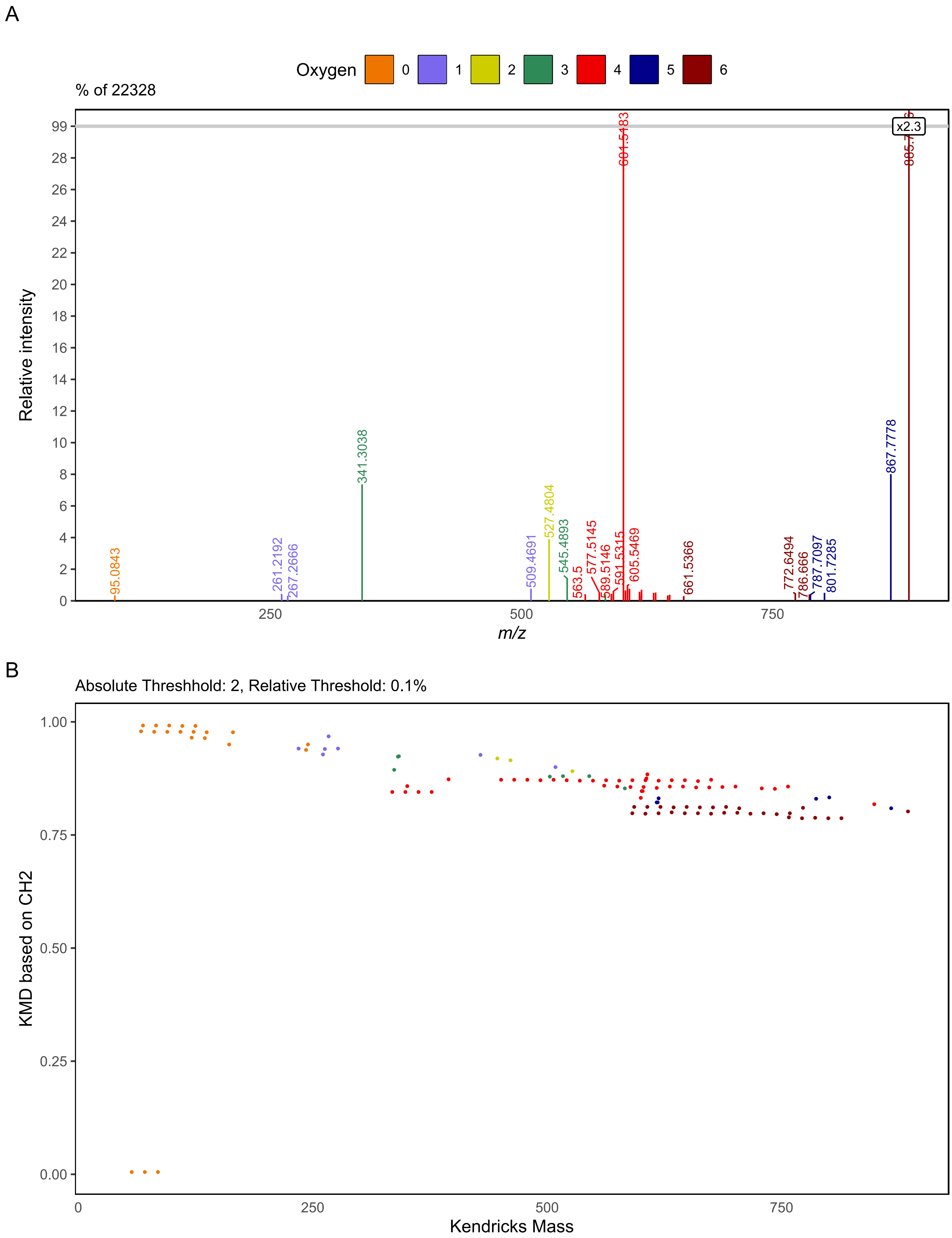

Figure S9: 35 eV CID of OSO $[M-H]^+$ with A) CID spectra and B) Kendricks plot from MsRadaR.

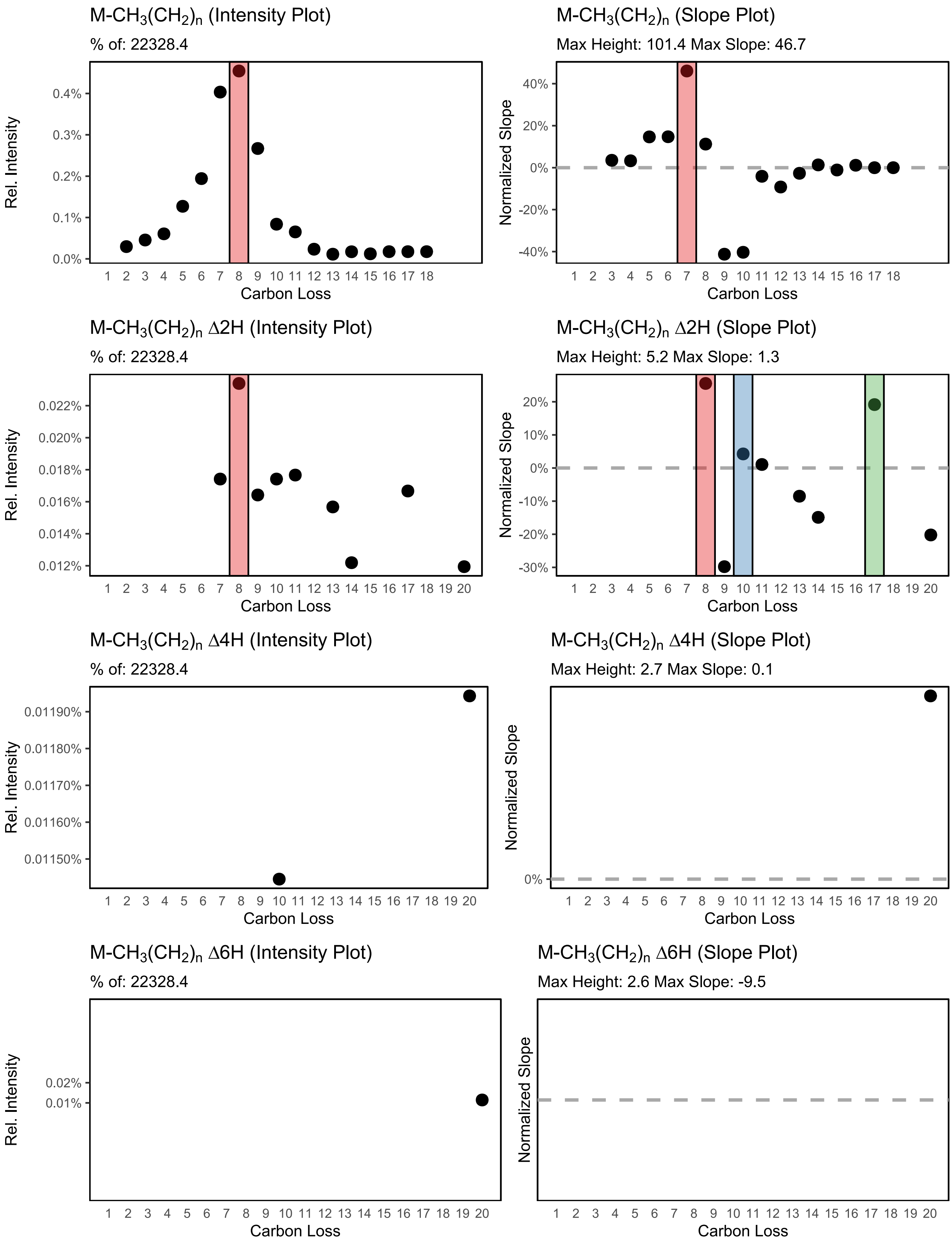

Figure S10: 35 eV CID of OSO $[M-H]^+$ with extracted fragmentation series starting from the precursor. Colored bars indicate intensity peak picking results from MsRadaR.

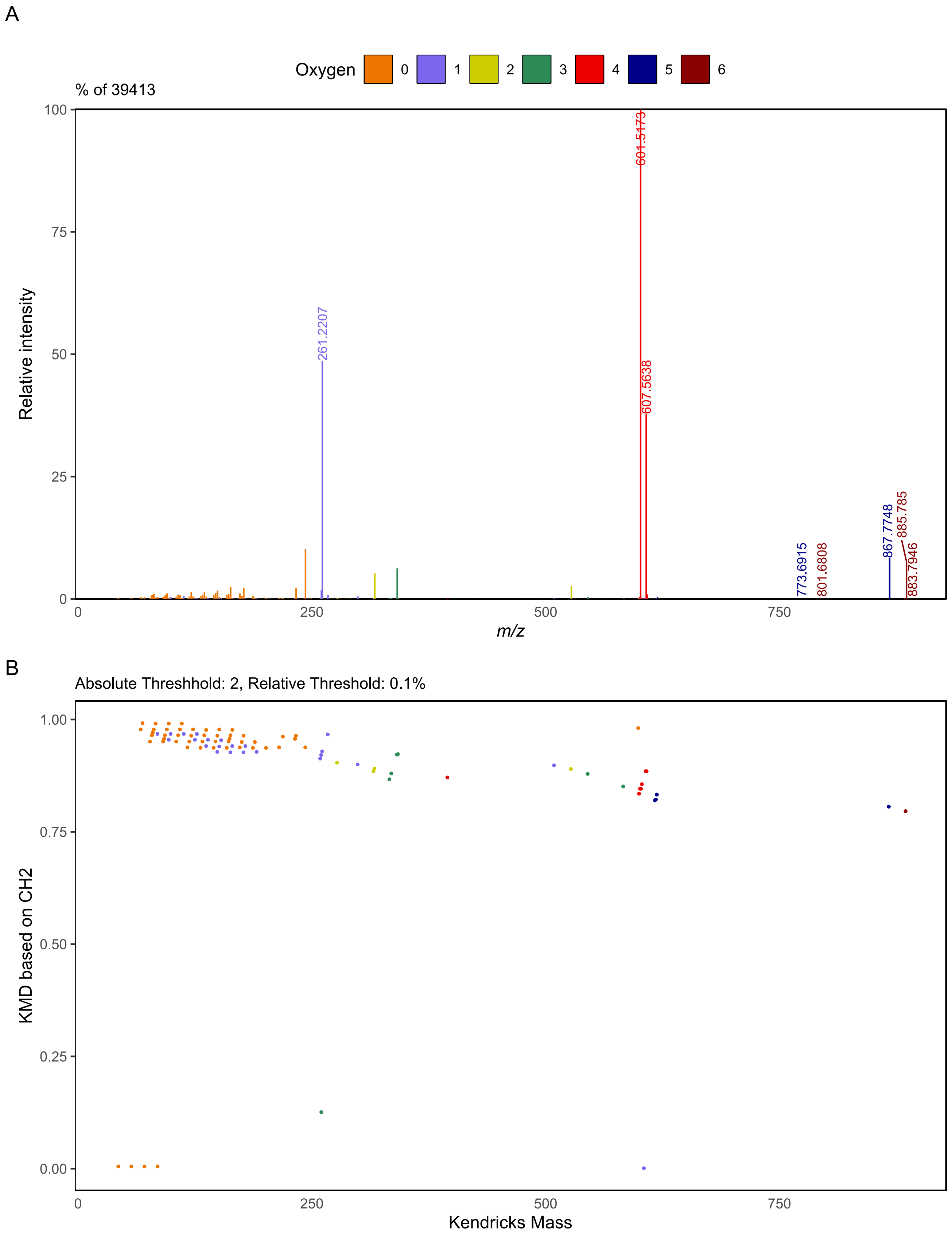

Figure S11: 35 eV CID of SLS $[M-H]^{+}$ with A) CID spectra and B) Kendricks plot from MsRadaR.

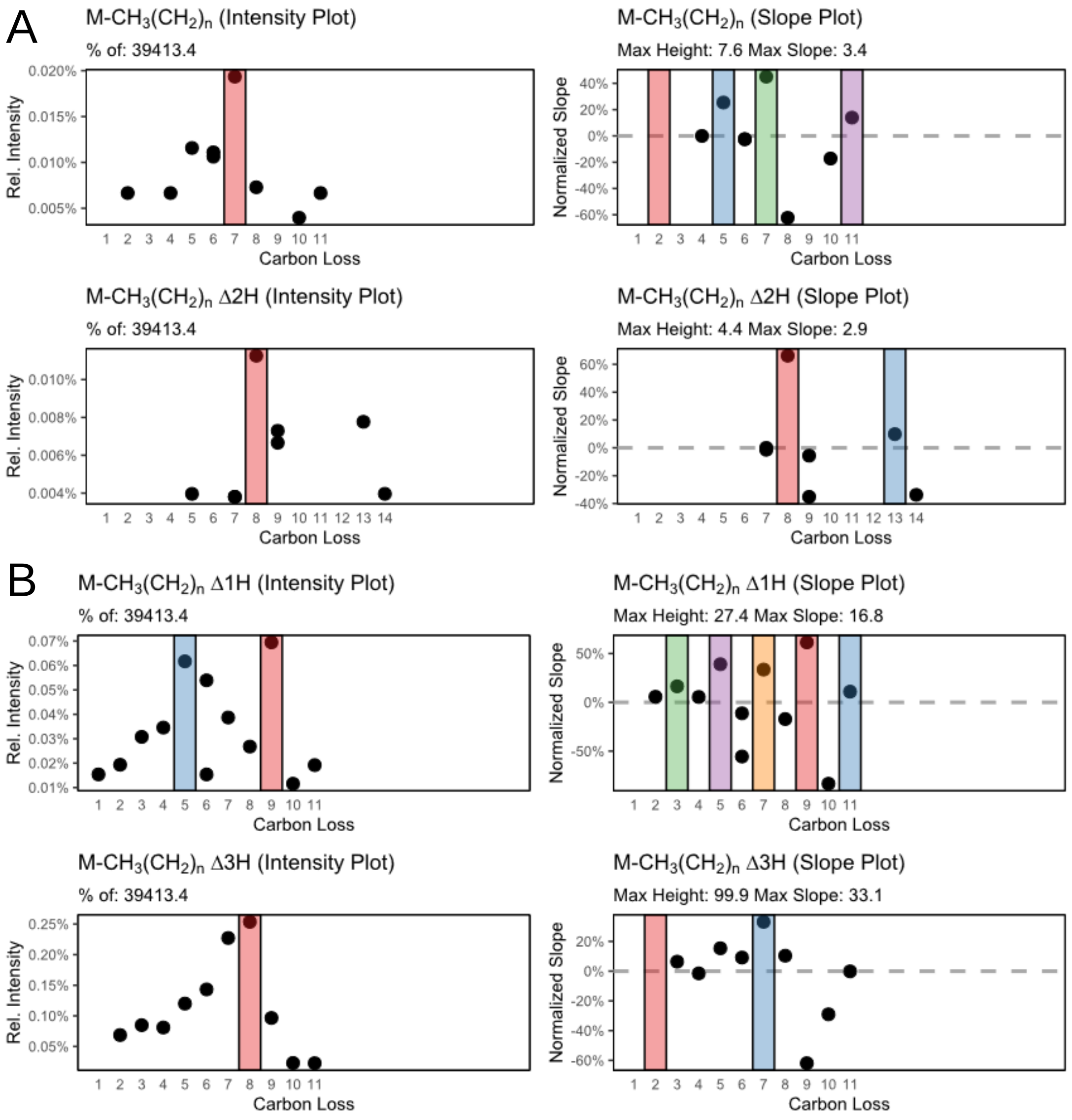

Figure S12: 35 eV CID of SLS $[M\text{-}H]^+$ with extracted fragmentation series starting from A) M-FA+H-$CH_3(CH_2)_n$ (*m/z* 603.5346) and B) $[L\text{-}H_2O]^+$ (*m/z* 259.2068). Colored bars indicate intensity peak picking results from MsRadaR.

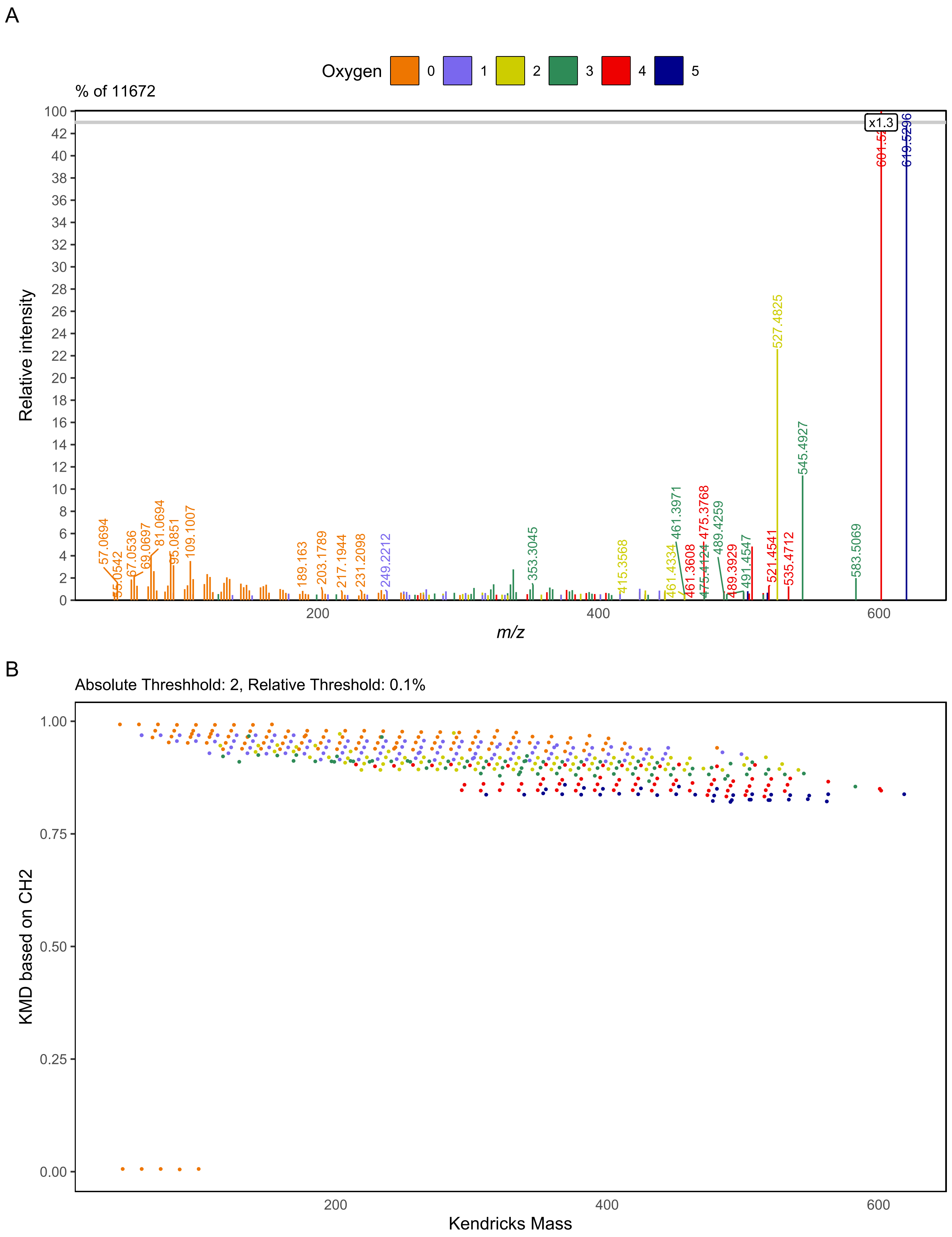

Figure S13: 35 eV CID of 1,3OO $[M-H]^+$ with A) CID spectra and B) Kendricks plot from MsRadaR.

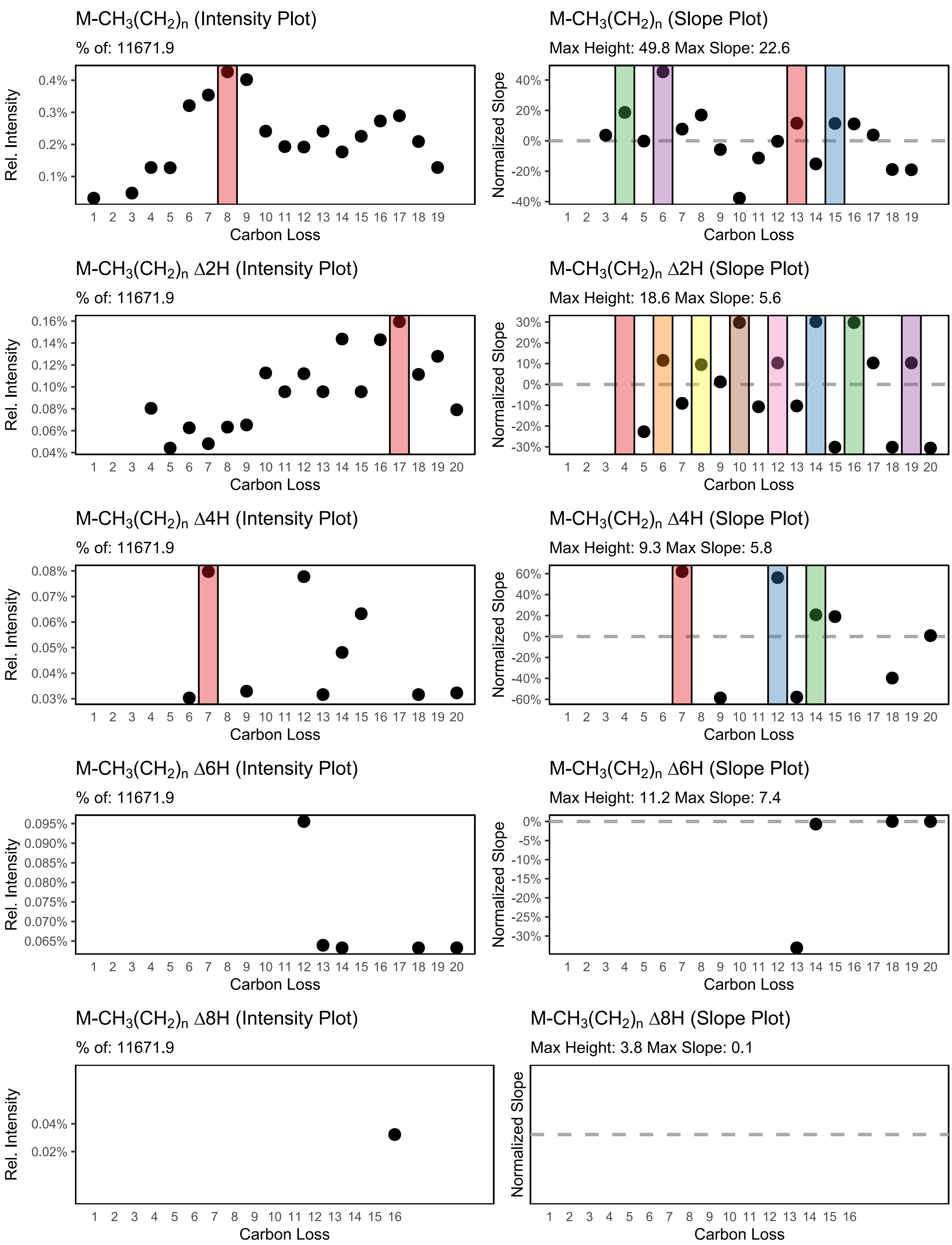

Figure S14: 35 eV CID of 1,3OO $[M-H]^+$ with extracted fragmentation series starting from the precursor. Colored bars indicate intensity peak picking results from MsRadaR.

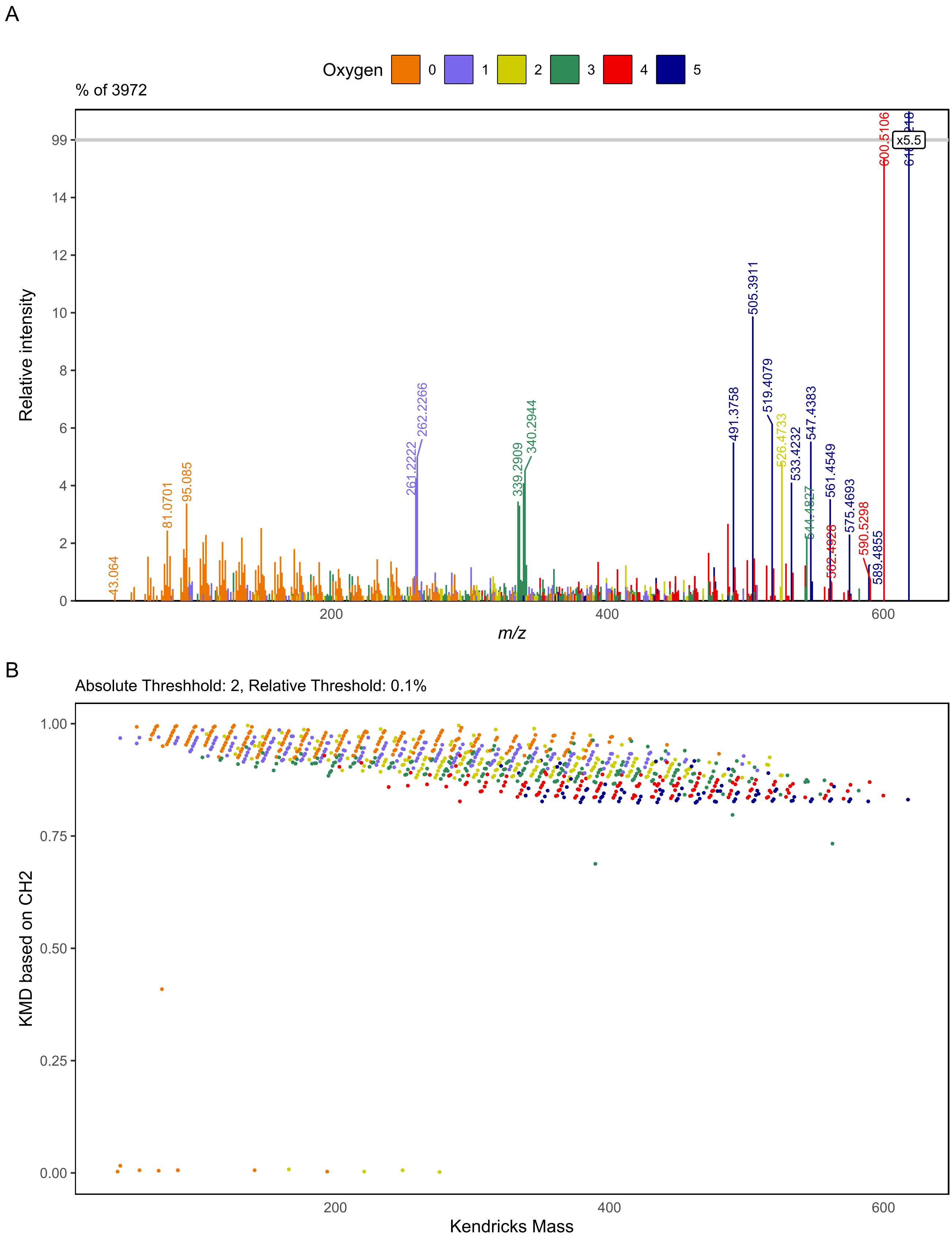

Figure S15: 35 eV CID of 1,2OL radical cation with A) CID spectra and B) Kendricks plot from MsRadaR.

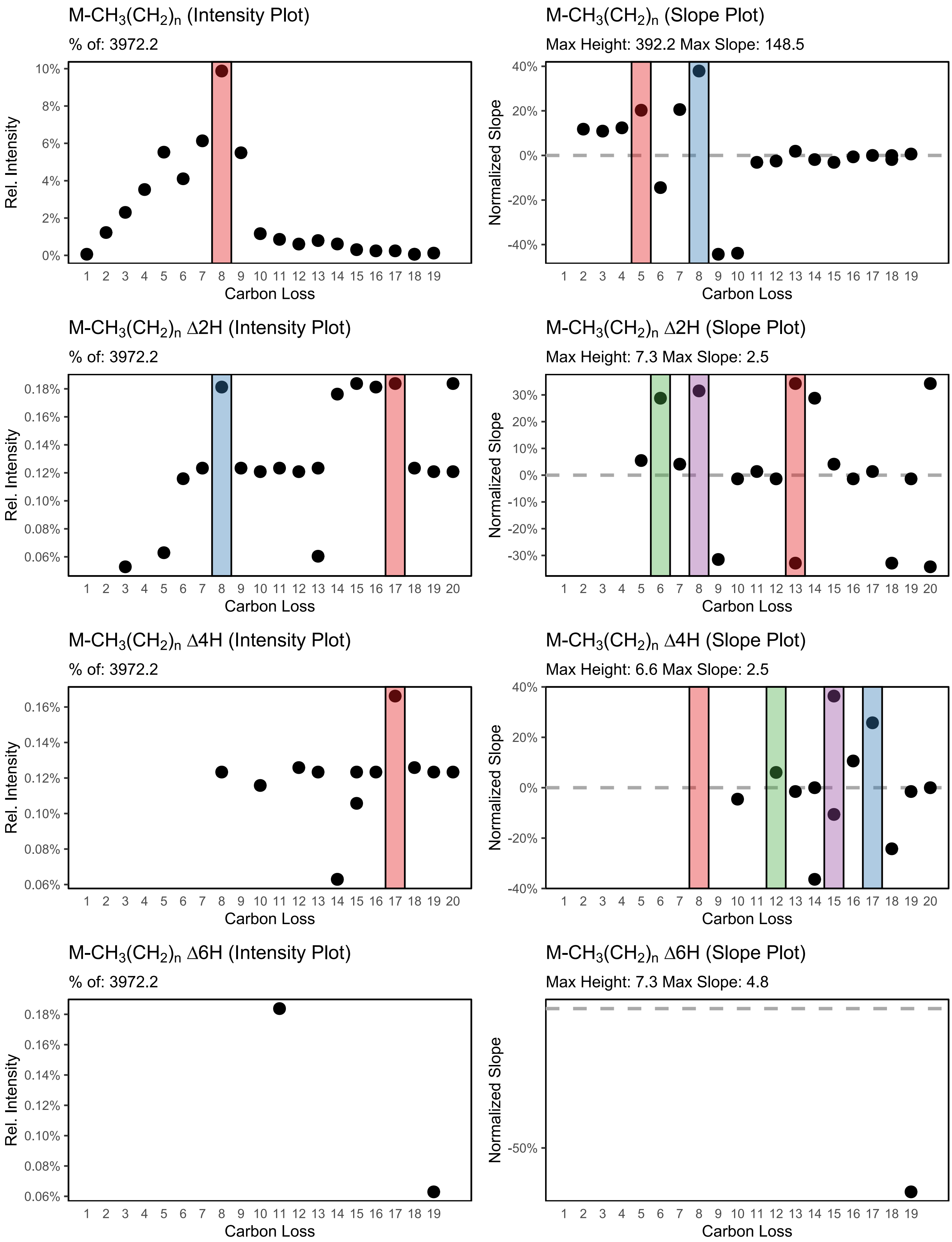

Figure S16: 35 eV CID of 1,2OL radical cation with extracted fragmentation series starting from the precursor. Colored bars indicate intensity peak picking results from MsRadaR.

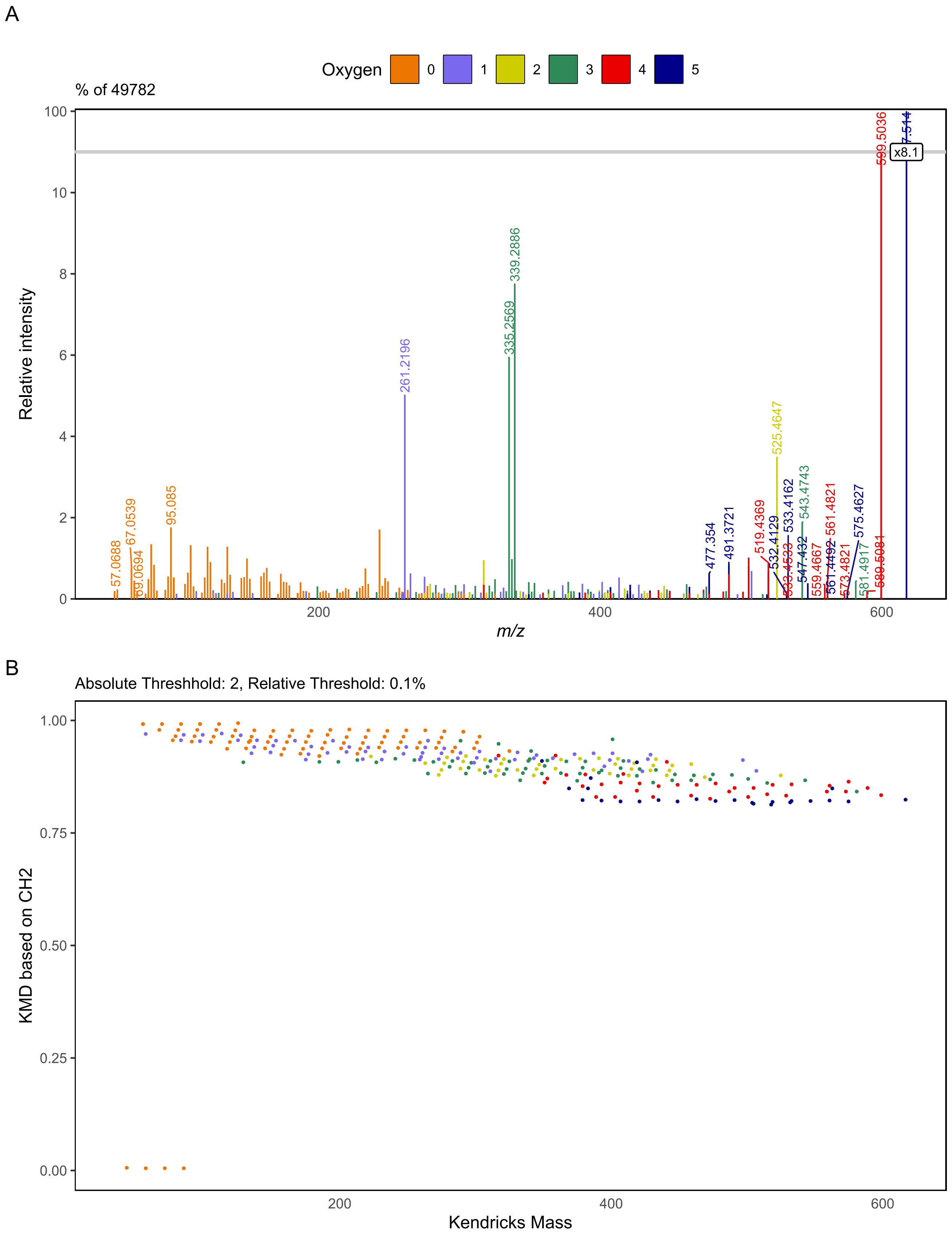

Figure S17: 35 eV CID of 1,2OL [M-H]$^+$ with A) CID spectra and B) Kendricks plot from MsRadaR.

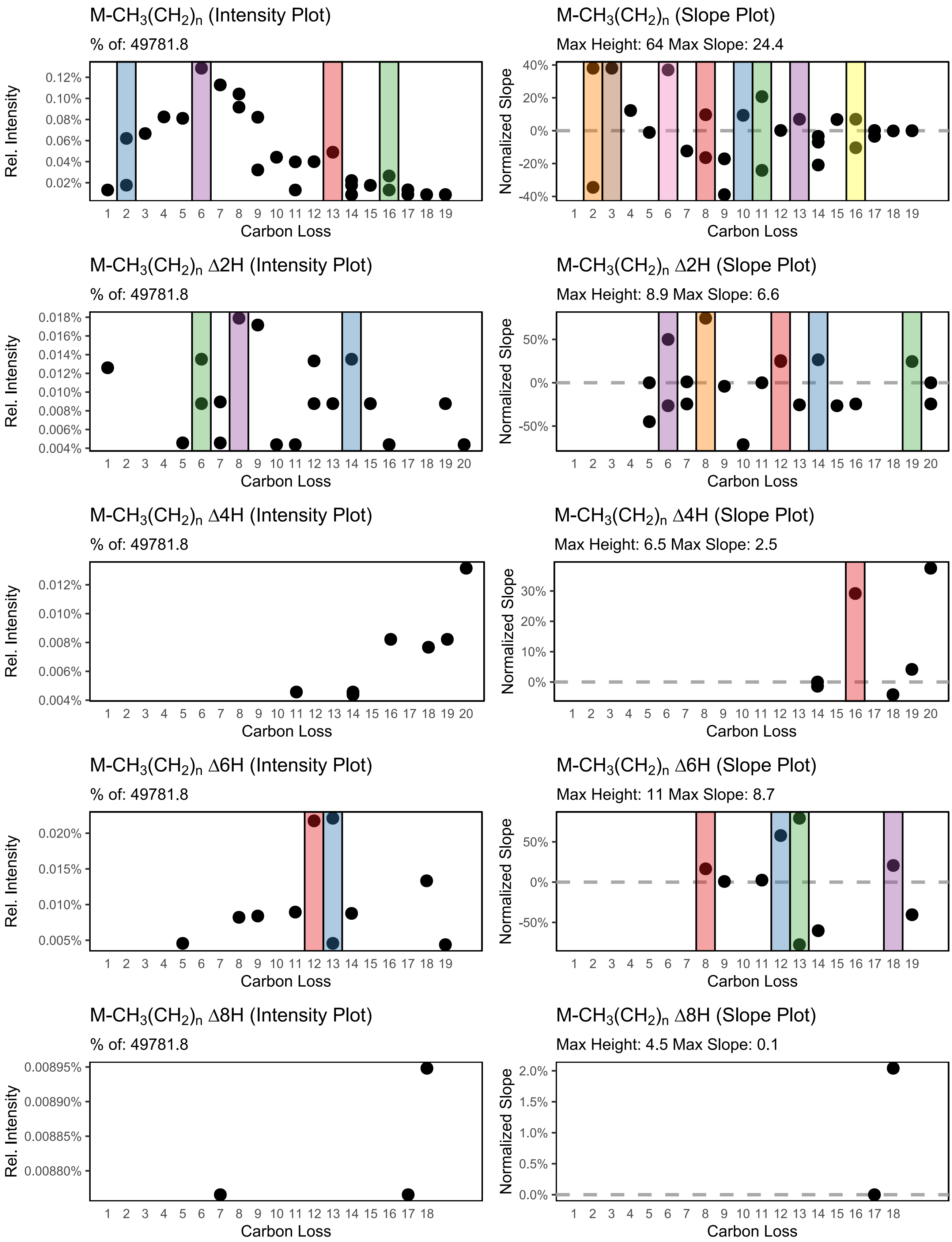

Figure S18: 35 eV CID of 1,2OL $[M-H]^+$ with extracted fragmentation series starting from the precursor. Colored bars indicate intensity peak picking results from MsRadaR.

A

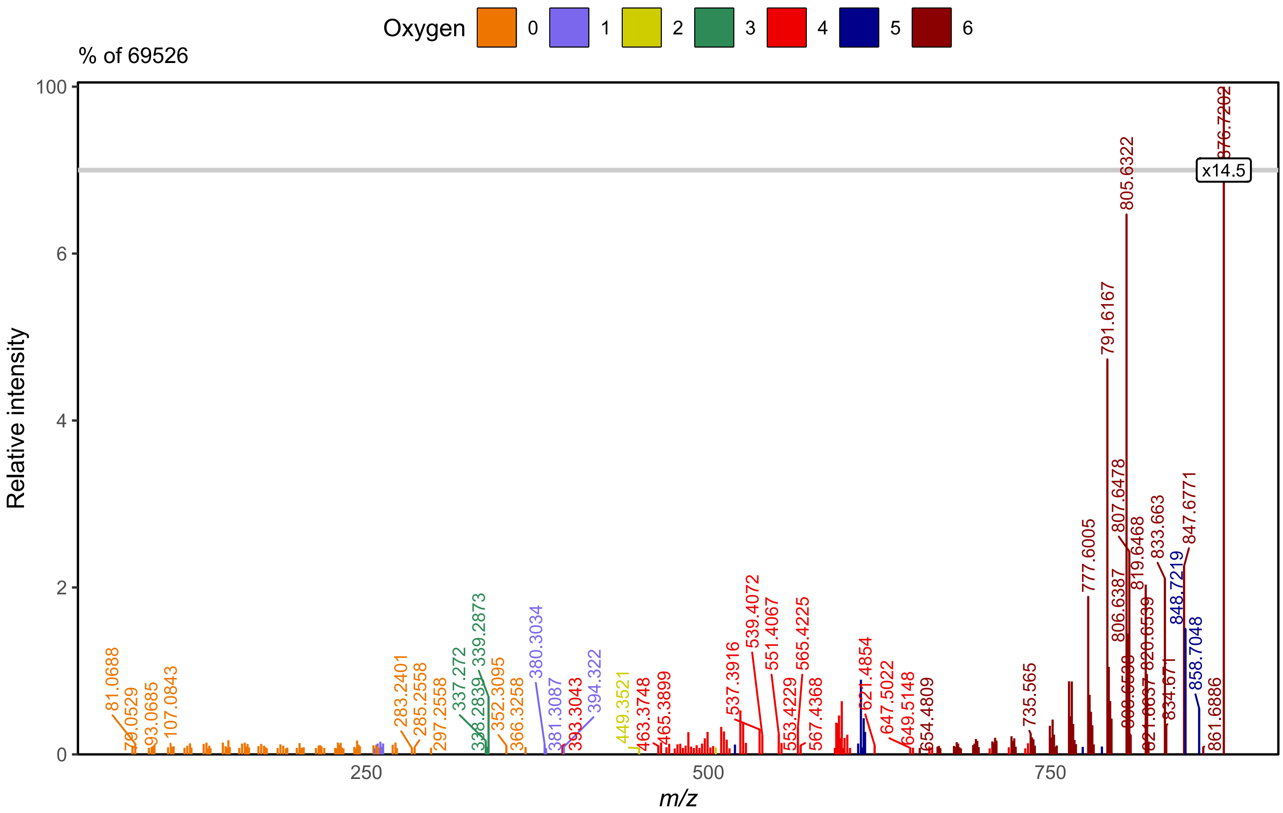

B

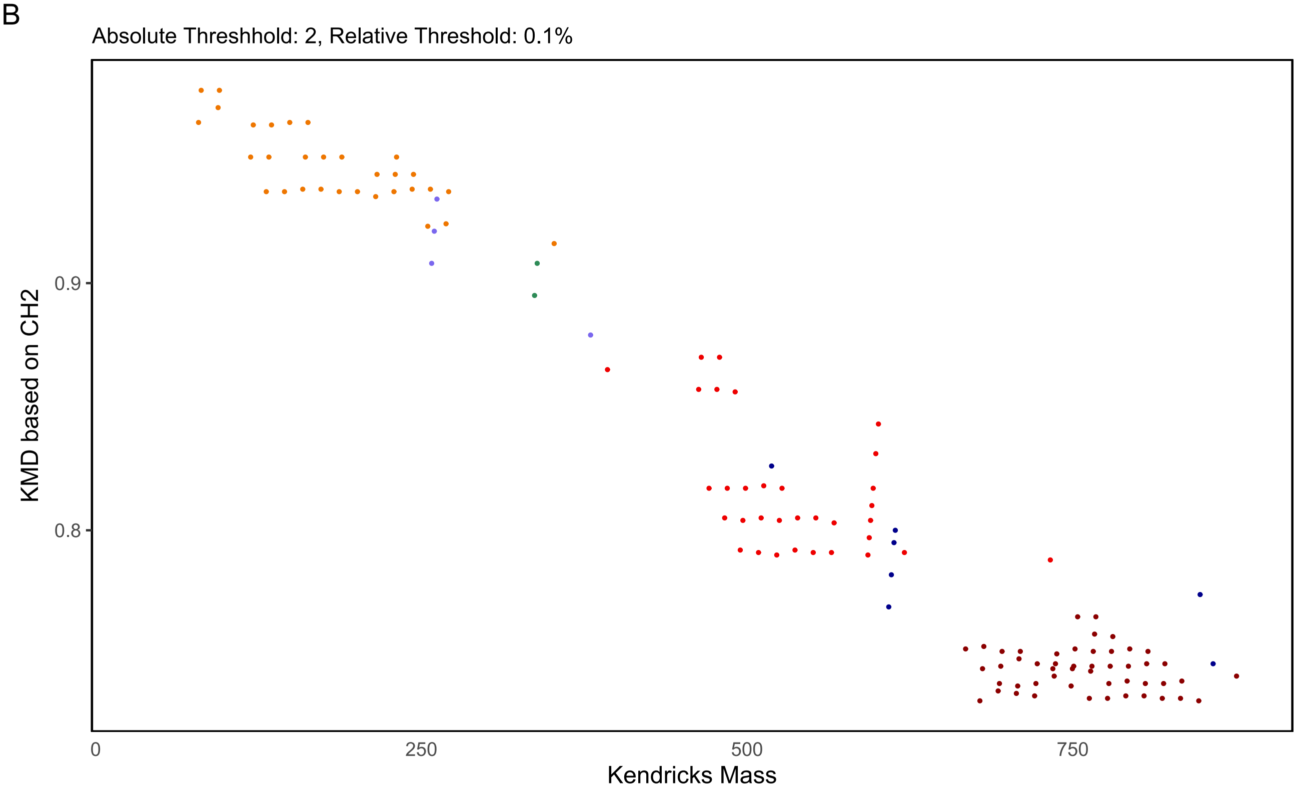

Figure S19: 35 eV CID of LLLn radical cation with A) CID spectra and B) Kendricks plot from MsRadaR.

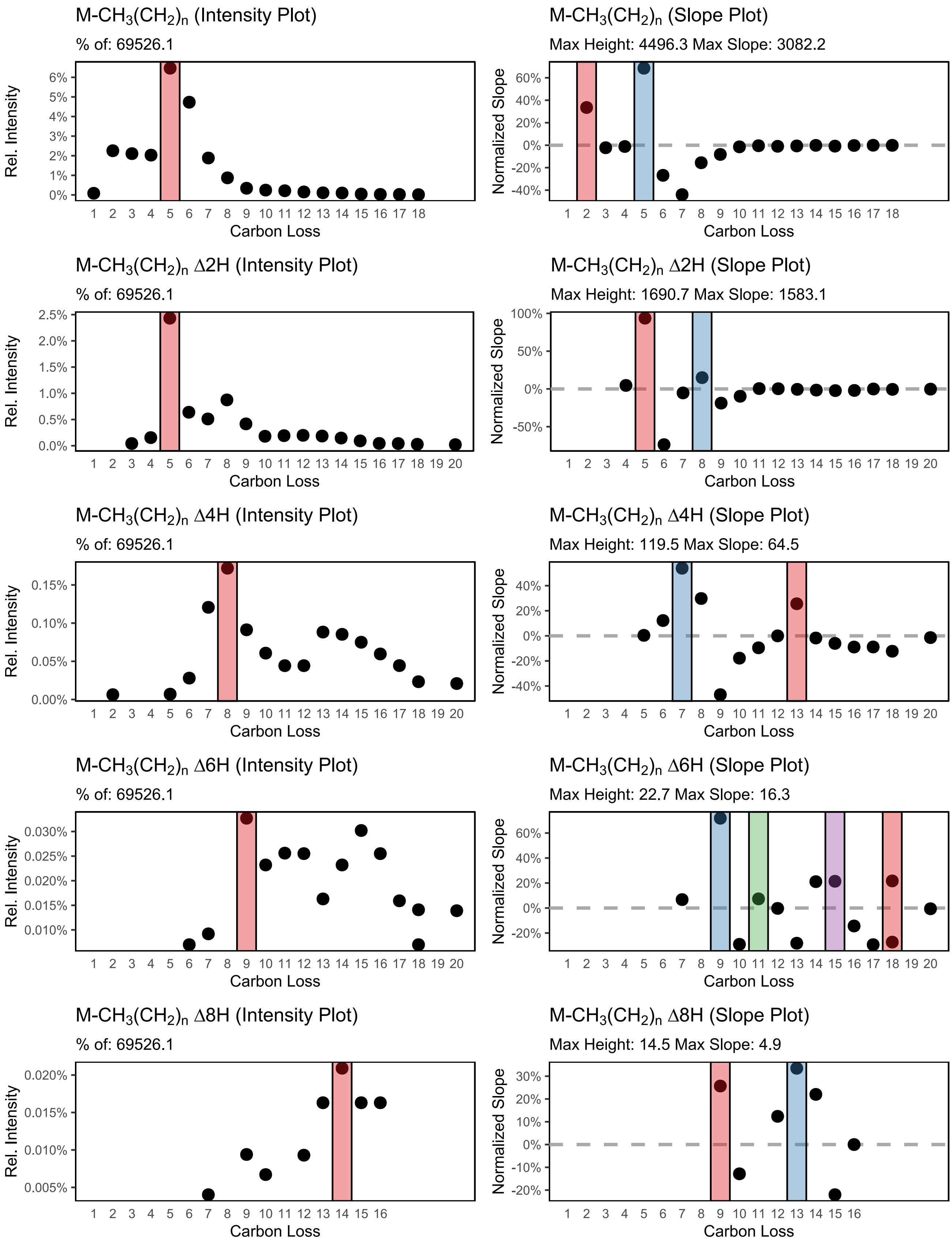

Figure S20: 35 eV CID of LLLn radical cation with extracted fragmentation series starting from the precursor. Colored bars indicate intensity peak picking results from MsRadaR.

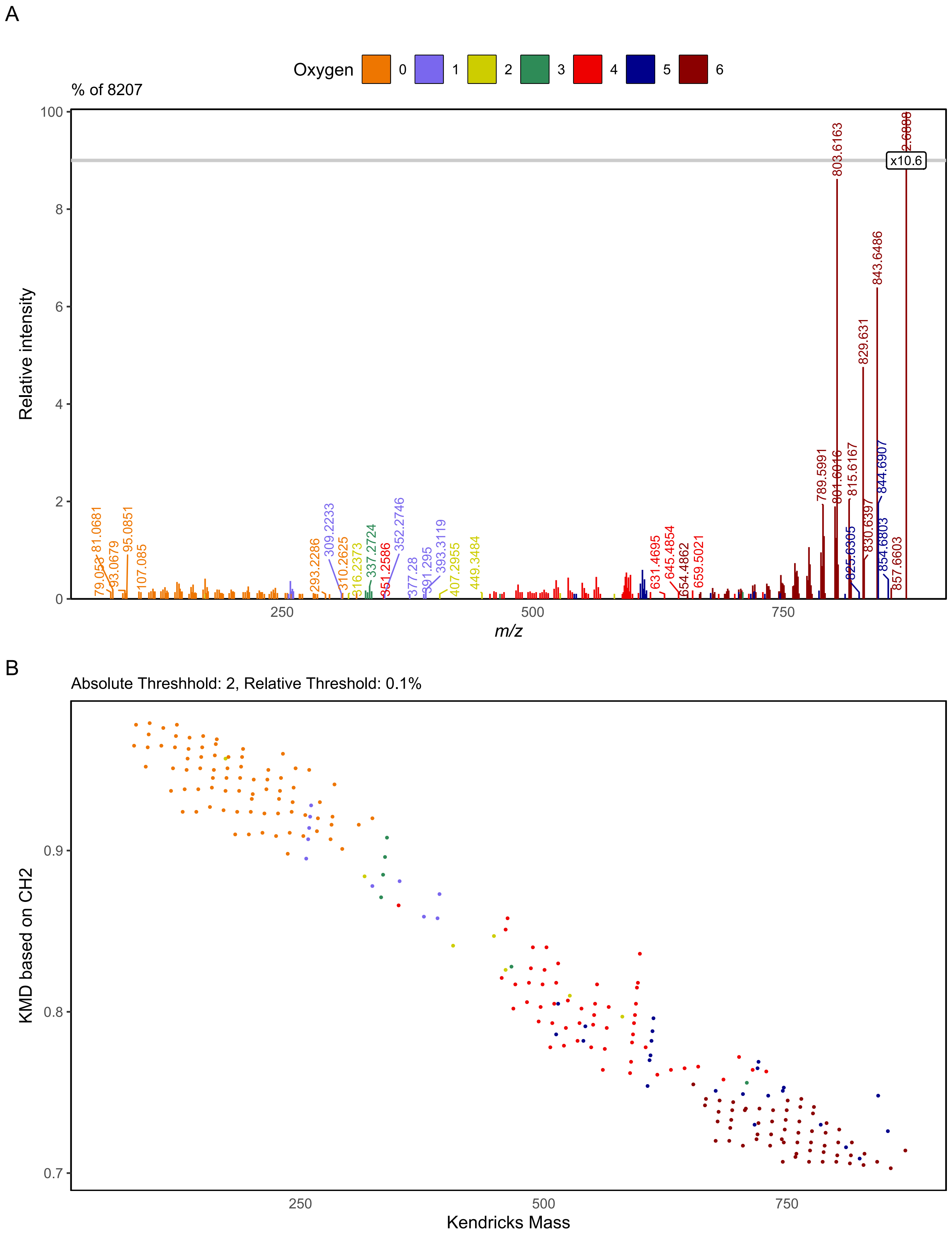

Figure S21: 35 eV CID of LnLnLn radical cation with A) CID spectra and B) Kendricks plot from MsRadaR.

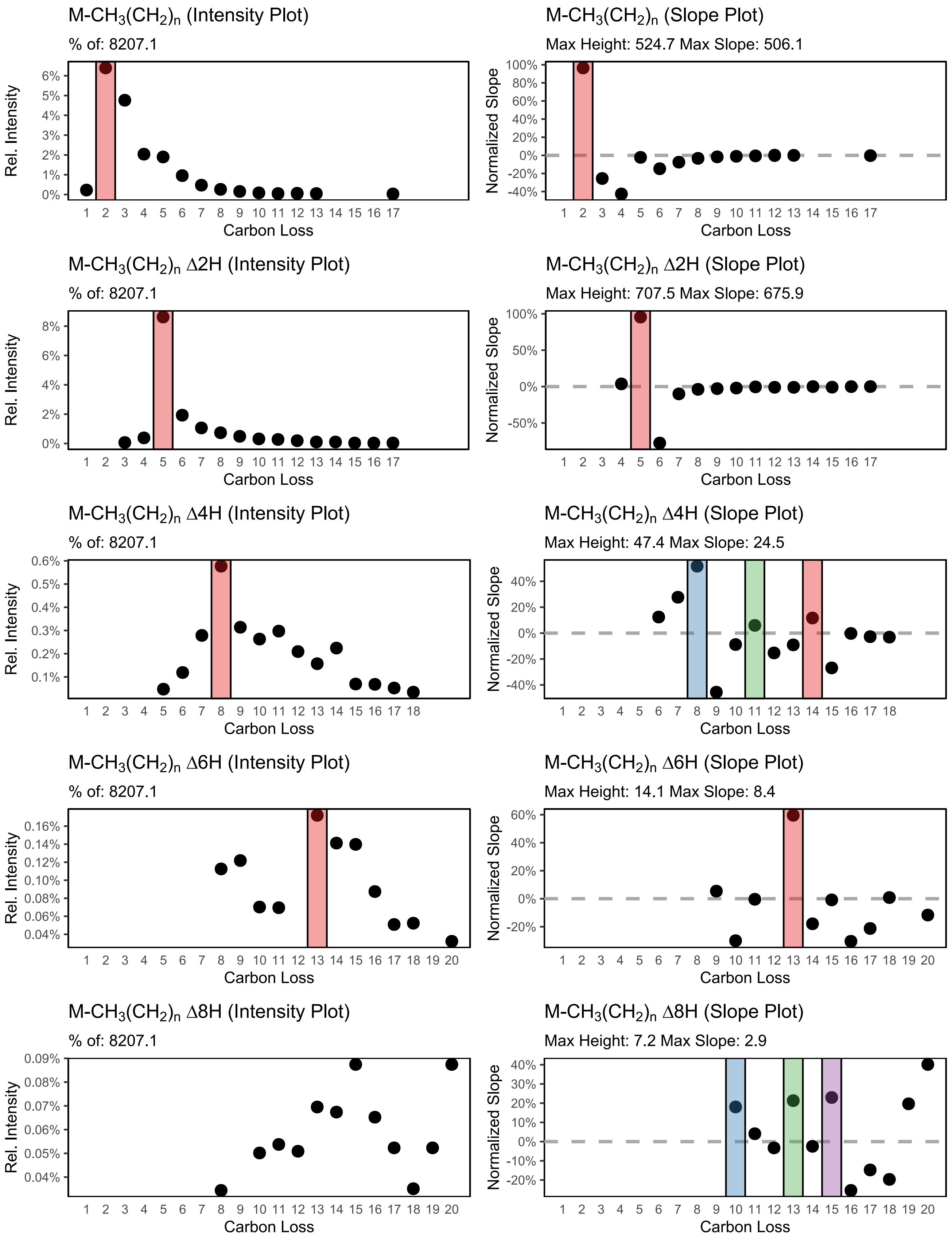

Figure S22: 35 eV CID of LnLnLn radical cation with extracted fragmentation series starting from the precursor. Colored bars indicate intensity peak picking results from MsRadaR.

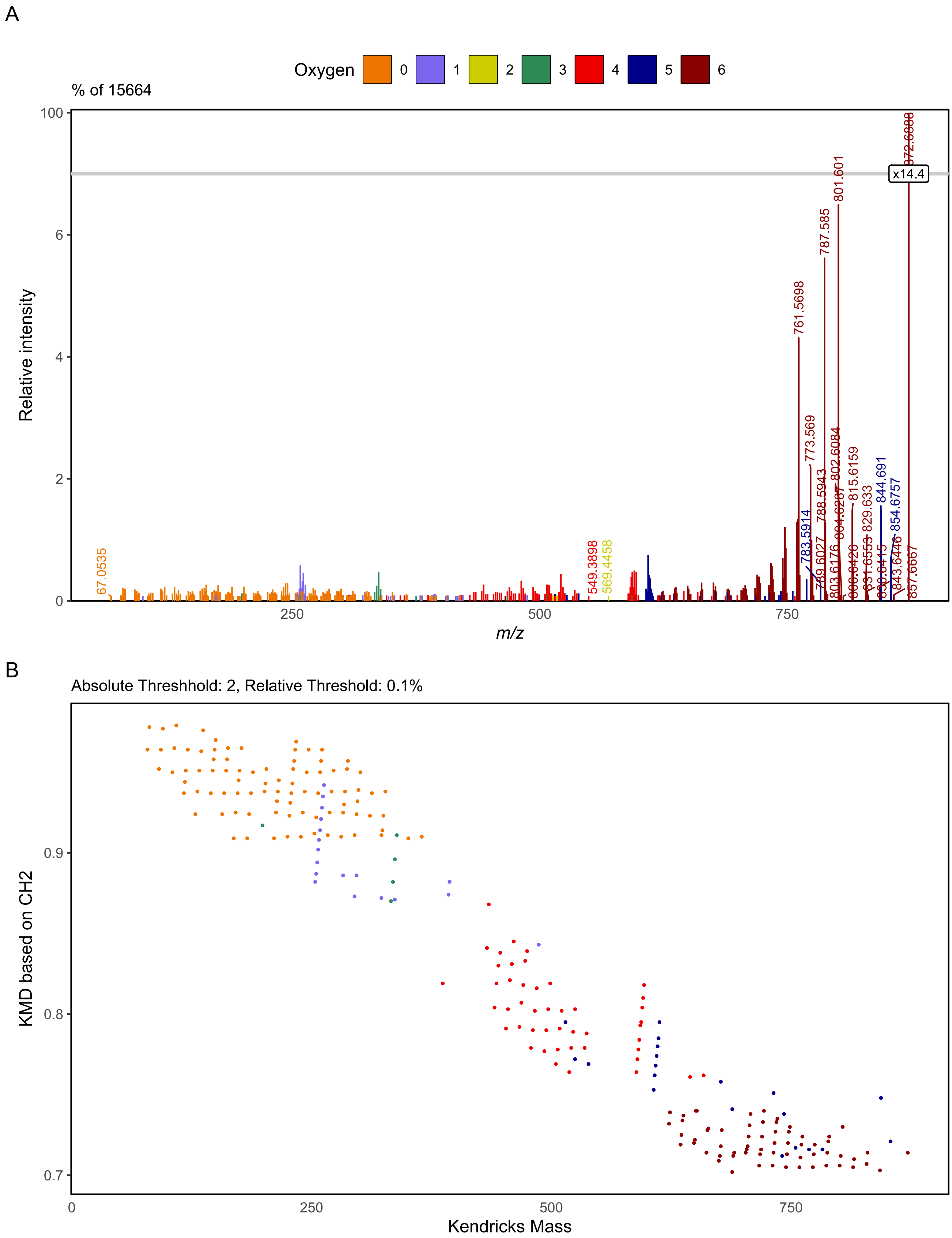

Figure S23: 35 eV CID of gLngLngLn radical cation with A) CID spectra and B) Kendricks plot from MsRadaR.

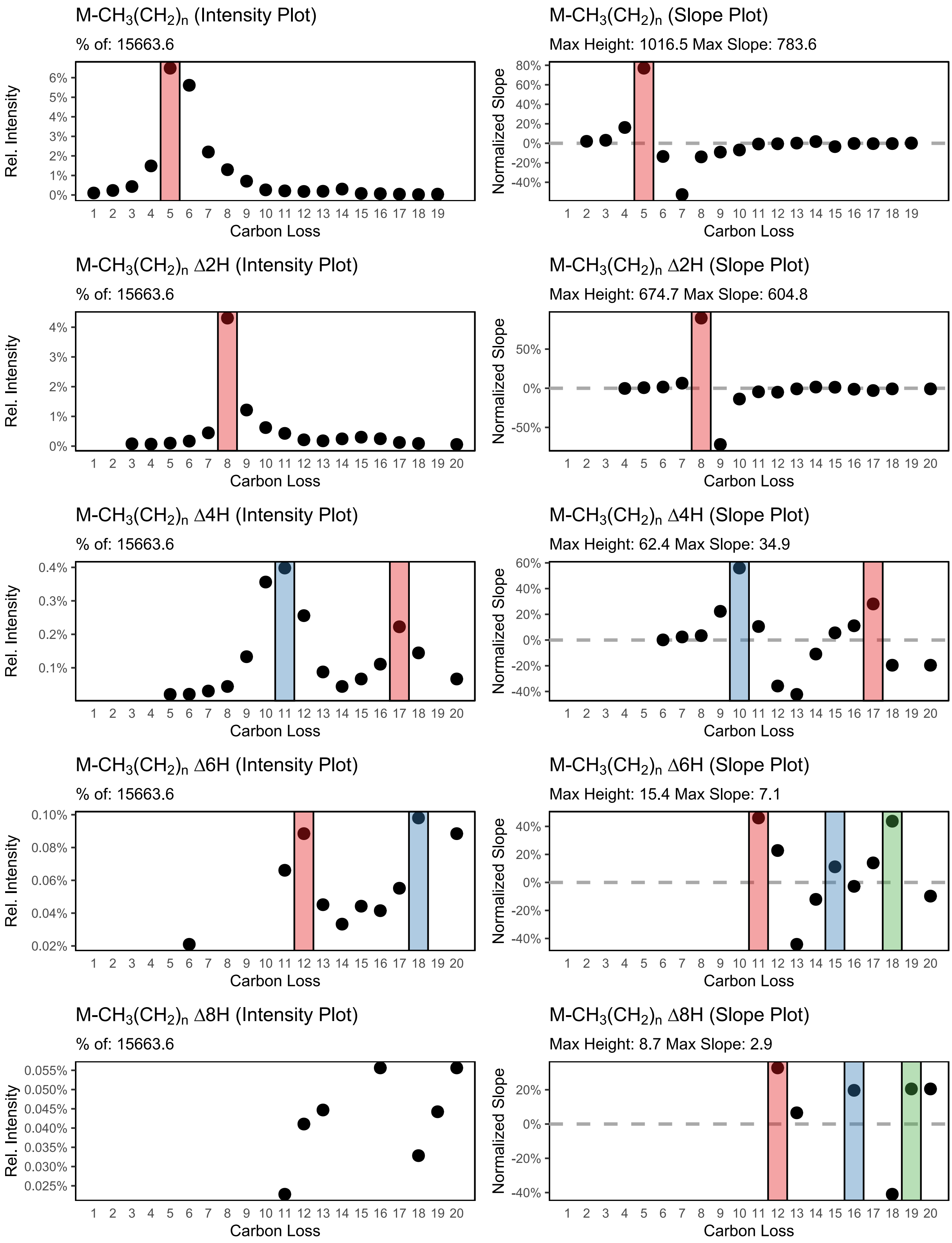

Figure S24: 35 eV CID of gLngLngLn radical cation with extracted fragmentation series starting from the precursor. Colored bars indicate intensity peak picking results from MsRadaR.

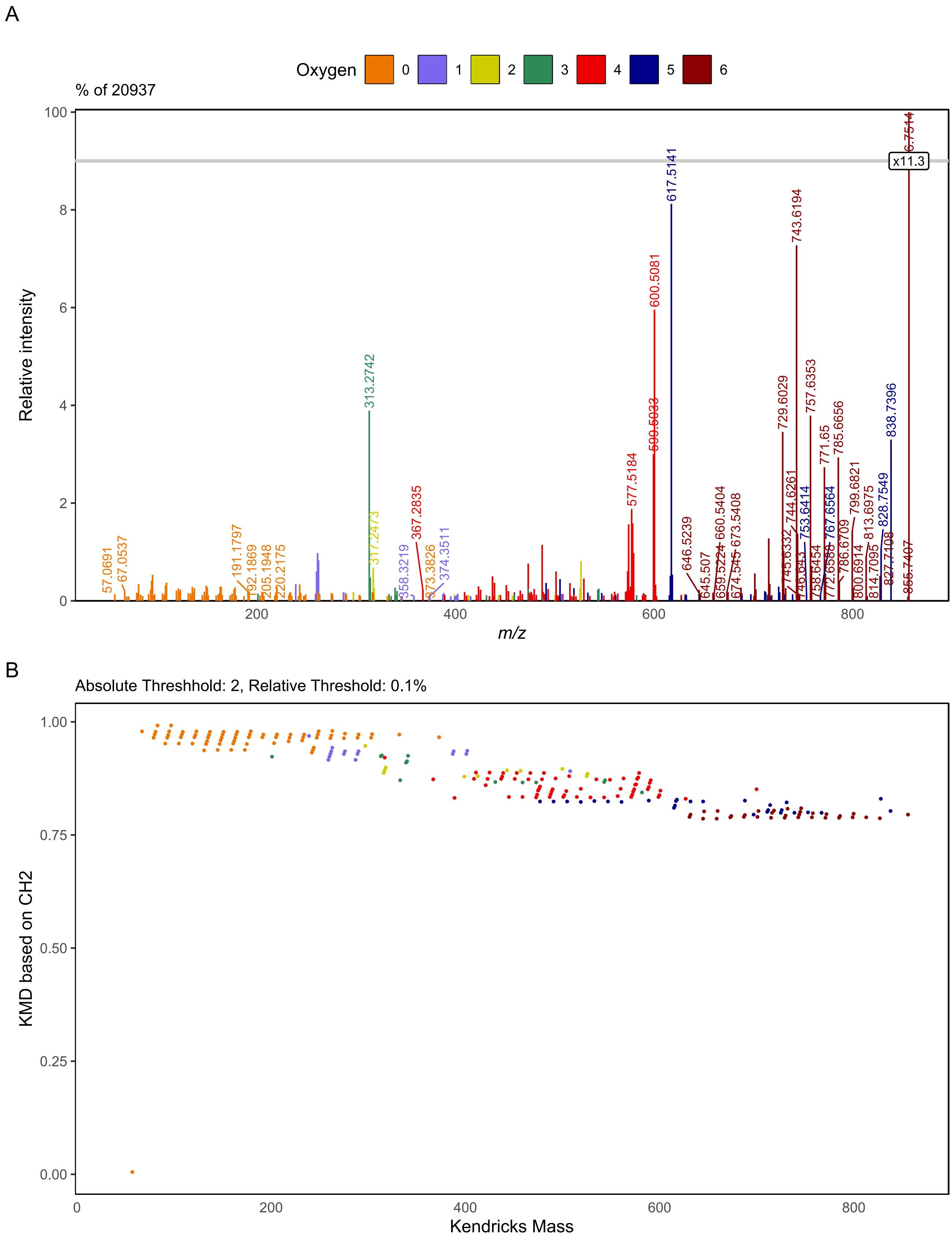

Figure S25: 35 eV CID of OPL radical cation with A) CID spectra and B) Kendricks plot from MsRadaR.

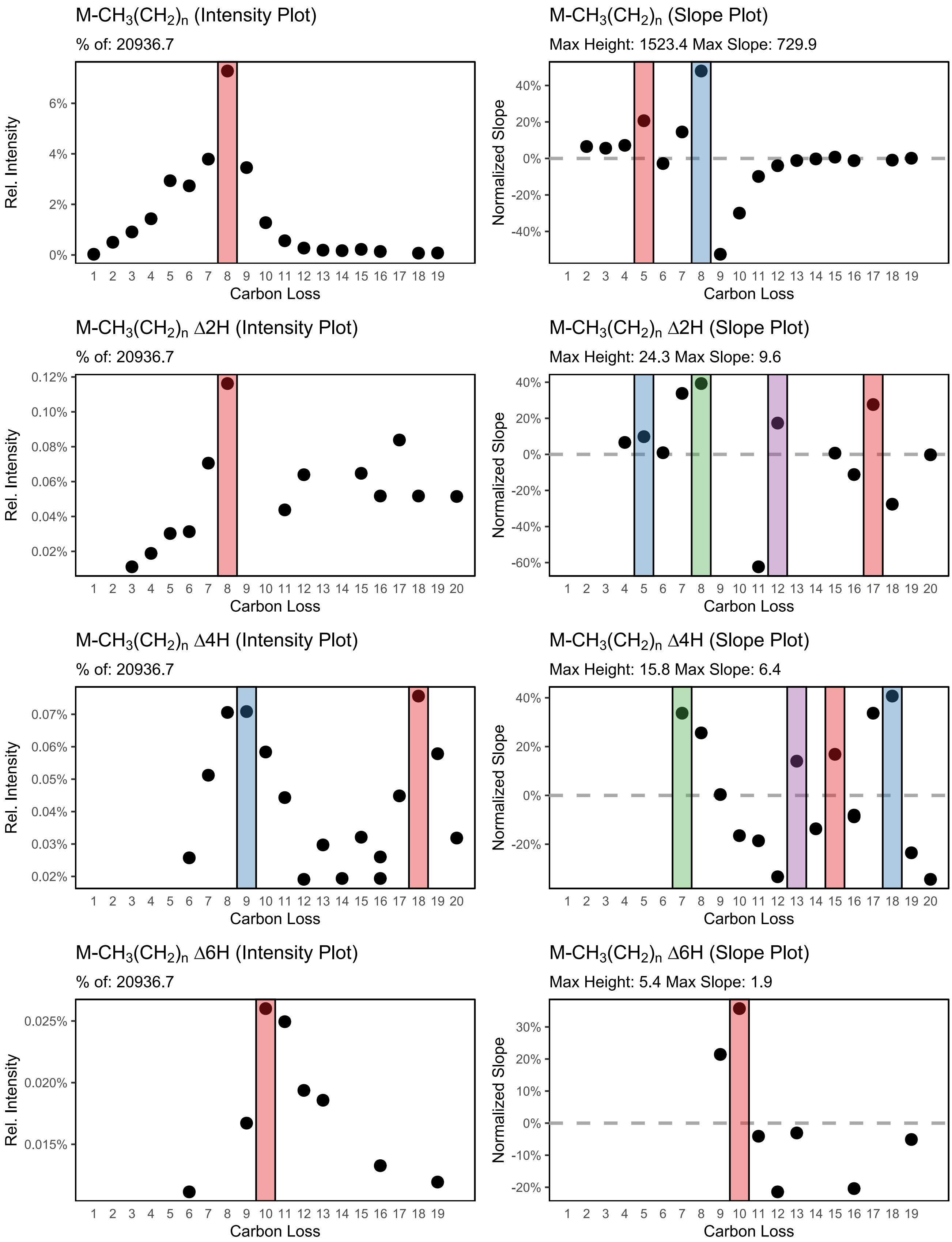

Figure S26: 35 eV CID of OPL radical cation with extracted fragmentation series starting from the precursor. Colored bars indicate intensity peak picking results from MsRadaR.

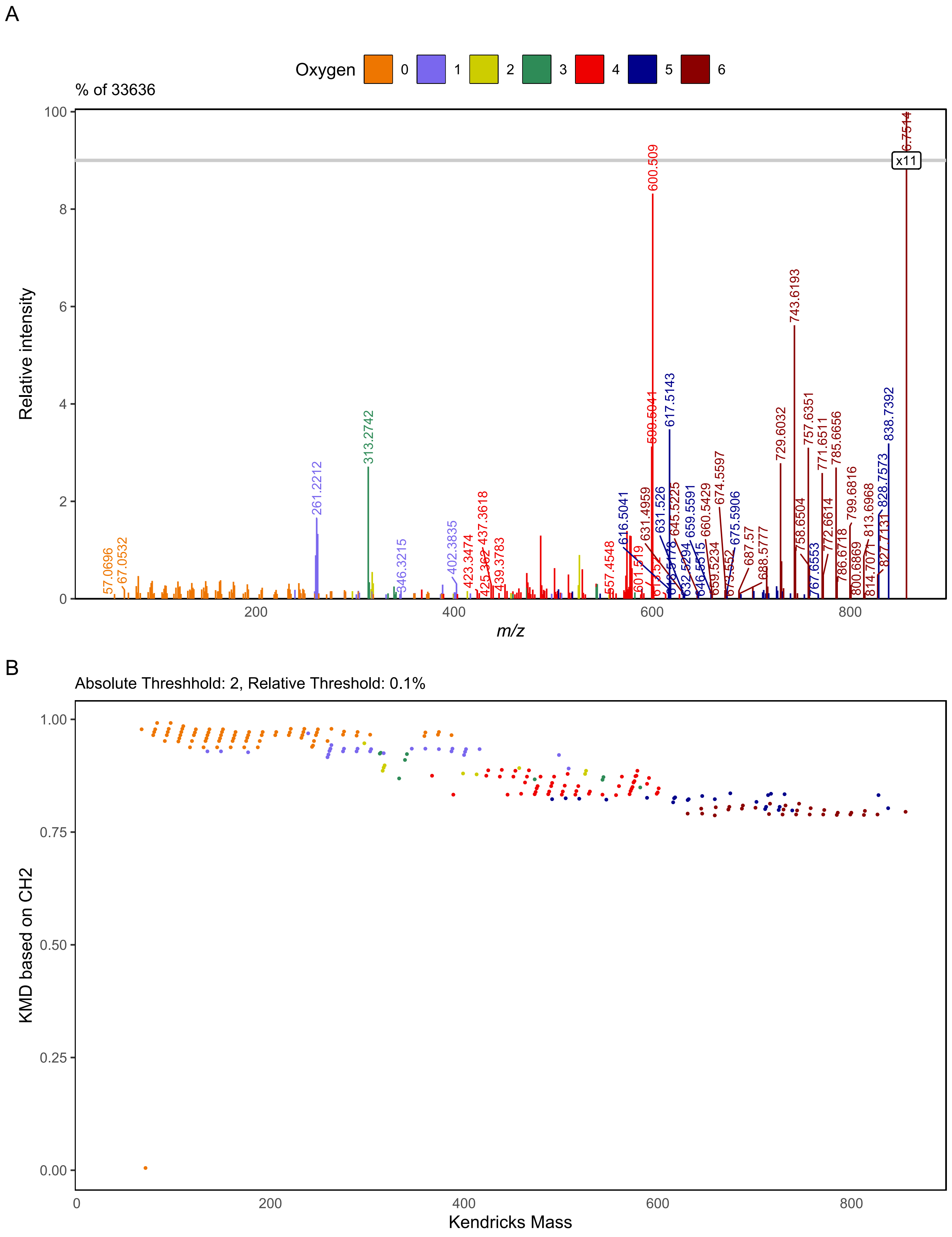

Figure S27: 35 eV CID of PLO radical cation with A) CID spectra and B) Kendricks plot from MsRadaR.

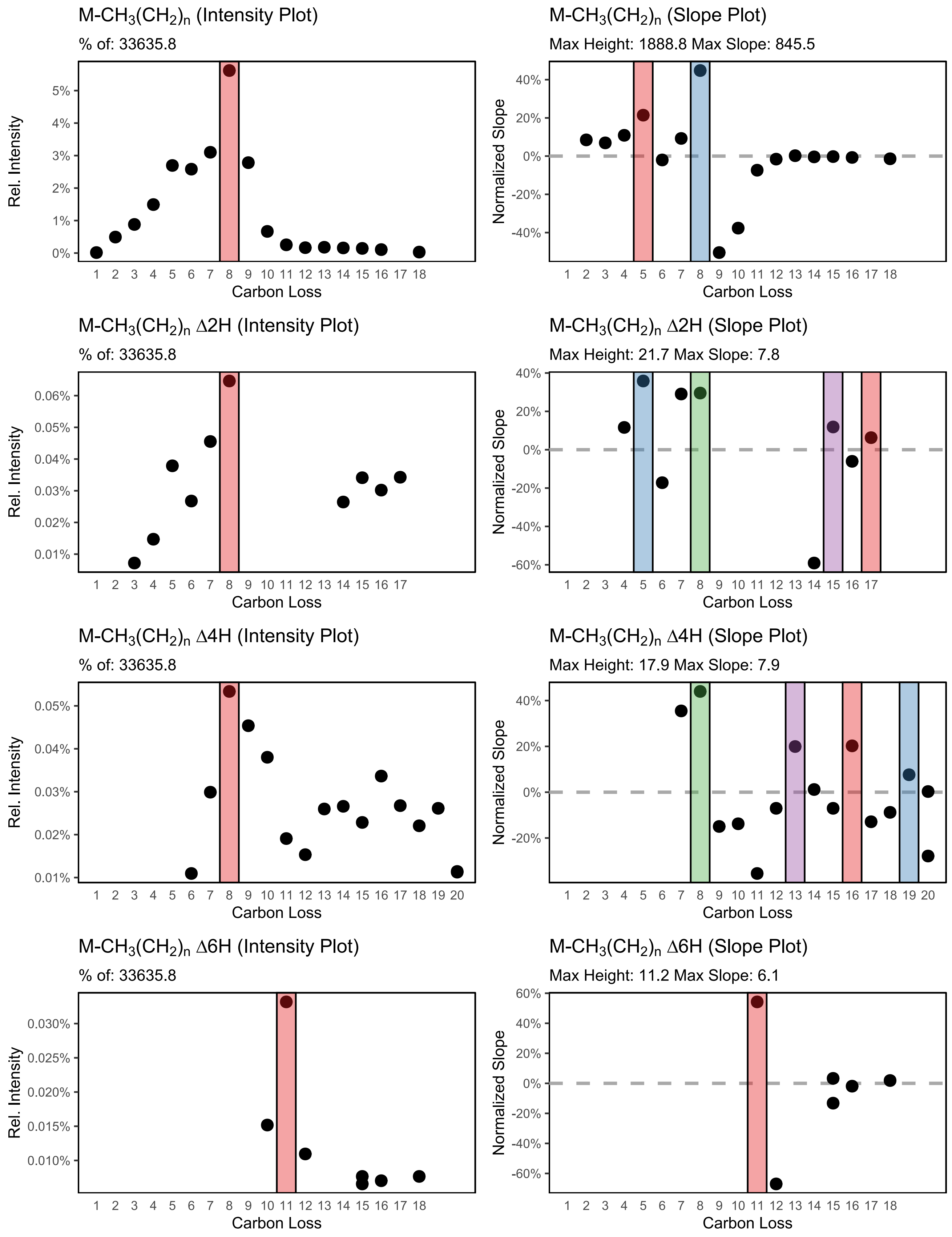

Figure S28: 35 eV CID of PLO radical cation with extracted fragmentation series starting from the precursor. Colored bars indicate intensity peak picking results from MsRadaR.

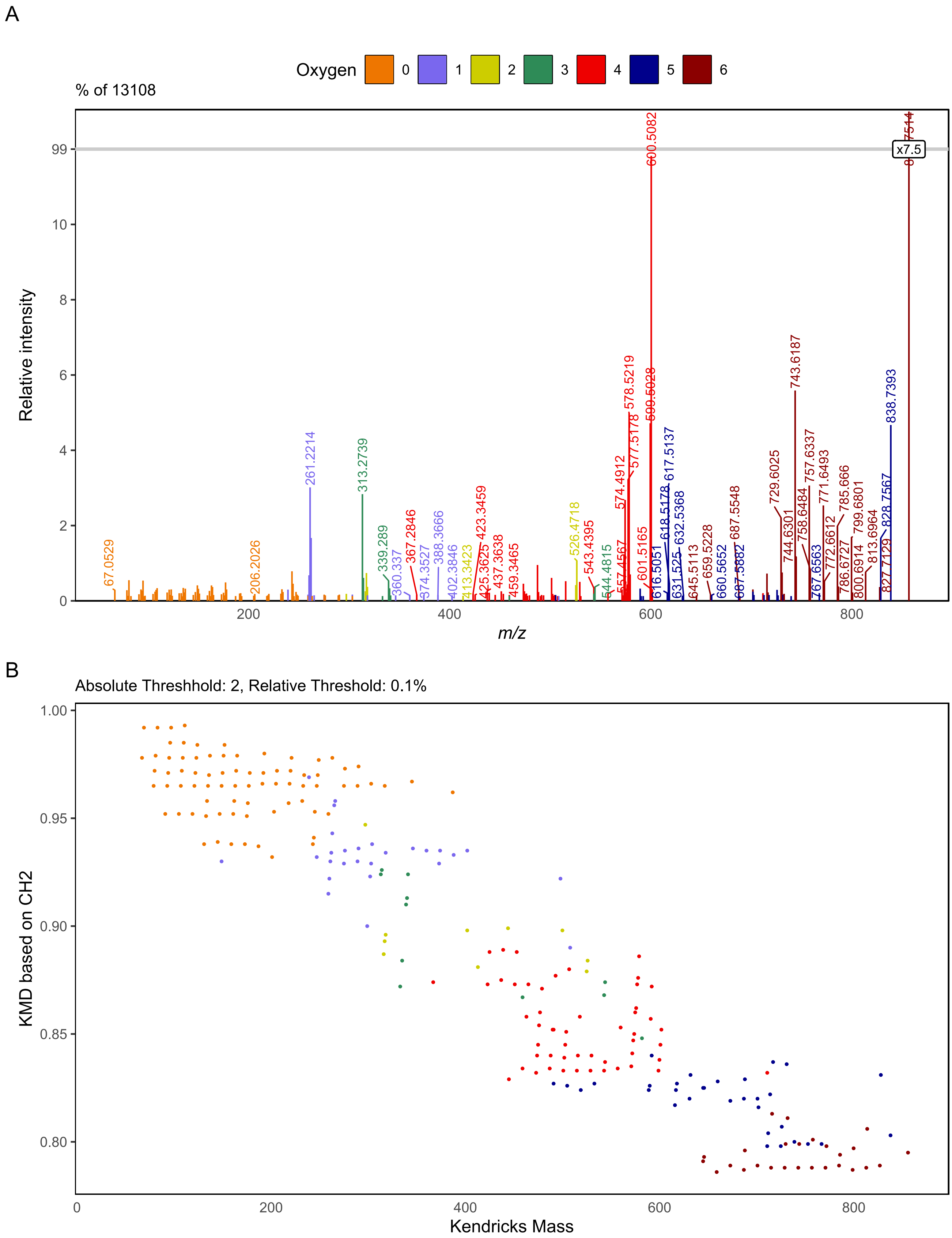

Figure S29: 35 eV CID of POL radical cation with A) CID spectra and B) Kendricks plot from MsRadaR.

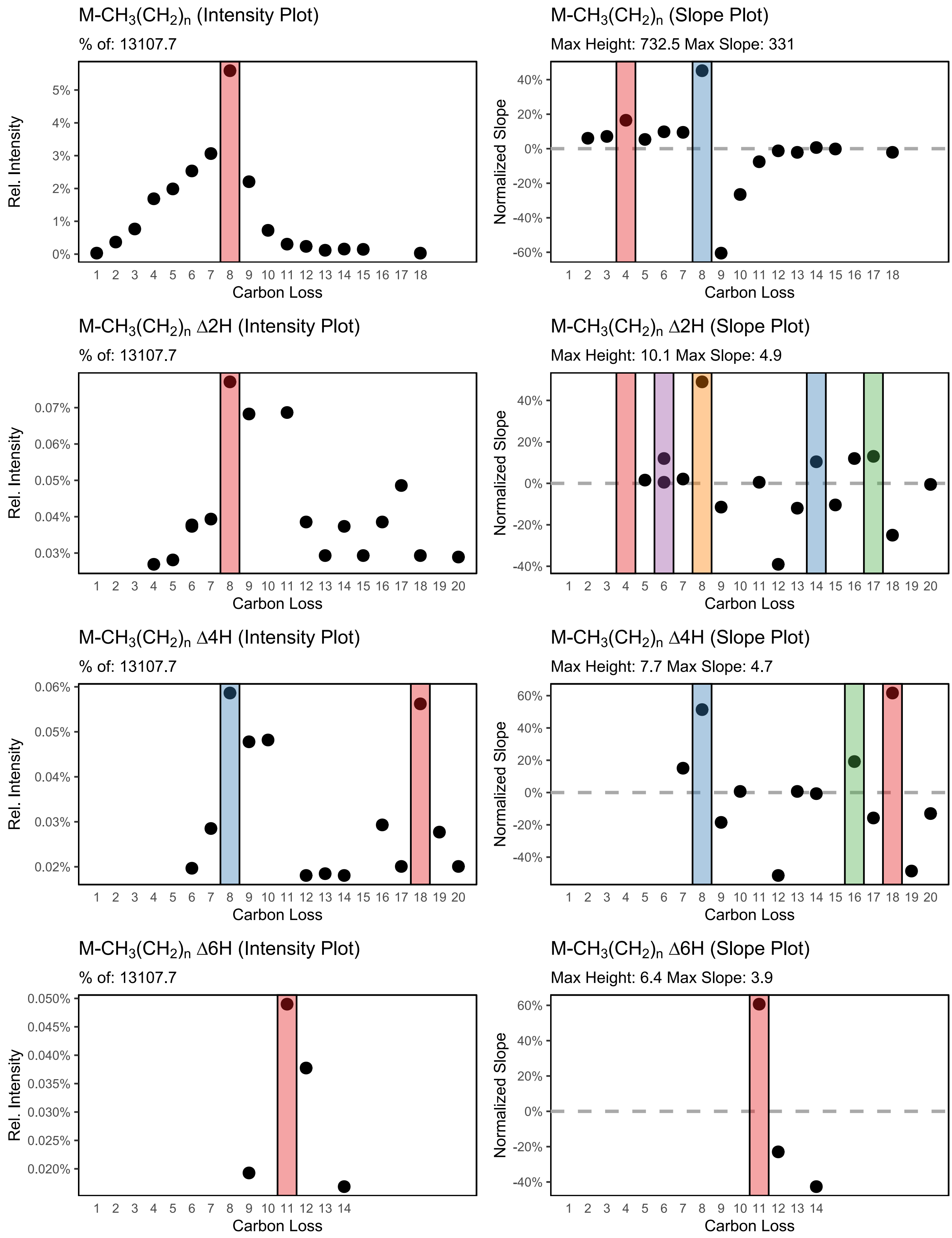

Figure S30: 35 eV CID of POL radical cation with extracted fragmentation series starting from the precursor. Colored bars indicate intensity peak picking results from MsRadaR.

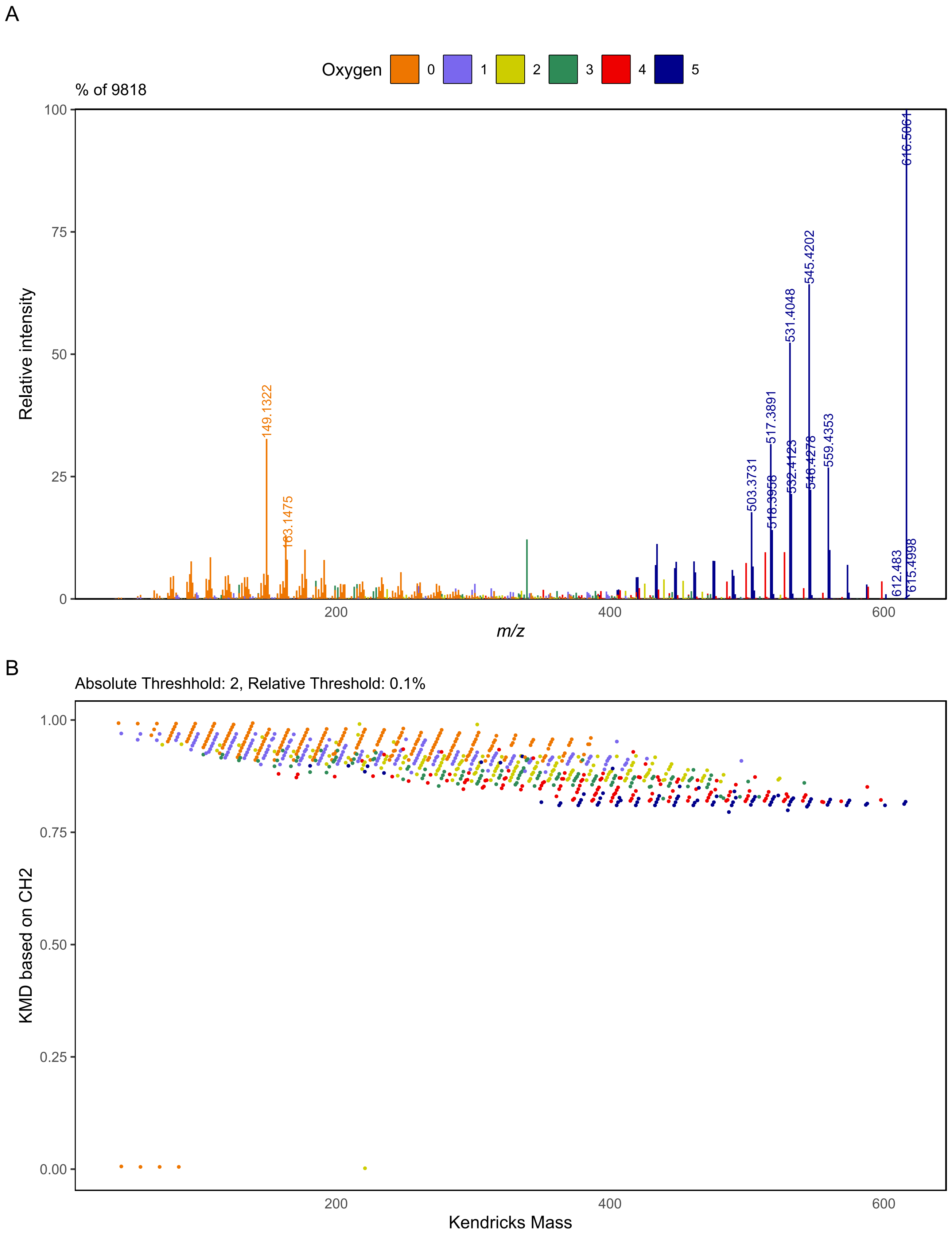

Figure S31: 35 eV CID of 1,3LL radical cation with A) CID spectra and B) Kendricks plot from MsRadaR.

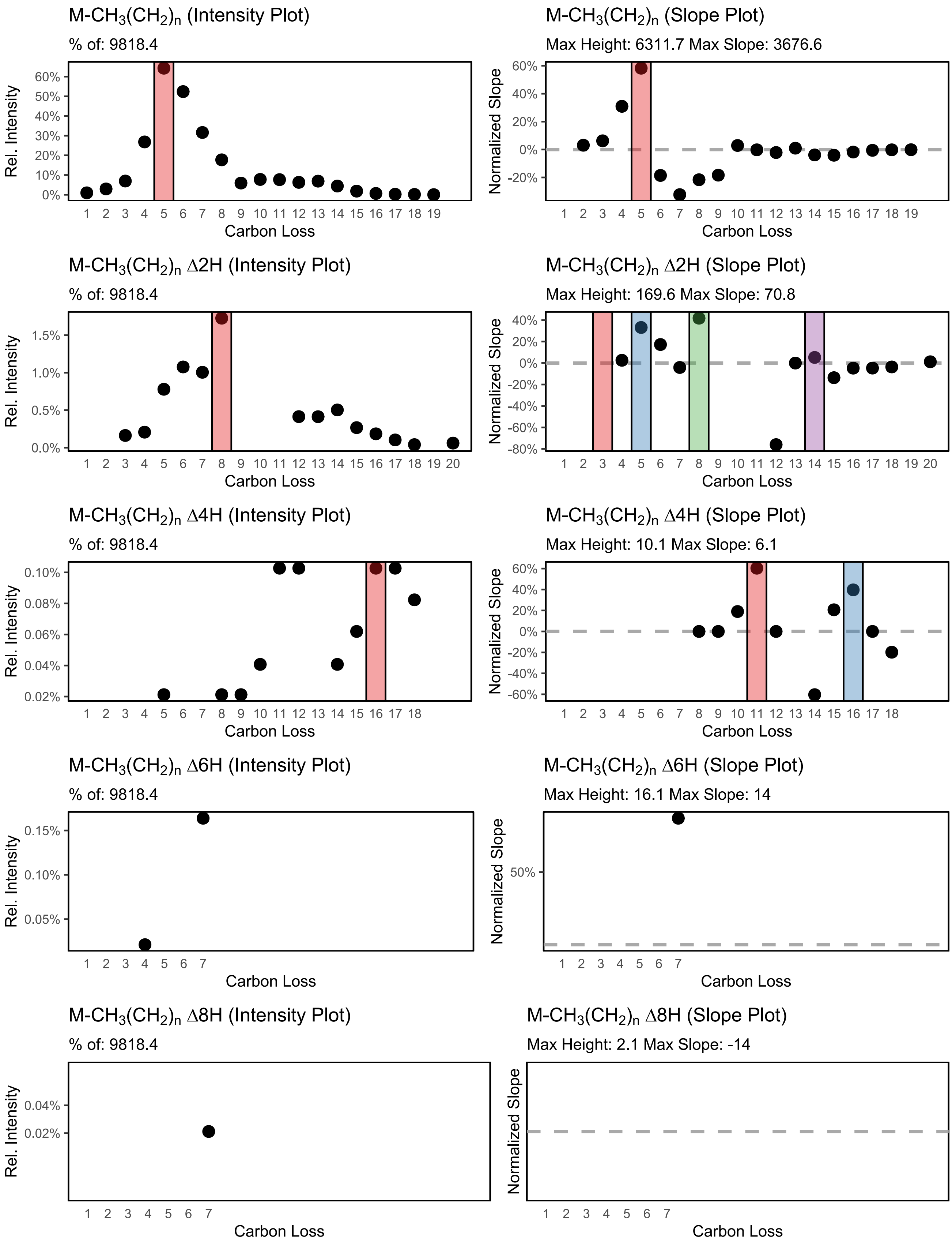

Figure S32: 35 eV CID of 1,3LL radical cation with extracted fragmentation series starting from the precursor. Colored bars indicate intensity peak picking results from MsRadaR.

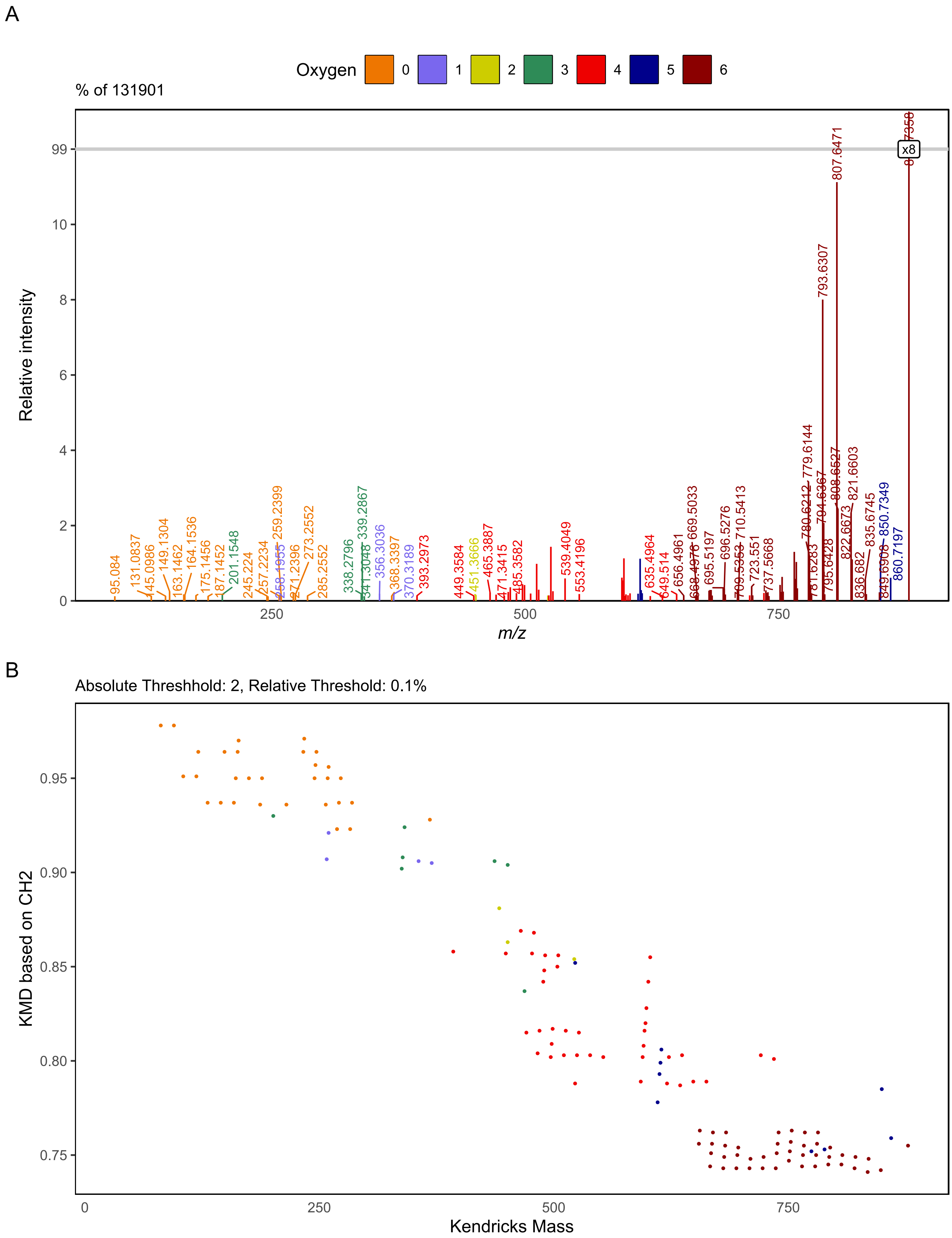

Figure S33: 35 eV CID of LLL radical cation with A) CID spectra and B) Kendricks plot from MsRadaR.

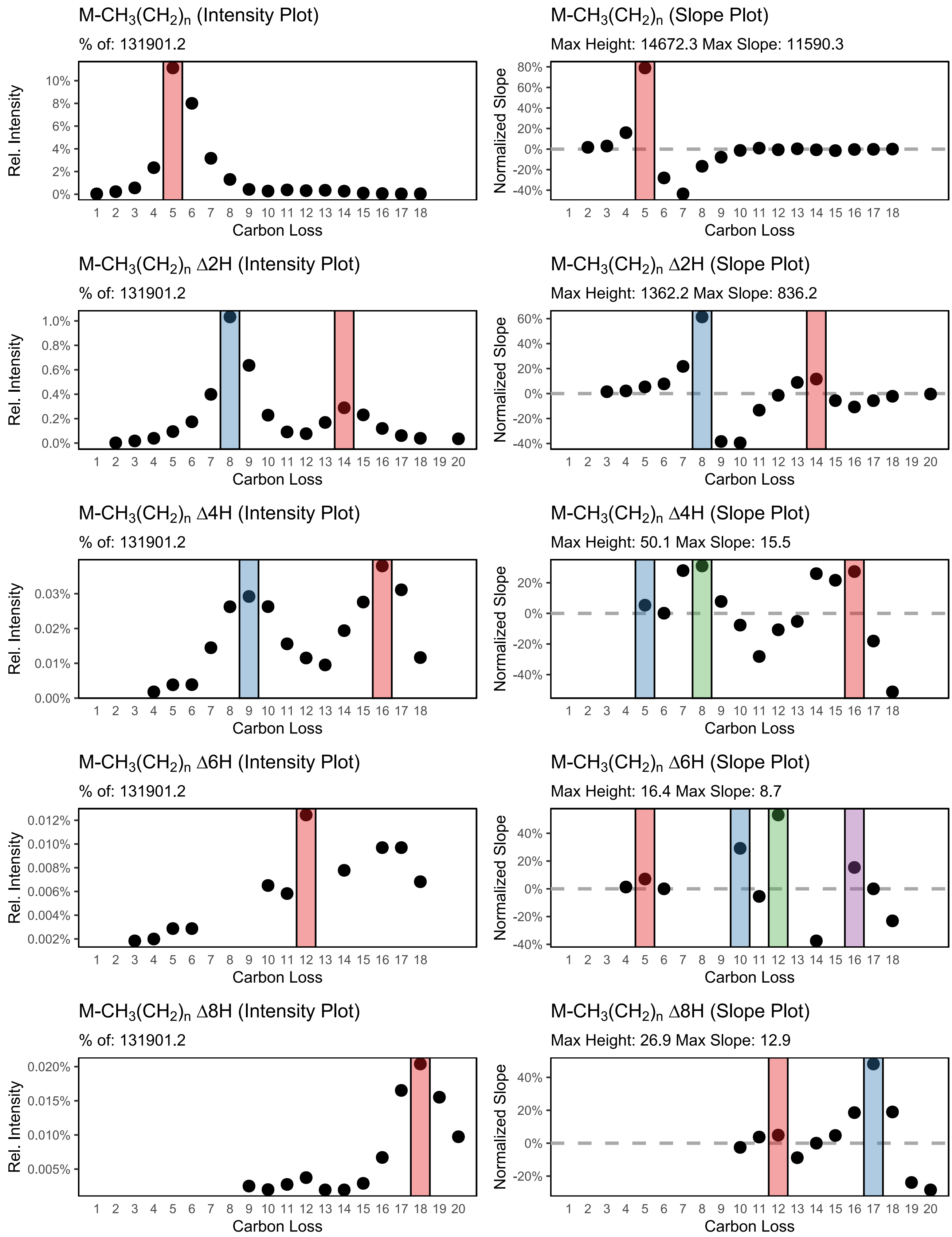

Figure S34: 35 eV CID of LLL radical cation with extracted fragmentation series starting from the precursor. Colored bars indicate intensity peak picking results from MsRadaR.

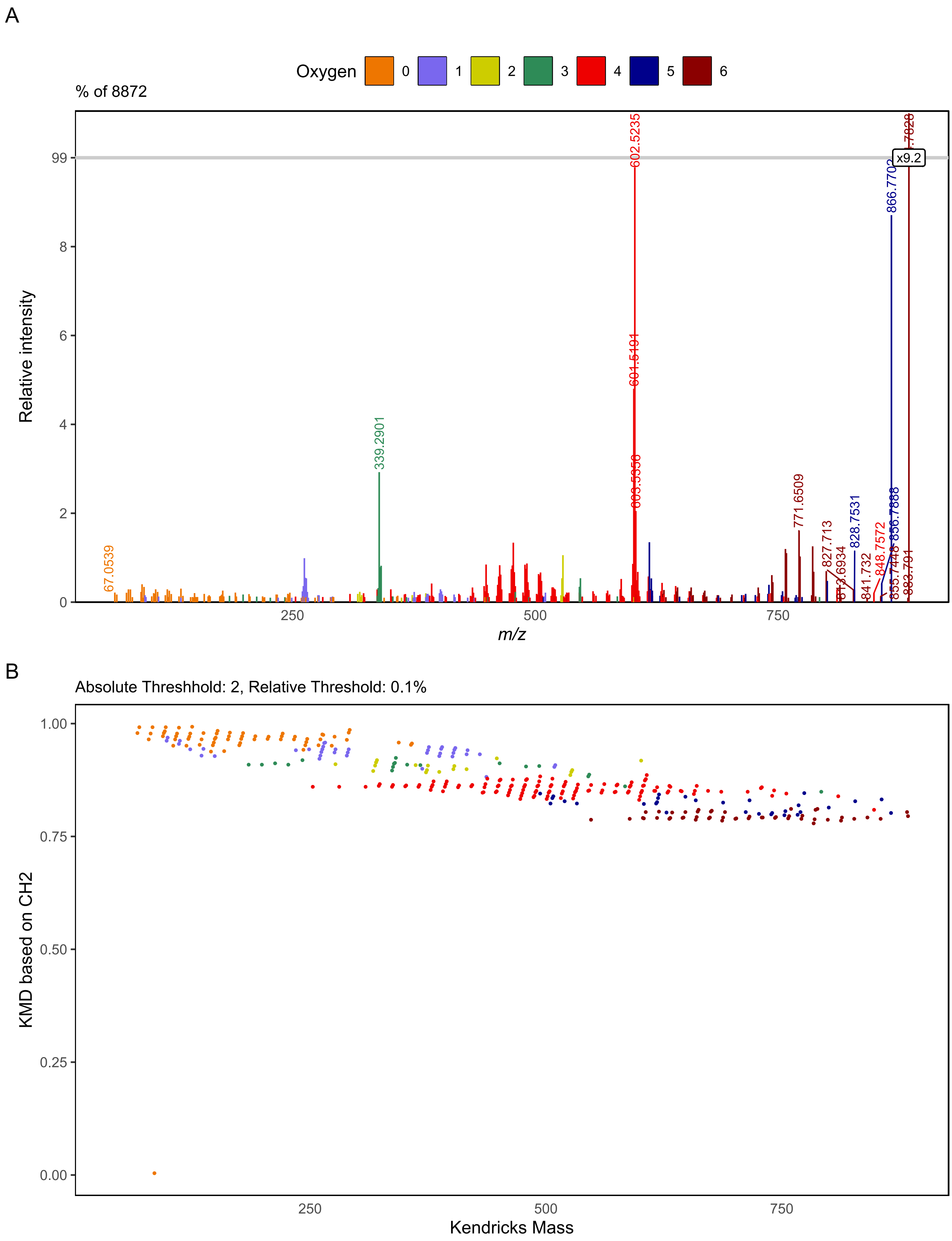

Figure S35: 35 eV CID of OOO radical cation with A) CID spectra and B) Kendricks plot from MsRadaR.

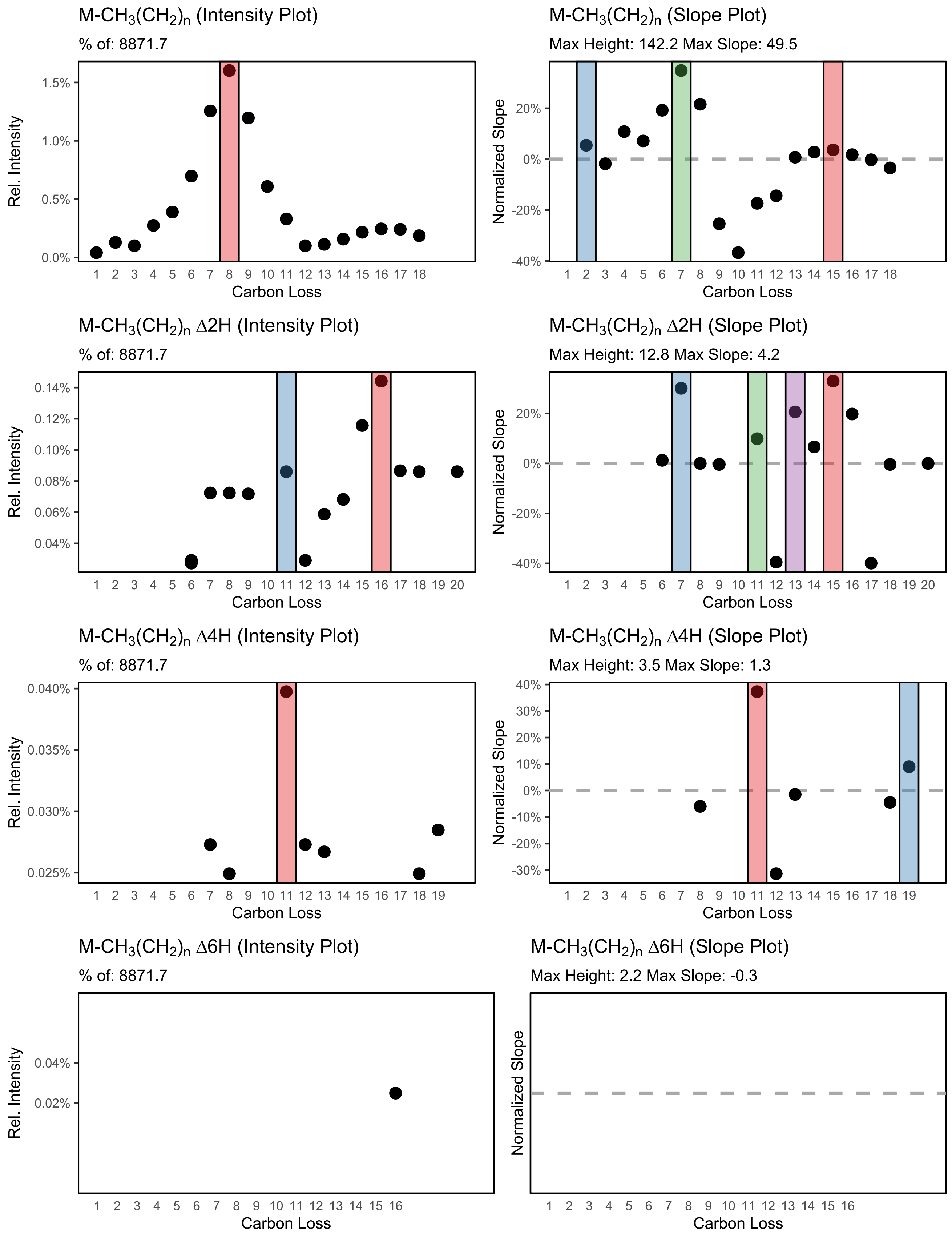

Figure S36: 35 eV CID of OOO radical cation with extracted fragmentation series starting from the precursor. Colored bars indicate intensity peak picking results from MsRadaR.

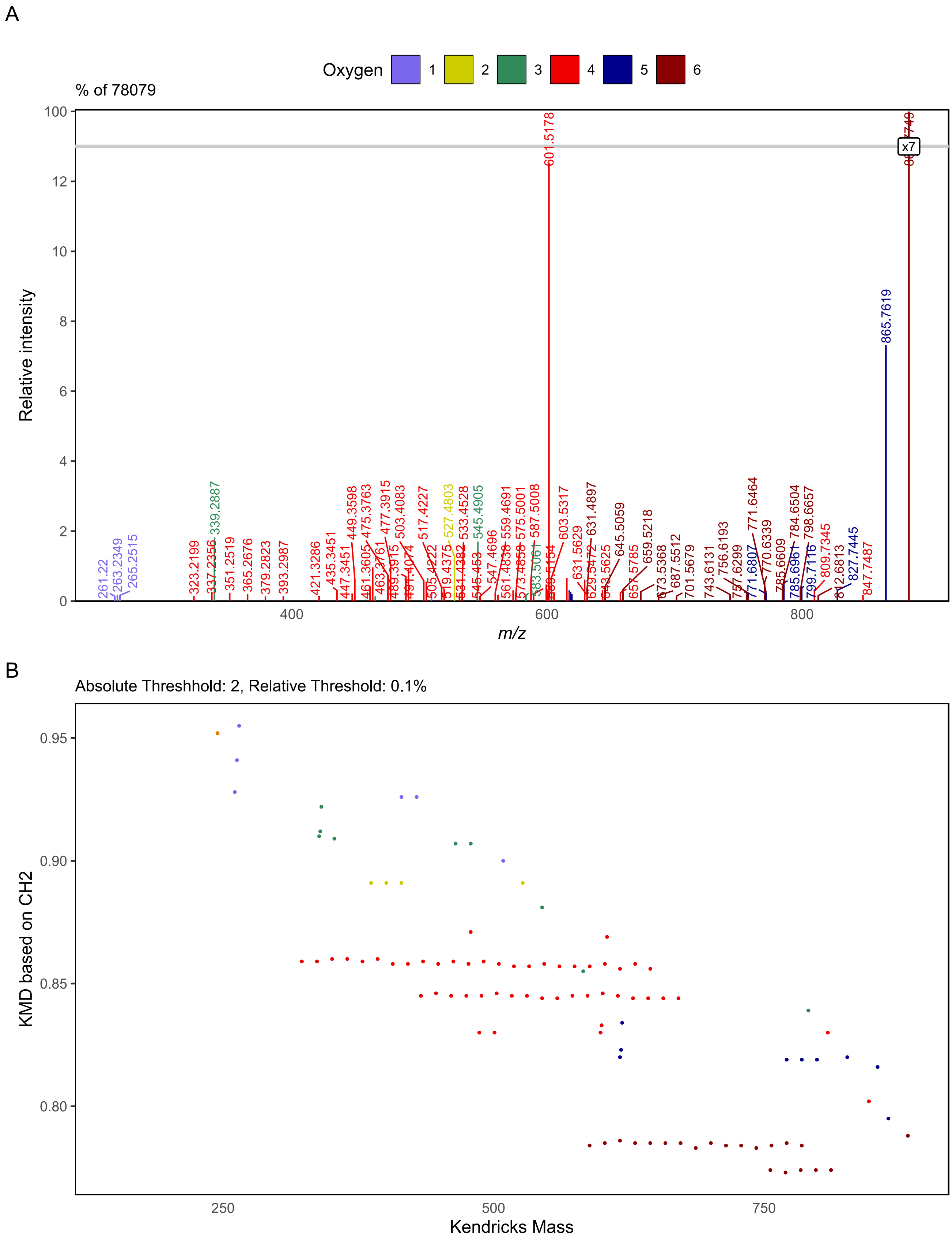

Figure S37: 35 eV CID of OOO [M-H]$^{+}$ with A) CID spectra and B) Kendricks plot from MsRadaR.

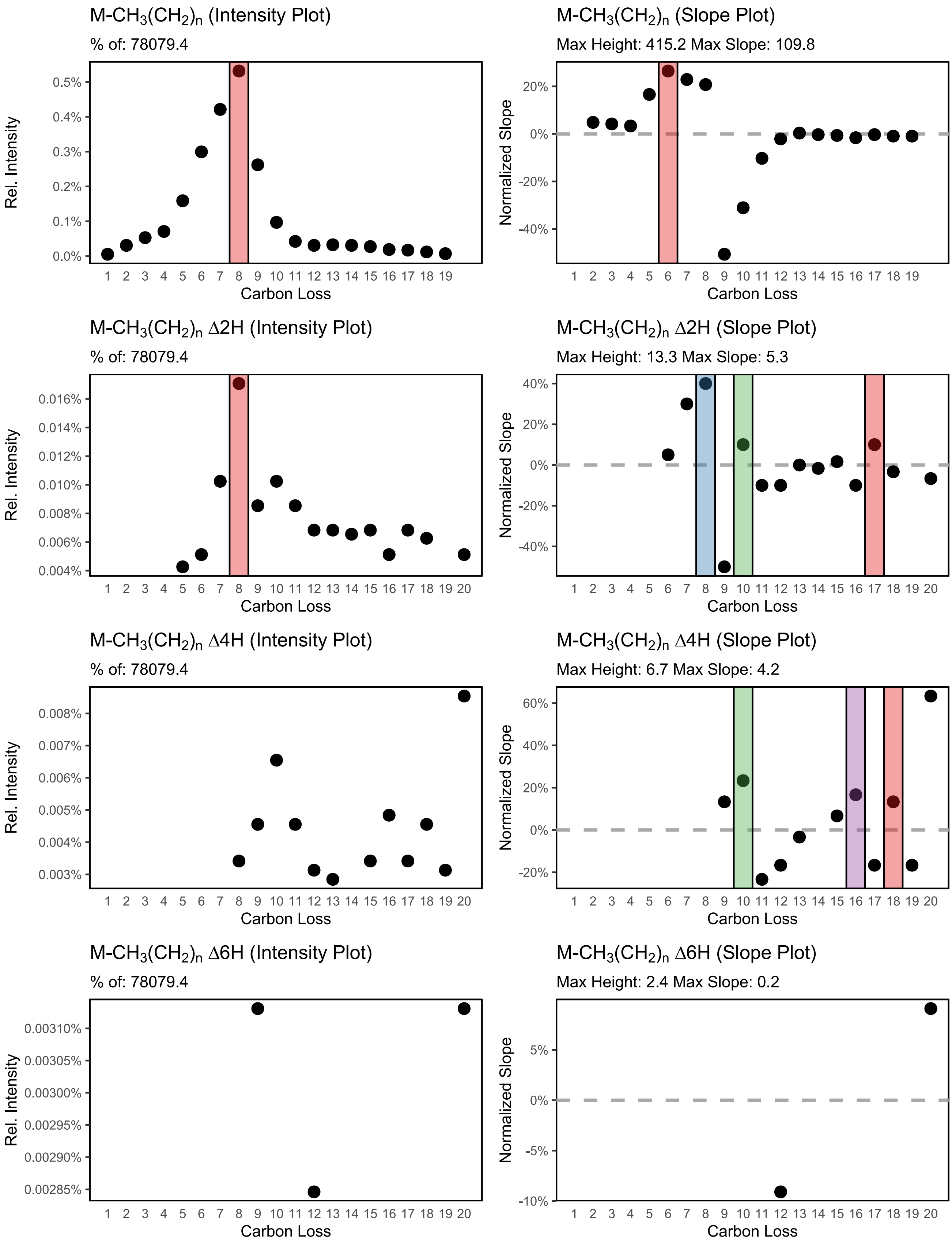

Figure S38: 35 eV CID of OOO [M-H]$^+$ with extracted fragmentation series starting from the precursor. Colored bars indicate intensity peak picking results from MsRadaR.

A

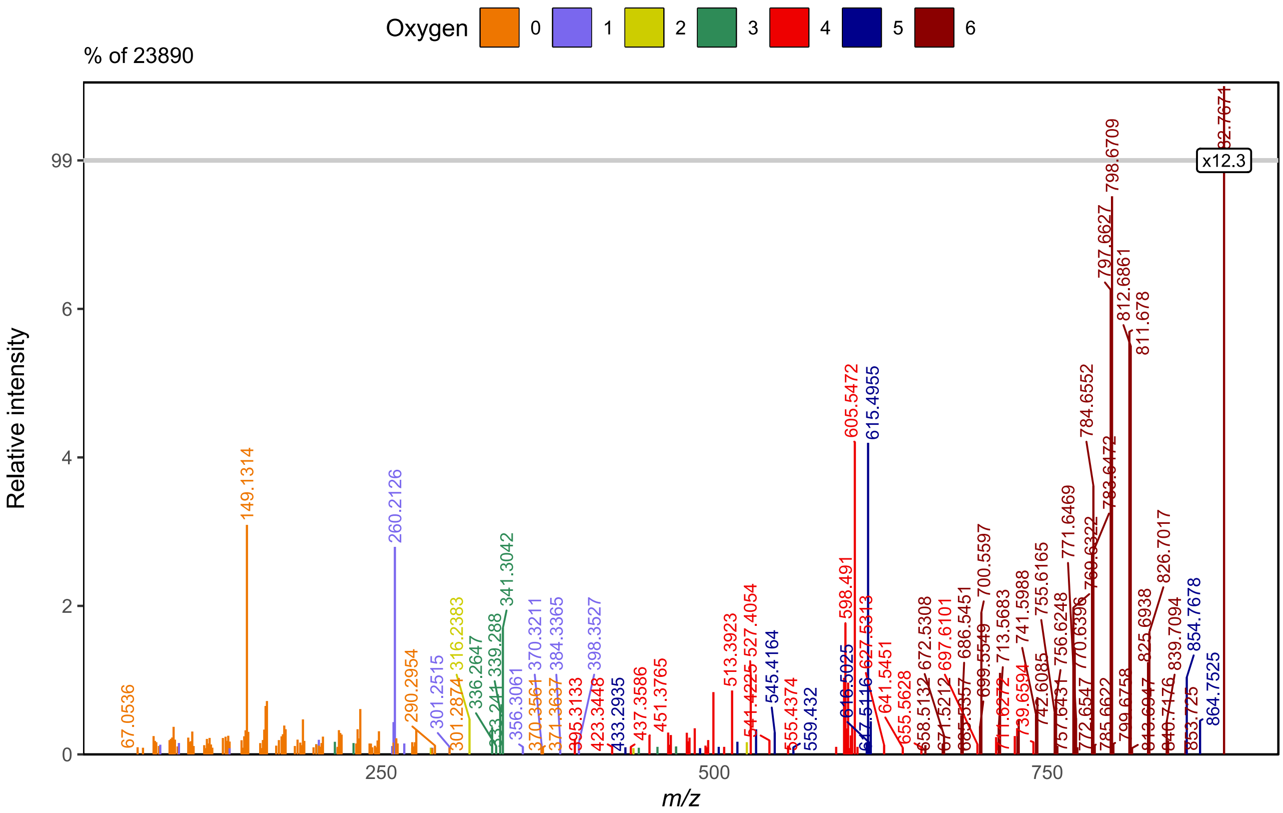

B

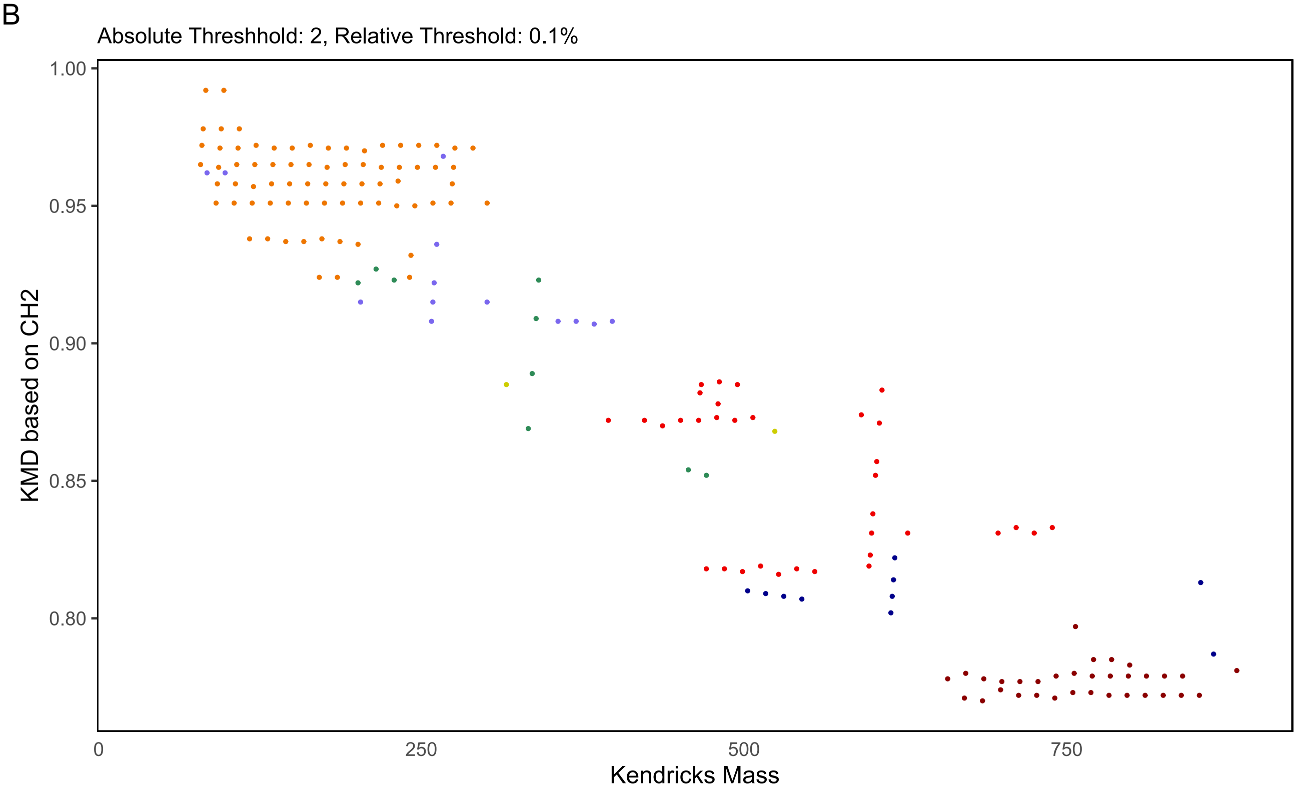

Figure S39: 35 eV CID of LSL radical cation with A) CID spectra and B) Kendricks plot from MsRadaR.

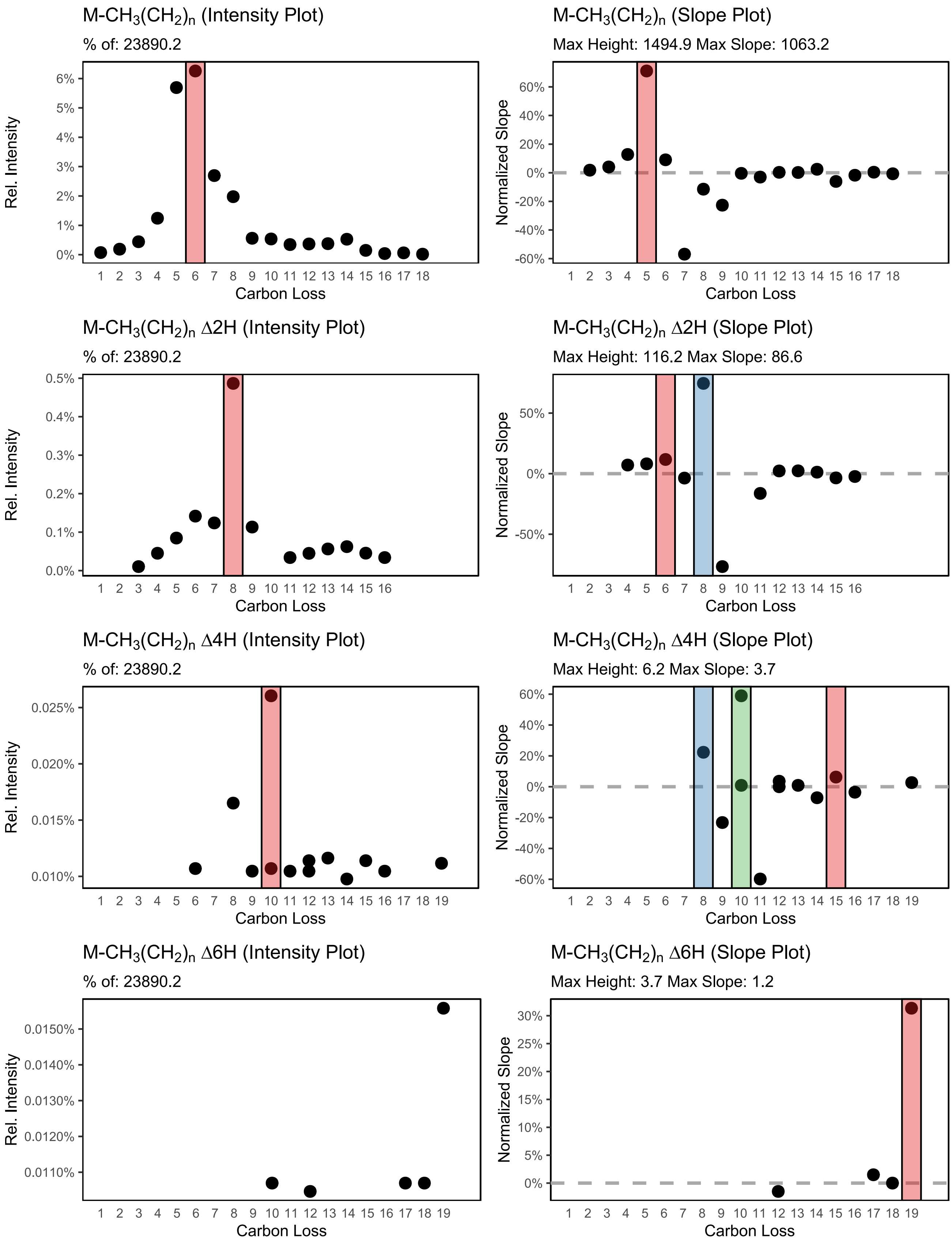

Figure S40: 35 eV CID of LSL radical cation with extracted fragmentation series starting from the precursor. Colored bars indicate intensity peak picking results from MsRadaR.

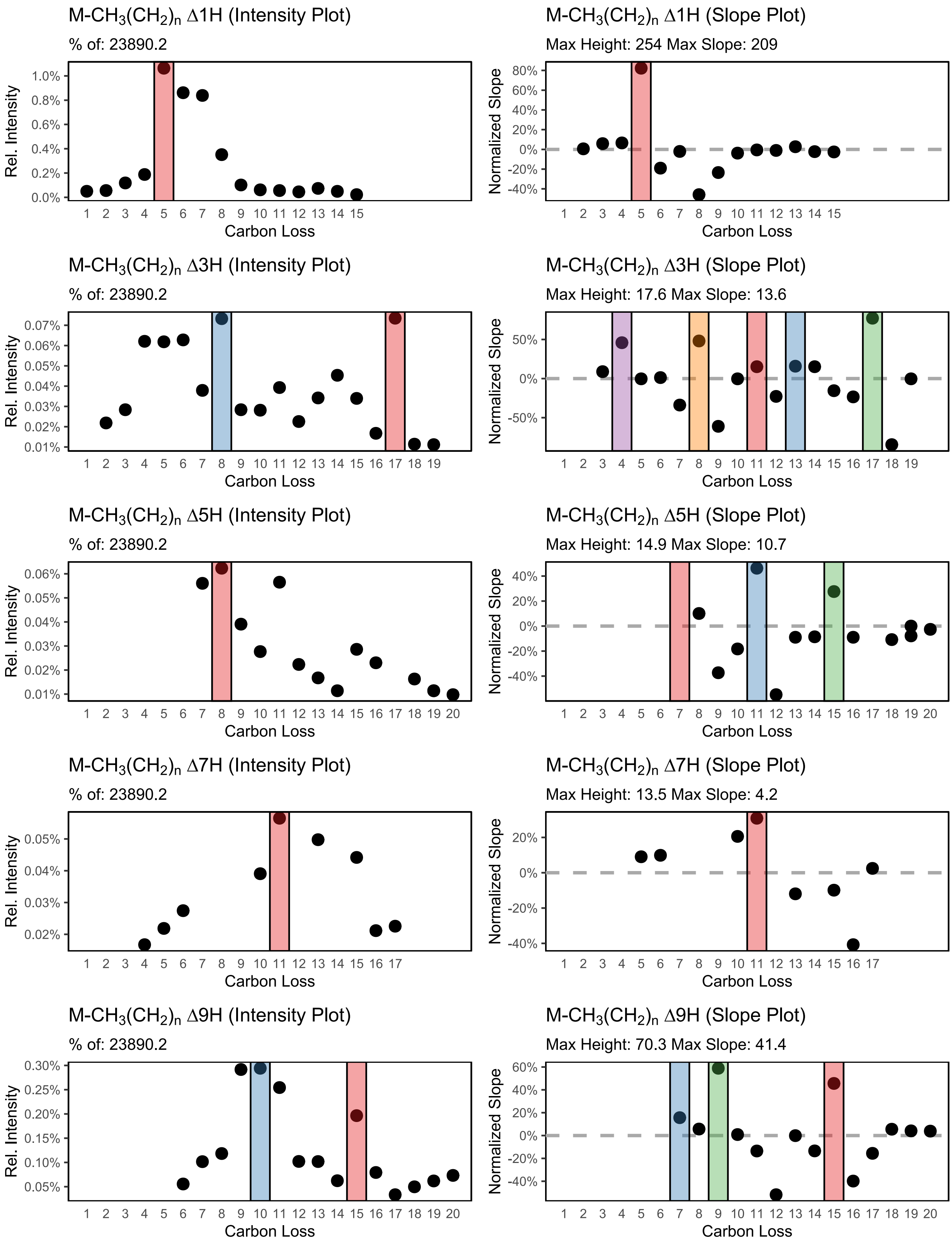

Figure S41: 35 eV CID of LSL radical cation with extracted fragmentation series starting from $[L\text{-}H_2O]^{+}$ (*m/z* 259.2225). Colored bars indicate intensity peak picking results from MsRadaR.

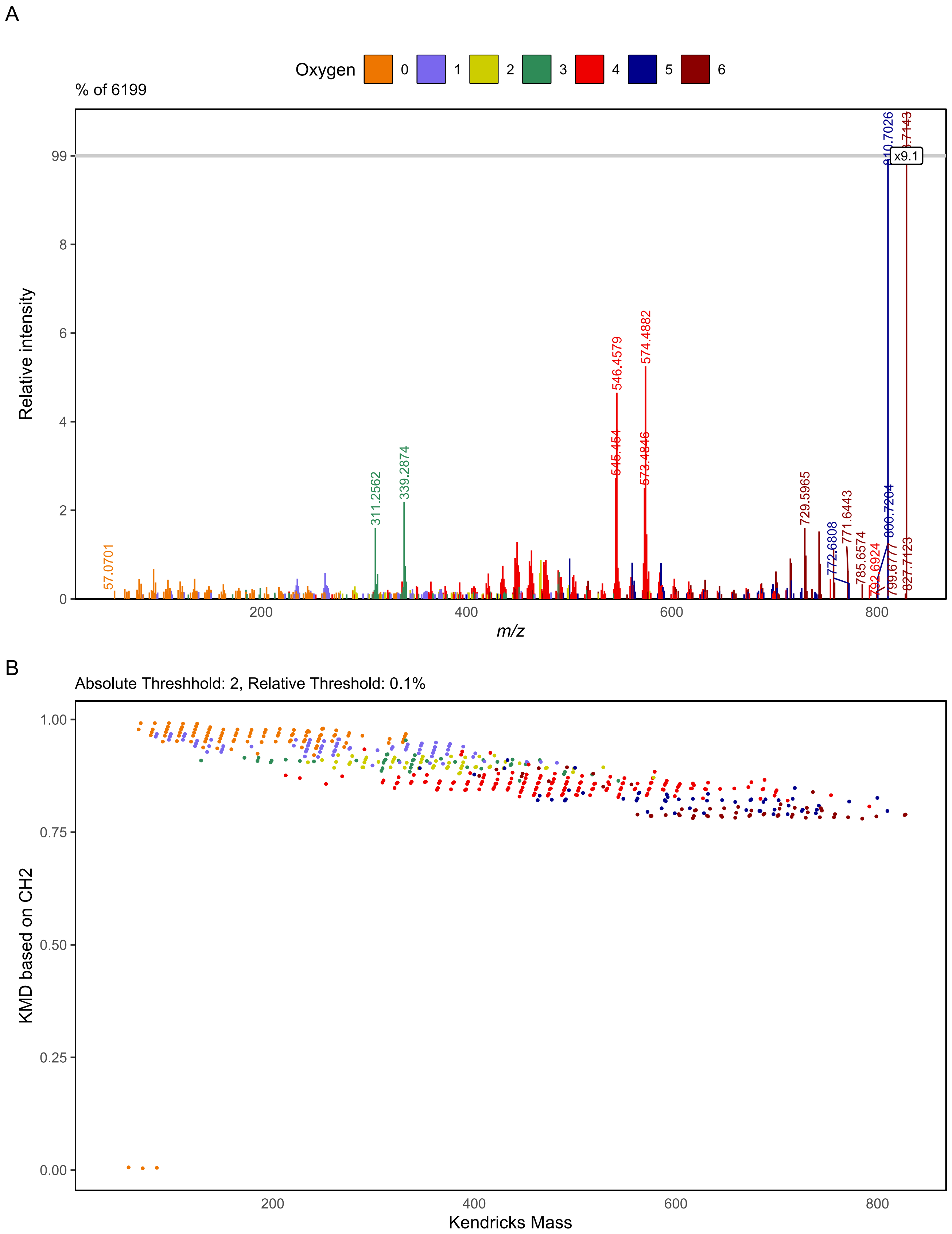

Figure S42: 35 eV CID of PoOPo radical cation with A) CID spectra and B) Kendricks plot from MsRadaR.

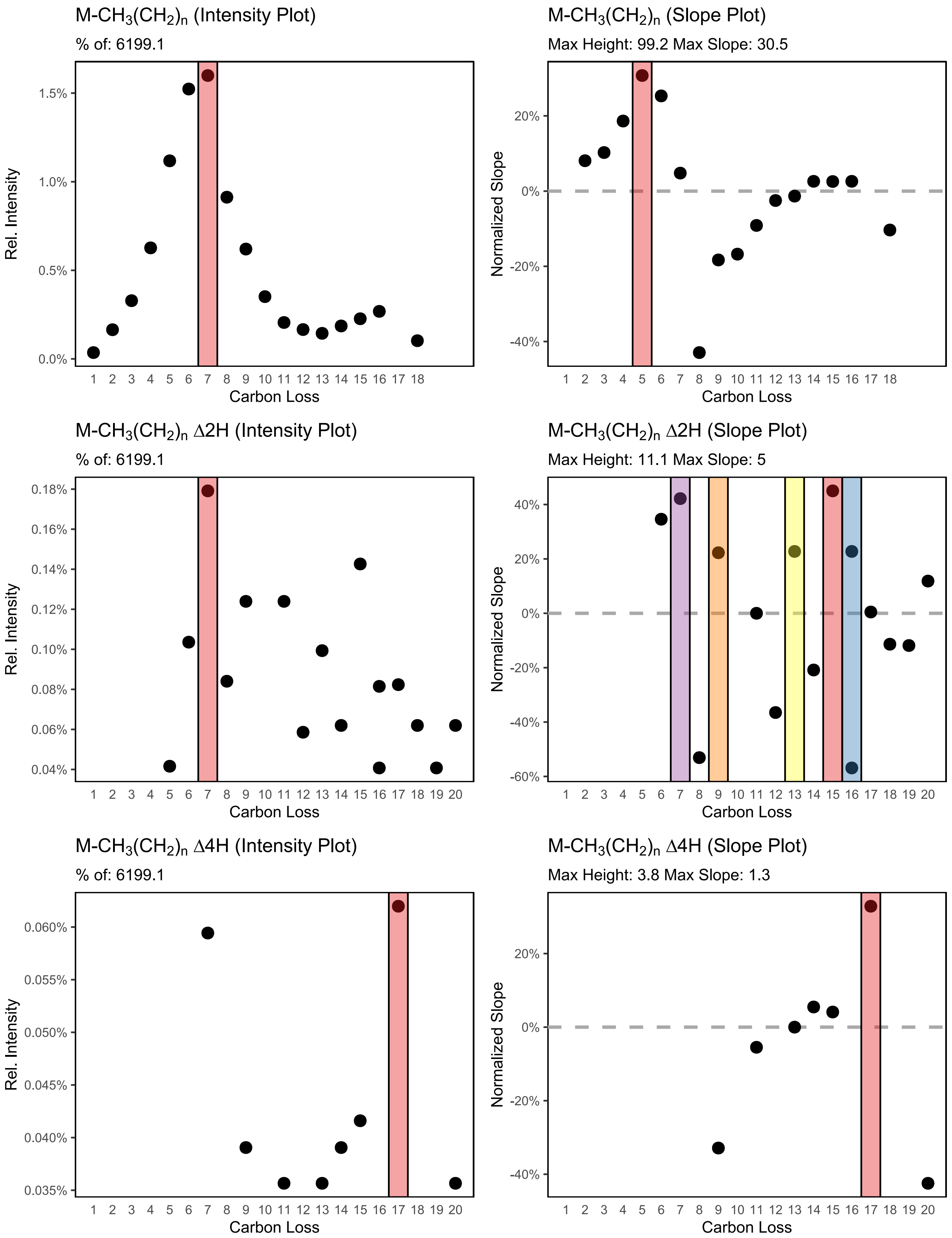

Figure S43: 35 eV CID of PoOPo radical cation with extracted fragmentation series starting from the precursor. Colored bars indicate intensity peak picking results from MsRadaR.

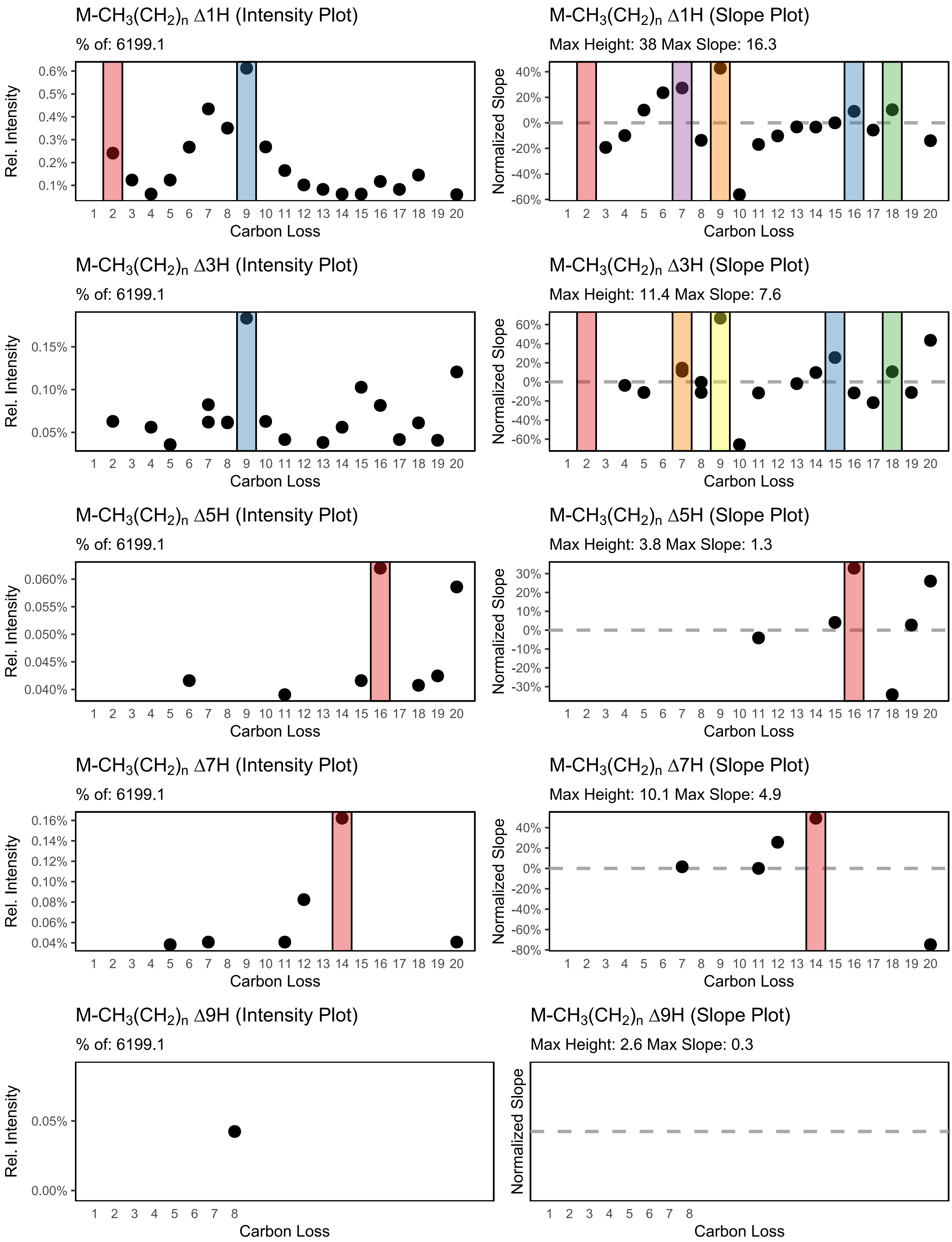

Figure S44: 35 eV CID of PoOPo radical cation with extracted fragmentation series starting from $[M\text{-}Po+2H]^+$ (*m/z* 577.5198) . Colored bars indicate intensity peak picking results from MsRadaR.

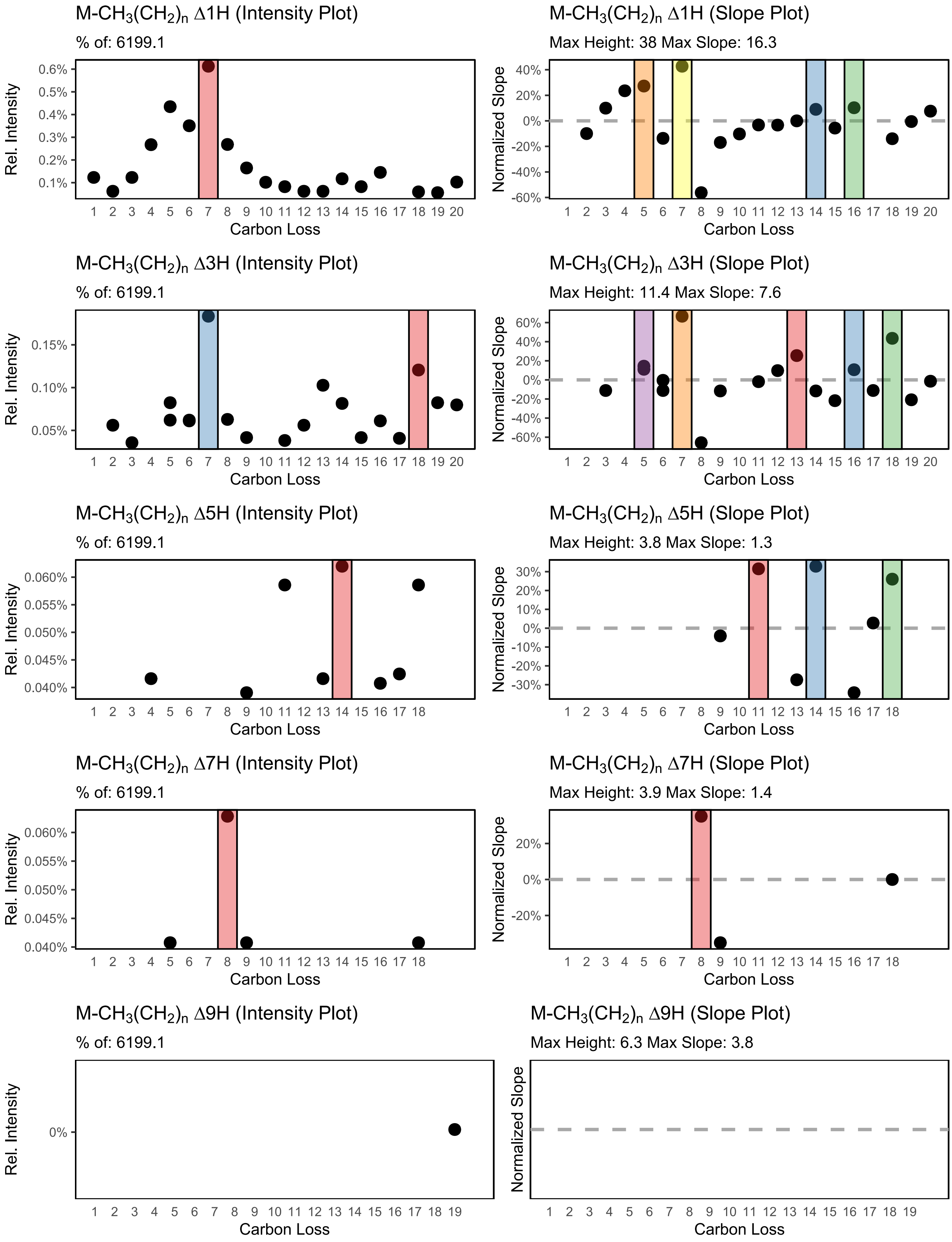

Figure S45: 35 eV CID of PoOPo radical cation with extracted fragmentation series starting from $[M-O+2H]^+$ (*m/z* 549.4877). Colored bars indicate intensity peak picking results from MsRadaR.

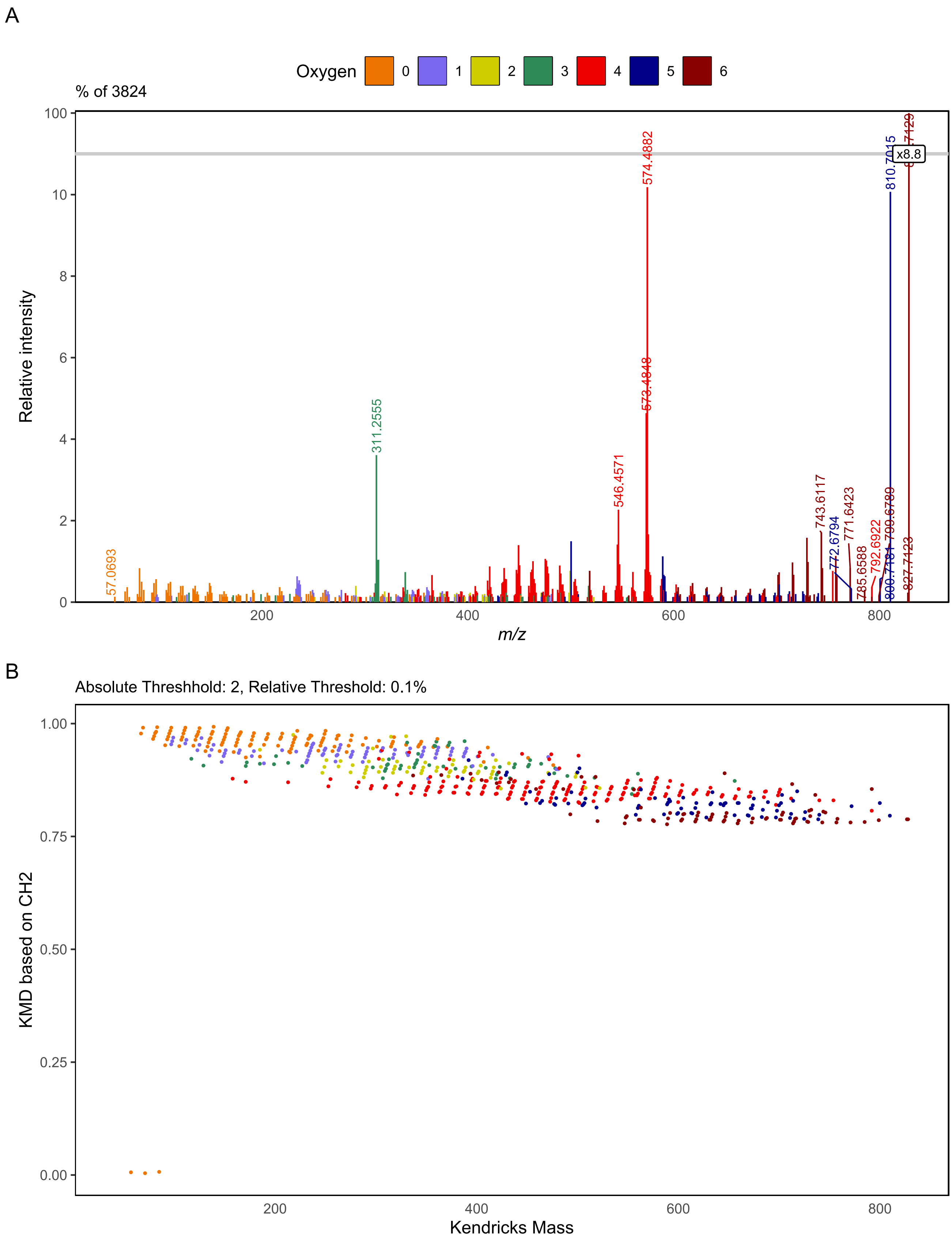

Figure S46: 35 eV CID of PoPoO radical cation with A) CID spectra and B) Kendricks plot from MsRadaR.

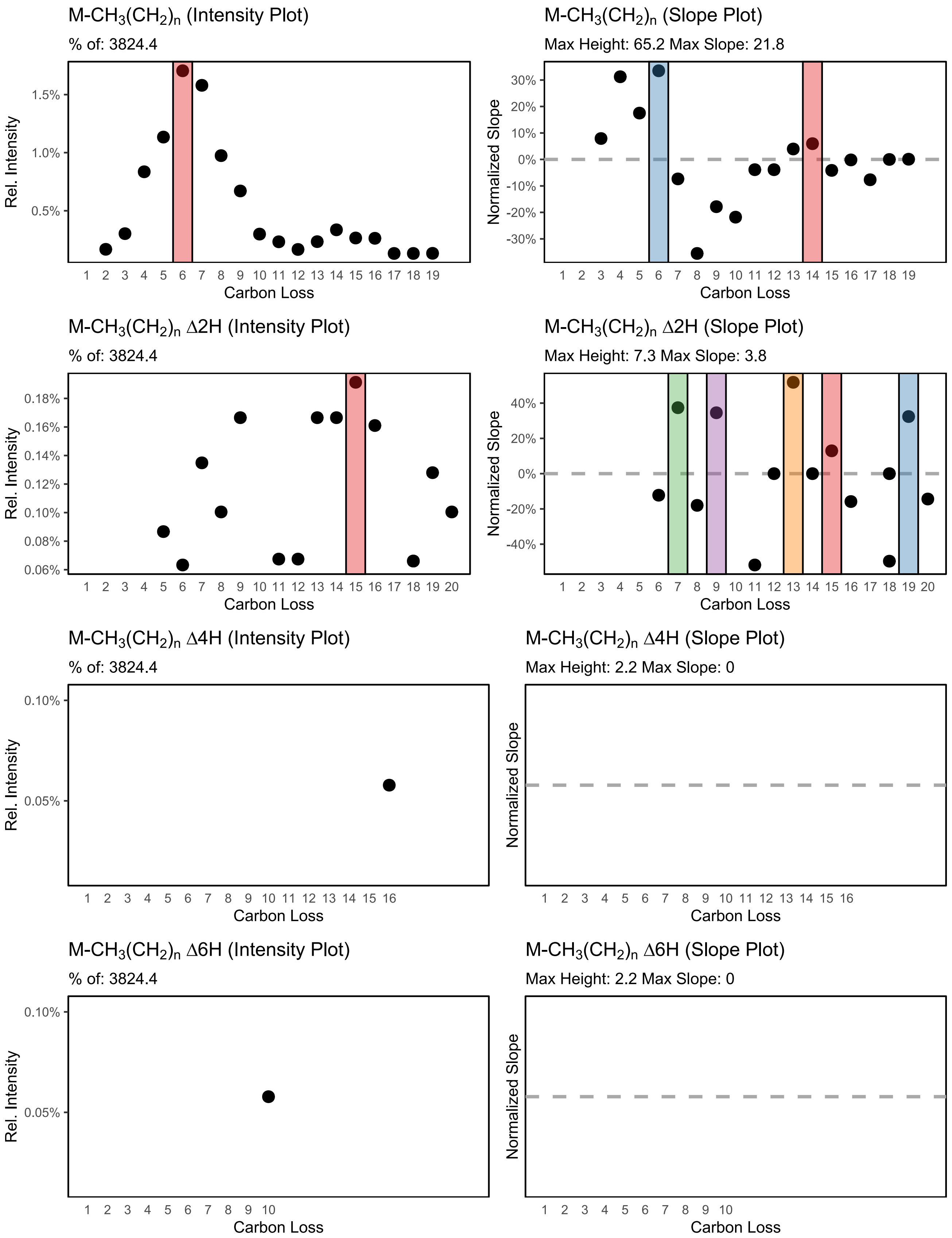

Figure S47: 35 eV CID of PoPoO radical cation with extracted fragmentation series starting from the precursor. Colored bars indicate intensity peak picking results from MsRadaR.

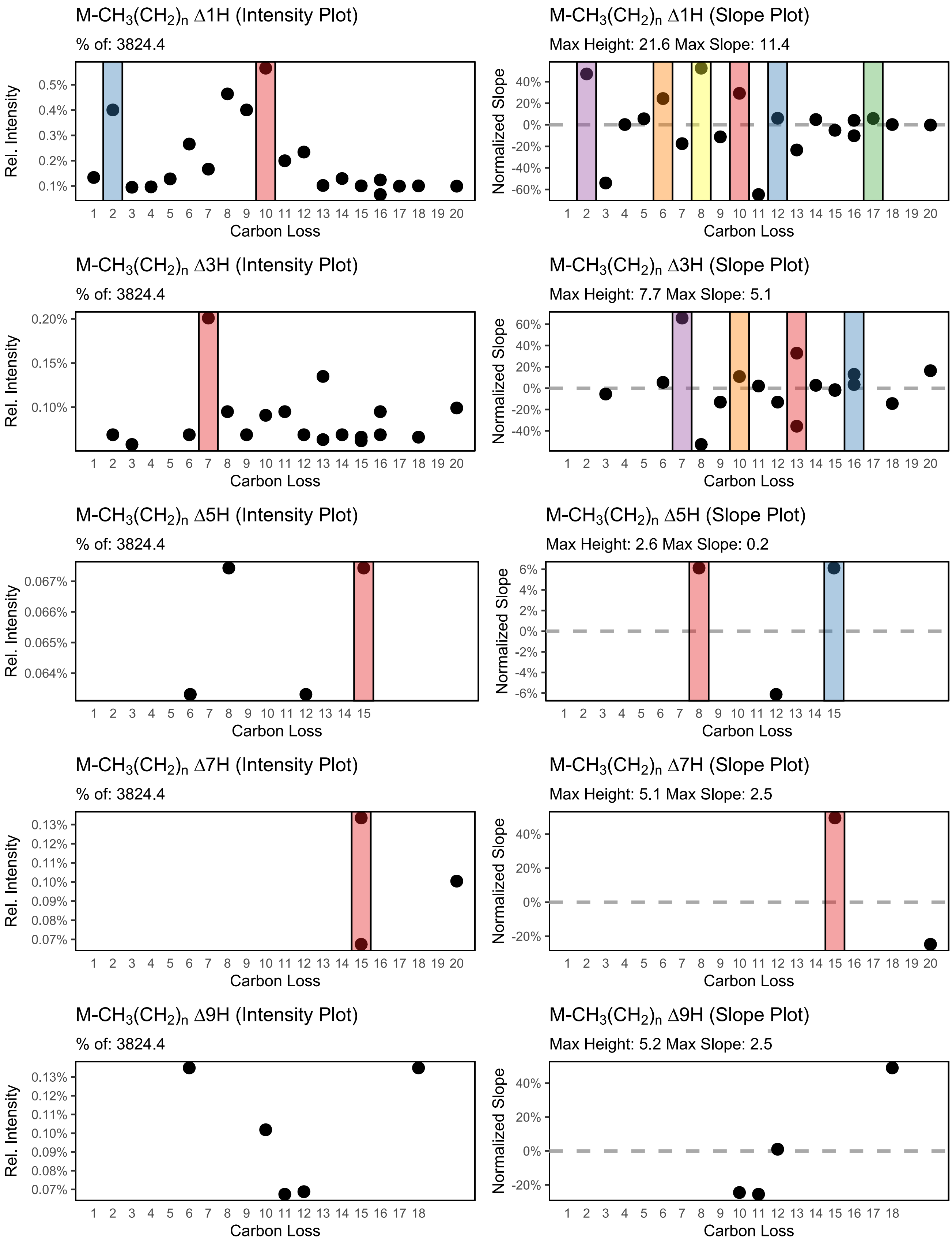

Figure S48: 35 eV CID of PoPoO radical cation with extracted fragmentation series starting from $[M-Po+2H]^+$ (*m/z* 577.5198). Colored bars indicate intensity peak picking results from MsRadaR.

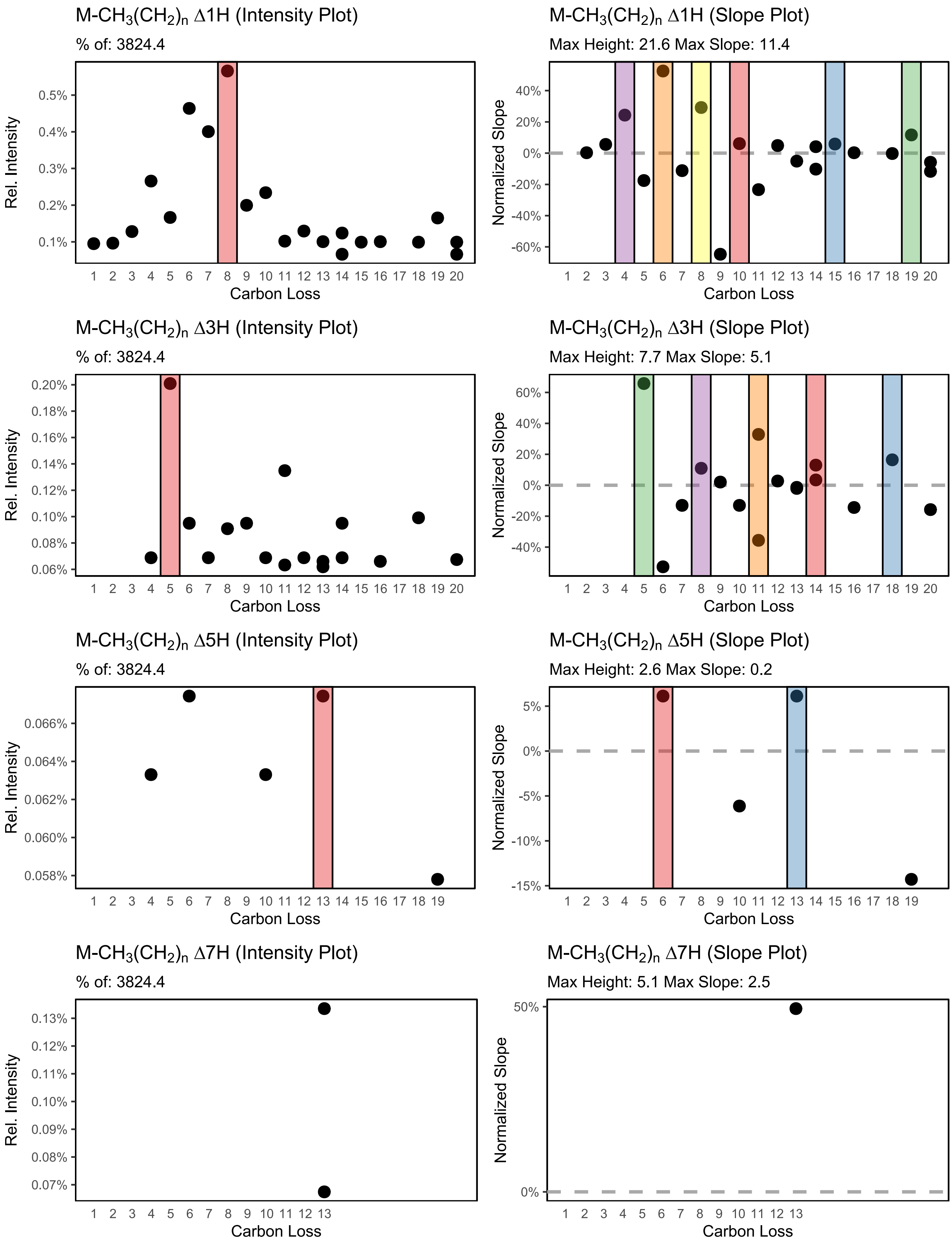

Figure S49: 35 eV CID of PoPoO radical cation with extracted fragmentation series starting from $[M\text{-}O+2H]^+$ (*m/z* 549.4877). Colored bars indicate intensity peak picking results from MsRadaR.

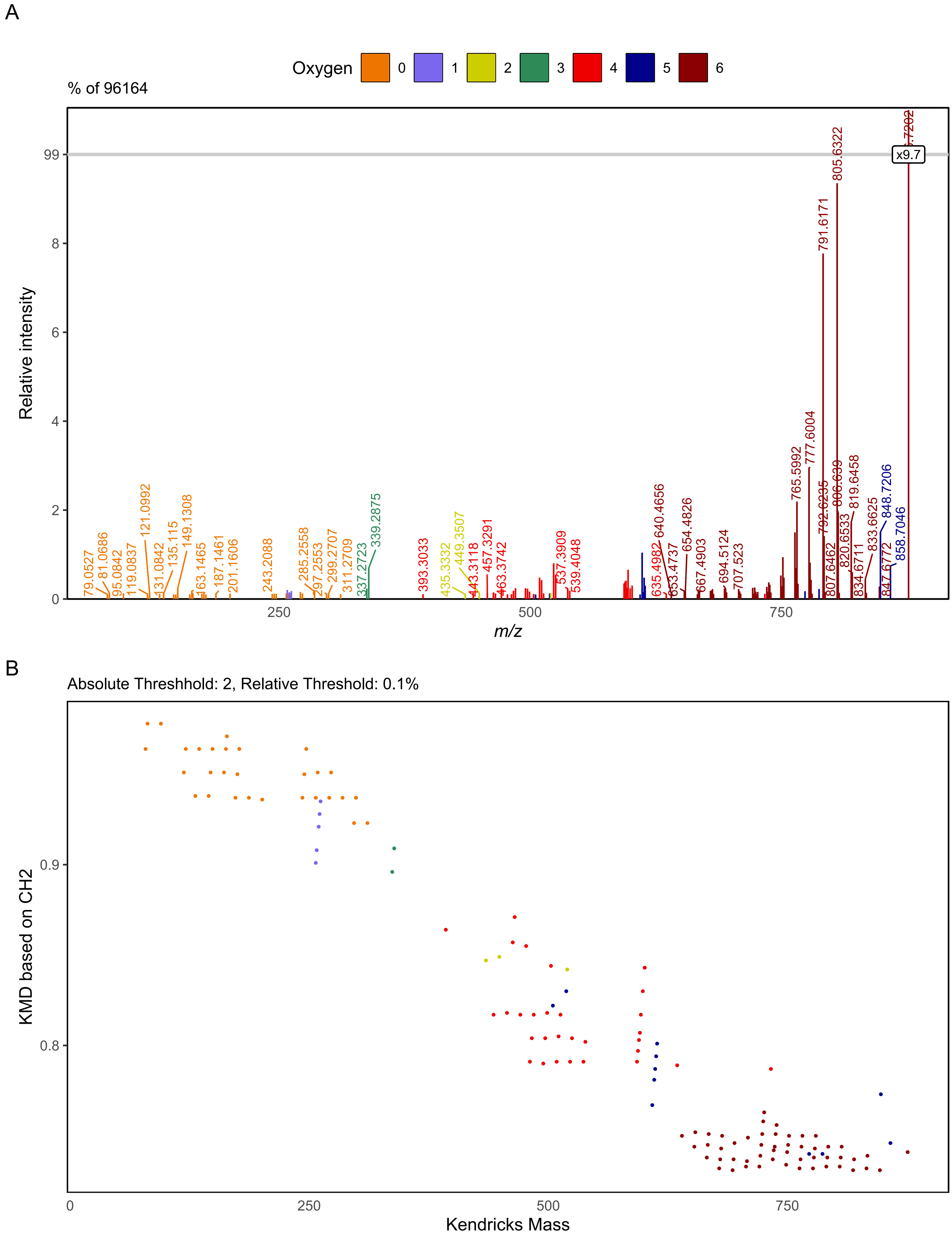

Figure S50: 35 eV CID of LLgLn radical cation with A) CID spectra and B) Kendricks plot from MsRadaR.

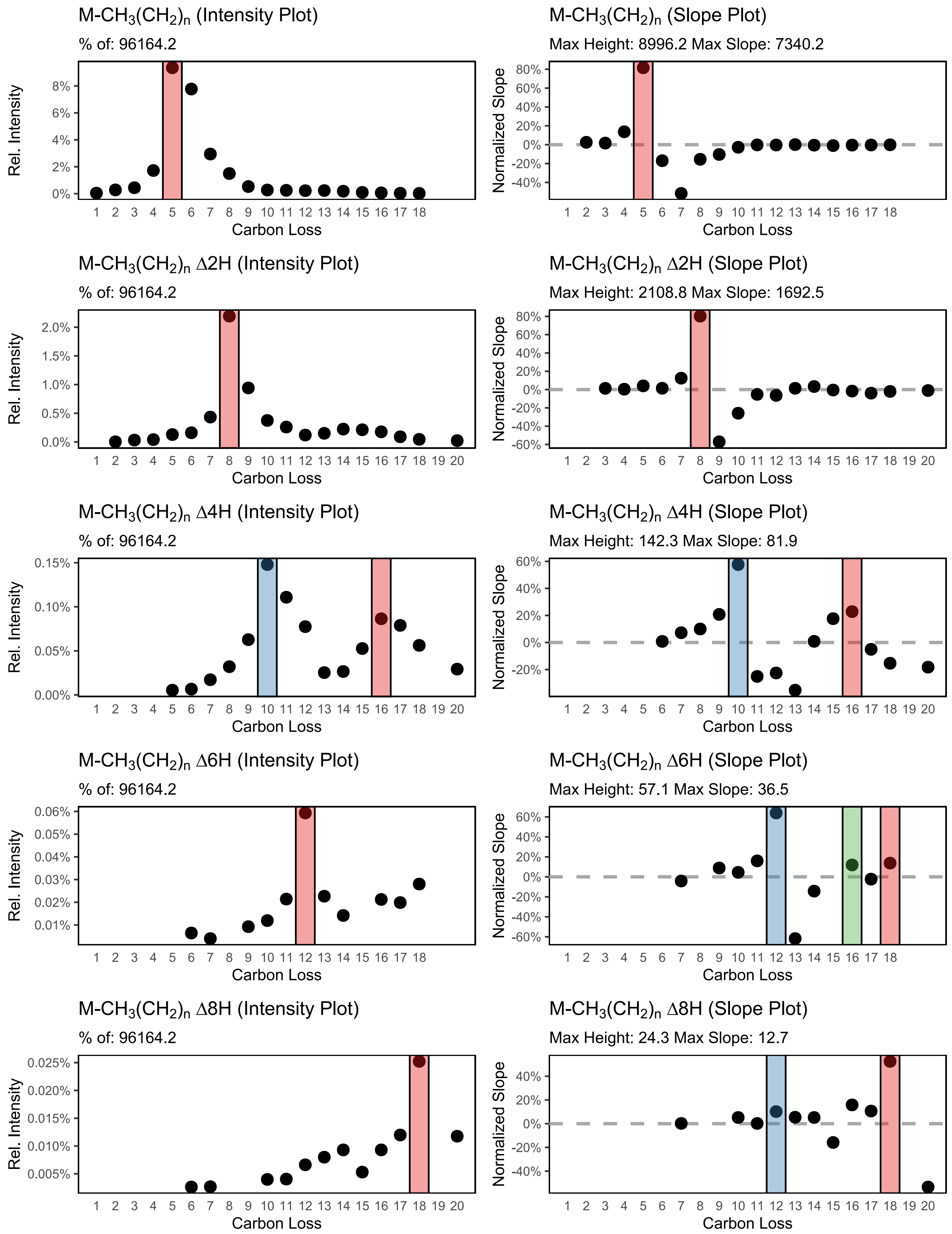

Figure S51: 35 eV CID of LLgLn radical cation with extracted fragmentation series starting from the precursor. Colored bars indicate intensity peak picking results from MsRadaR.

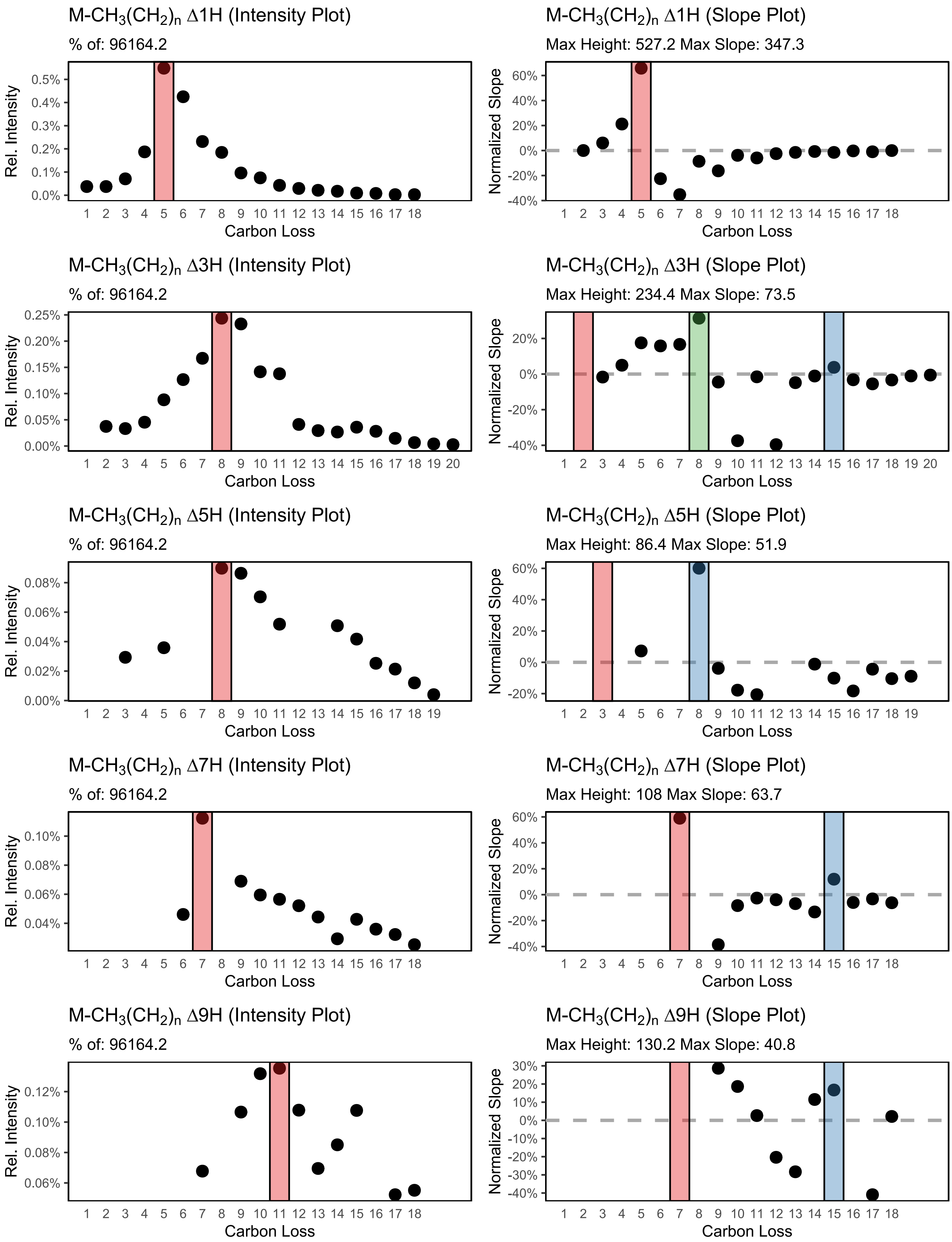

Figure S52: 35 eV CID of LLgLn radical cation with extracted fragmentation series starting from $[M-L+2H]^+$ (*m/z* 599.5034). Colored bars indicate intensity peak picking results from MsRadaR.

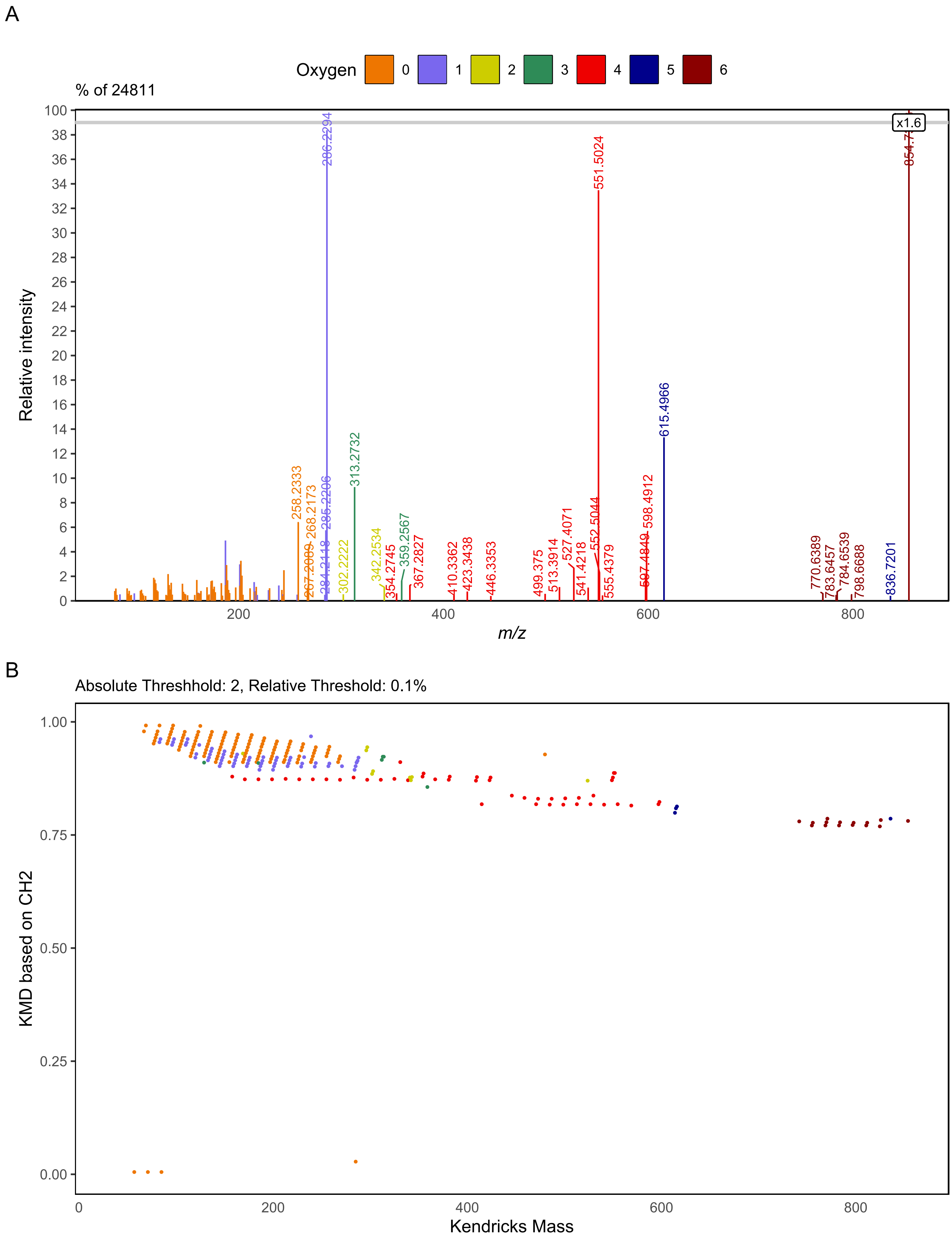

Figure S53: 35 eV CID of PArP radical cation with A) CID spectra and B) Kendricks plot from MsRadaR.

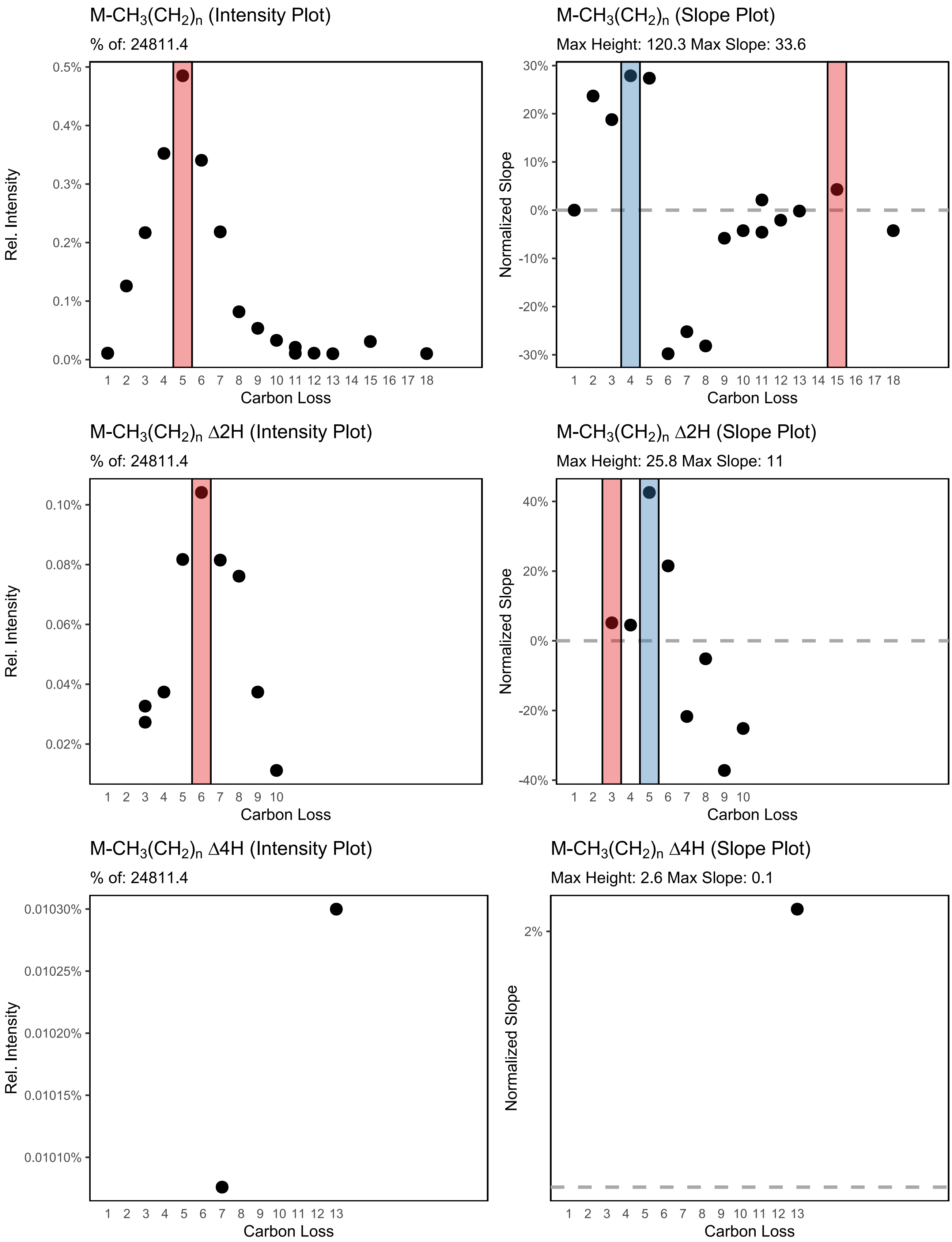

Figure S54: 35 eV CID of PArP radical cation with extracted fragmentation series starting from the precursor. Colored bars indicate intensity peak picking results from MsRadaR.

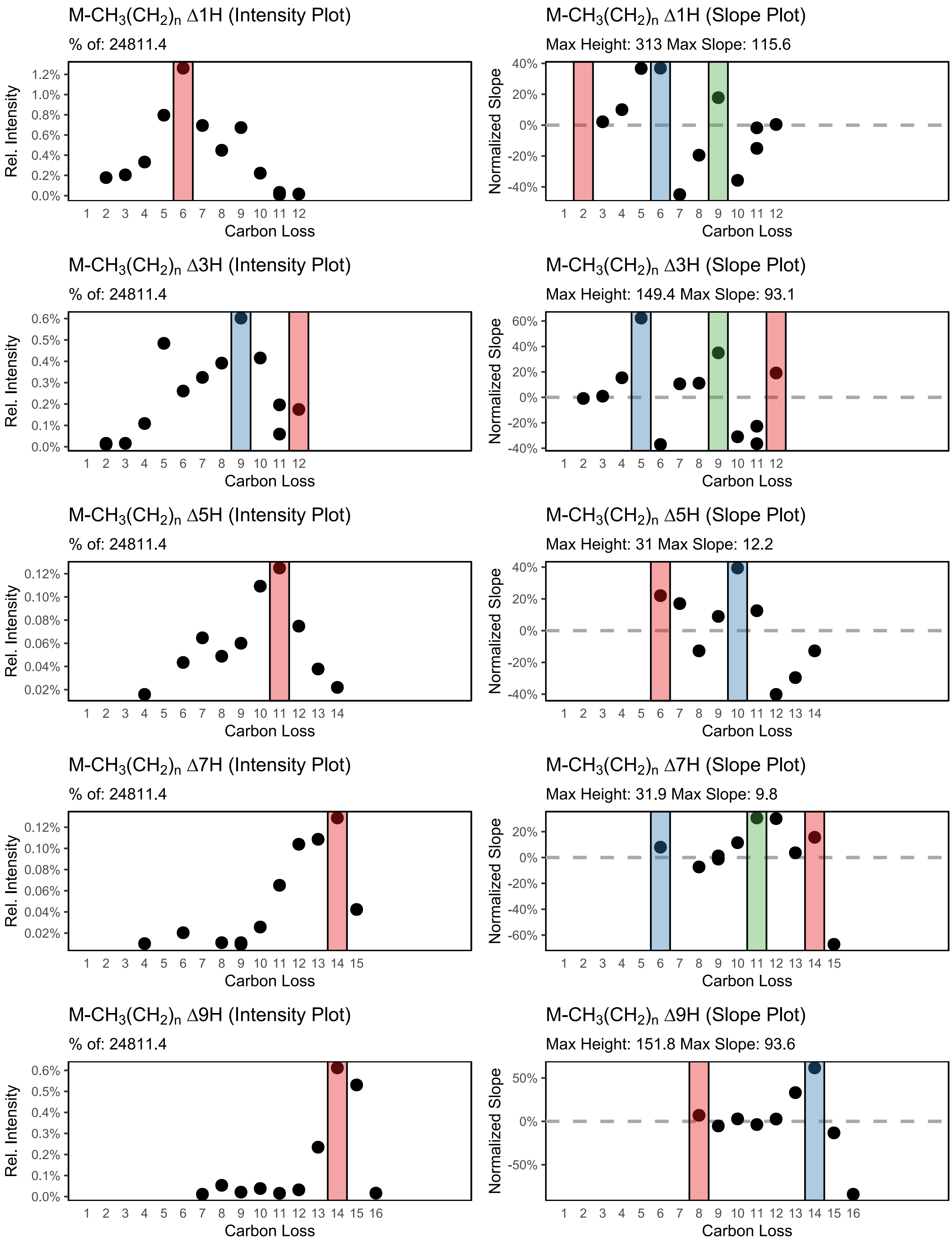

Figure S55: 35 eV CID of PArP radical cation with extracted fragmentation series starting from $[Ar\text{-}H_2O]^+$ (*m/z* 286.2294). Colored bars indicate intensity peak picking results from MsRadaR.

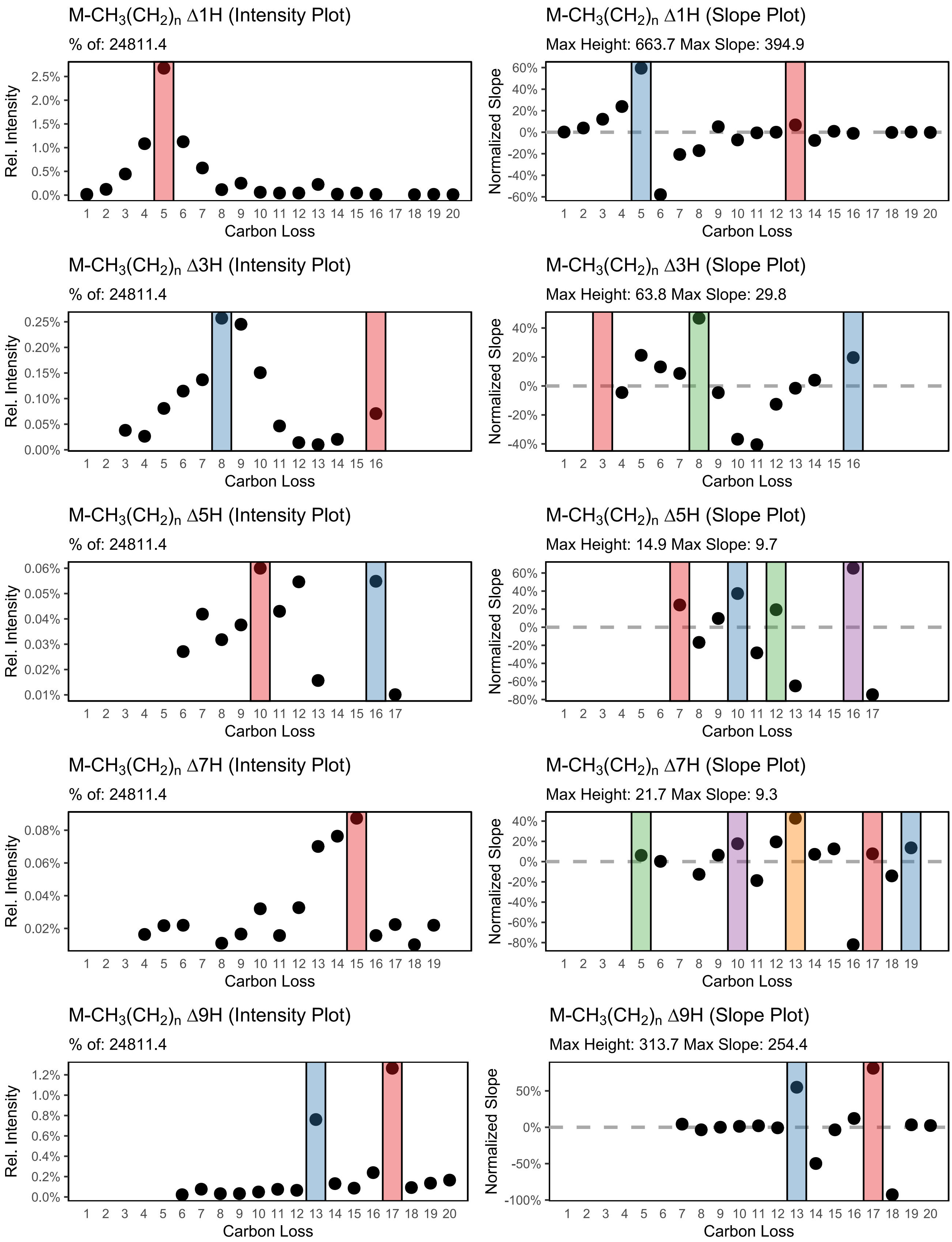

Figure S56: 35 eV CID of PArP radical cation with extracted fragmentation series starting from $[M-P-2H]^+$ (*m/z* 597.4877). Colored bars indicate intensity peak picking results from MsRadaR.

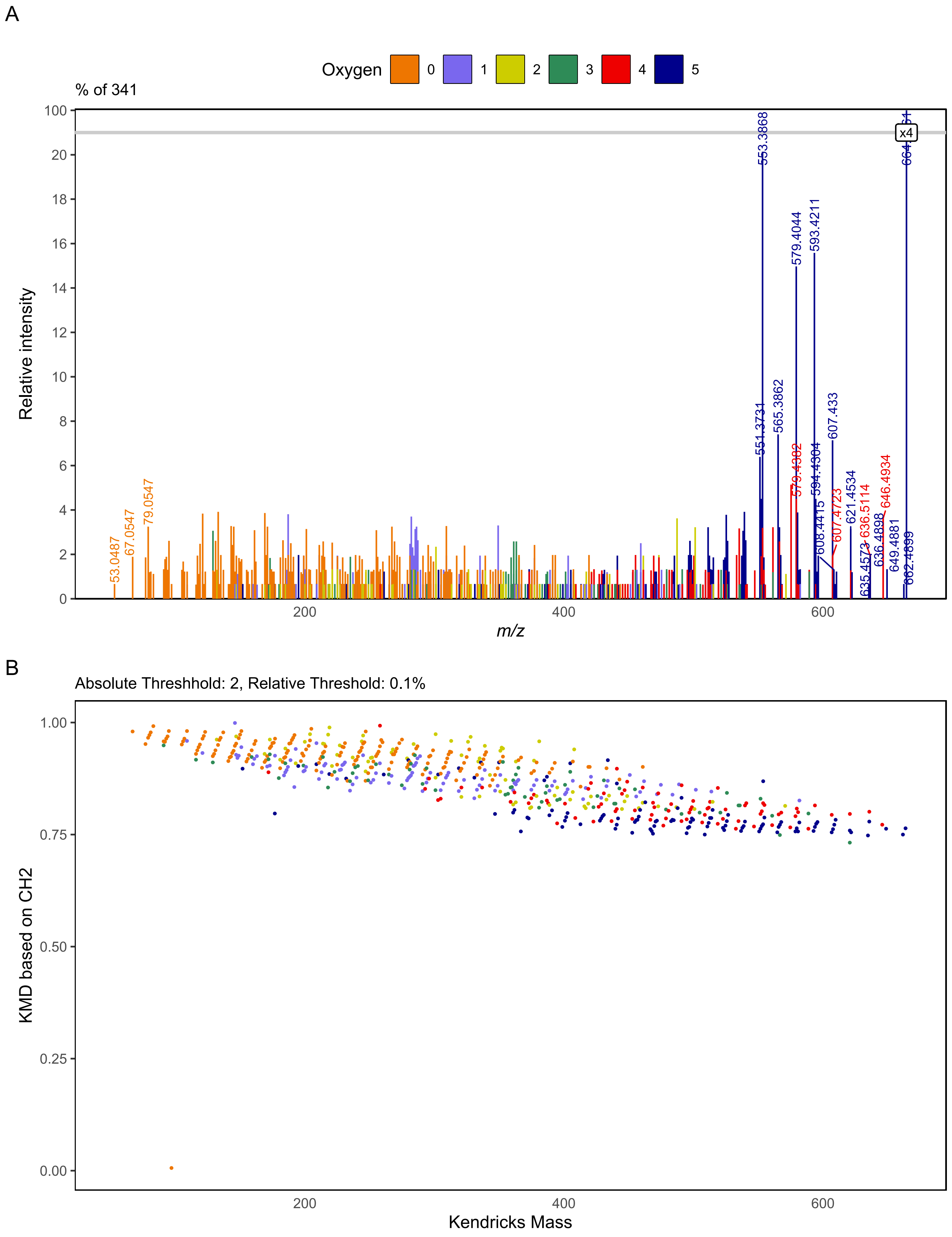

Figure S57: 35 eV CID of 1,3ArAr radical cation with A) CID spectra and B) Kendricks plot from MsRadaR.

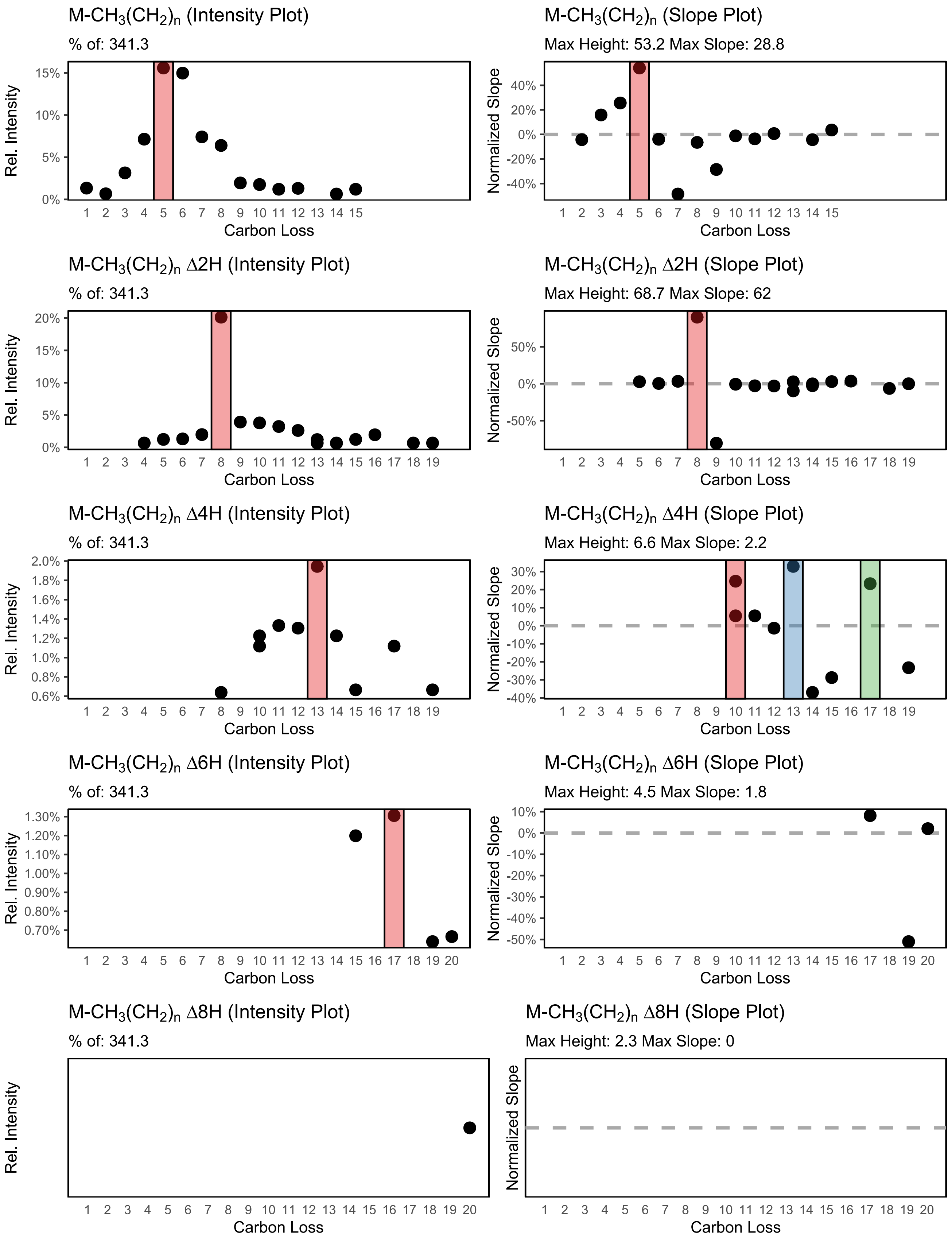

Figure S58: 35 eV CID of 1,3ArAr radical cation with extracted fragmentation series starting from the precursor. Colored bars indicate intensity peak picking results from MsRadaR.

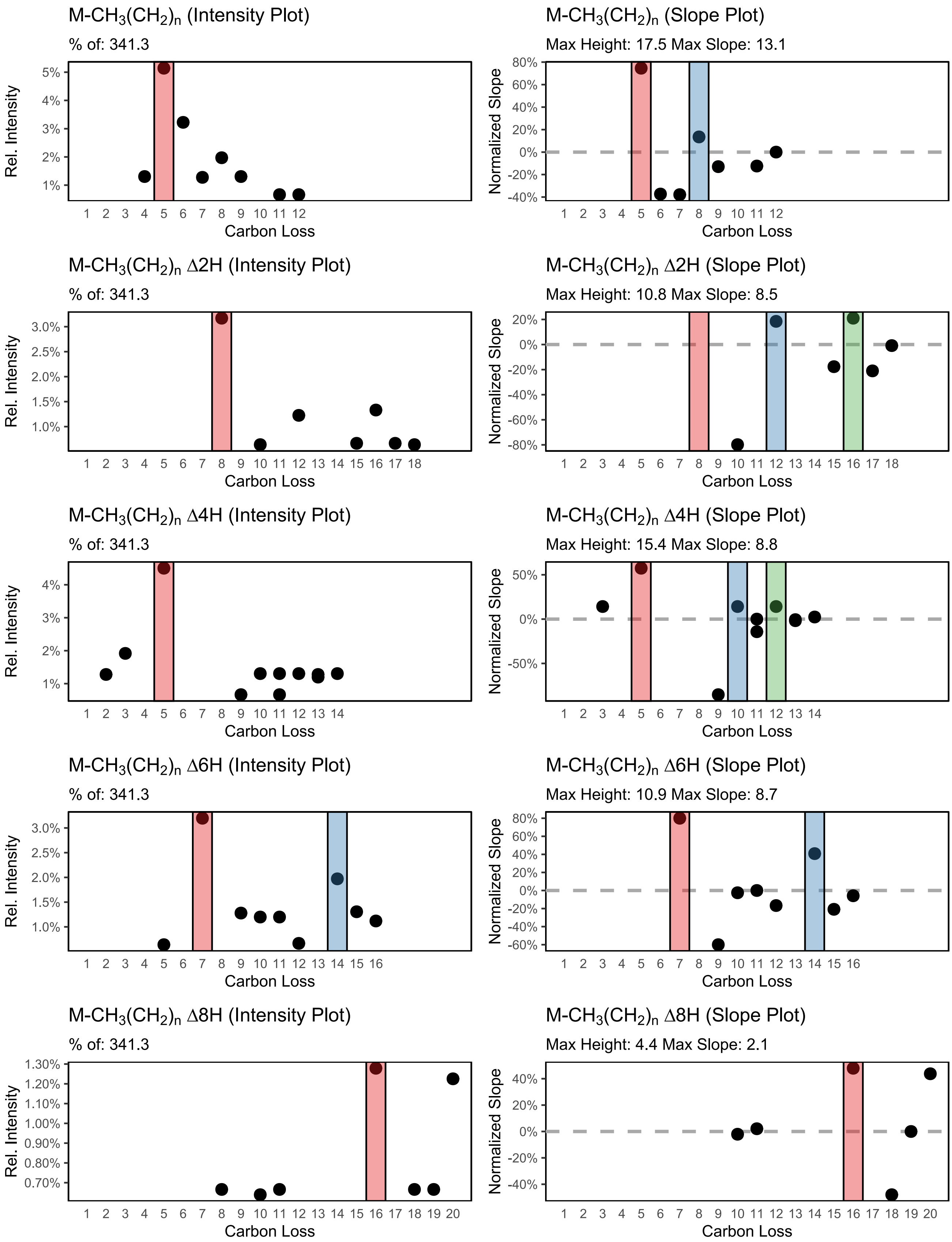

Figure S59: 35 eV CID of 1,3ArAr radical cation with extracted fragmentation series starting from $[M-H_2O]^+$ (*m/z* 646.4956). Colored bars indicate intensity peak picking results from MsRadaR.

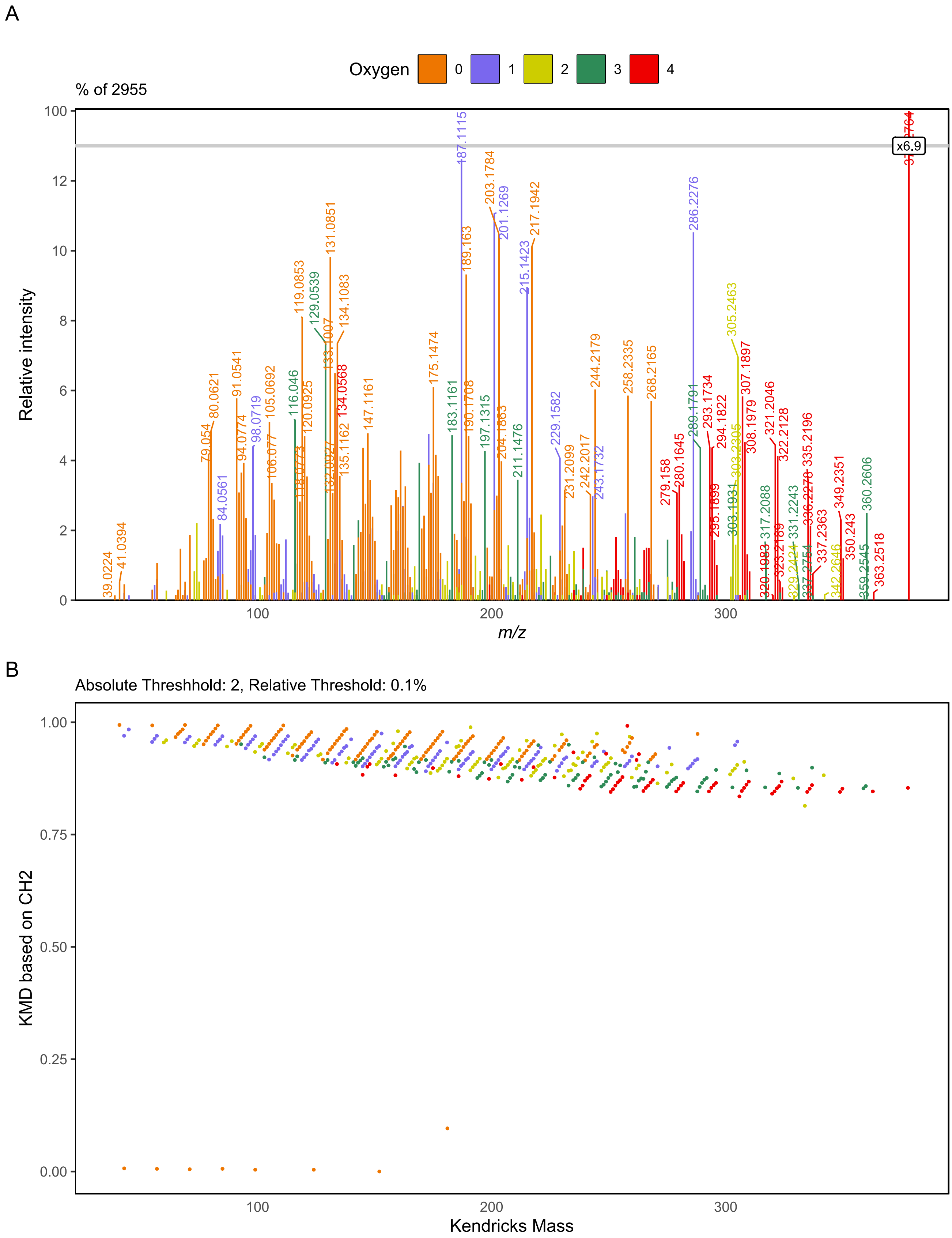

Figure S60: 20 eV CID of 2Ar radical cation with A) CID spectra and B) Kendricks plot from MsRadaR.

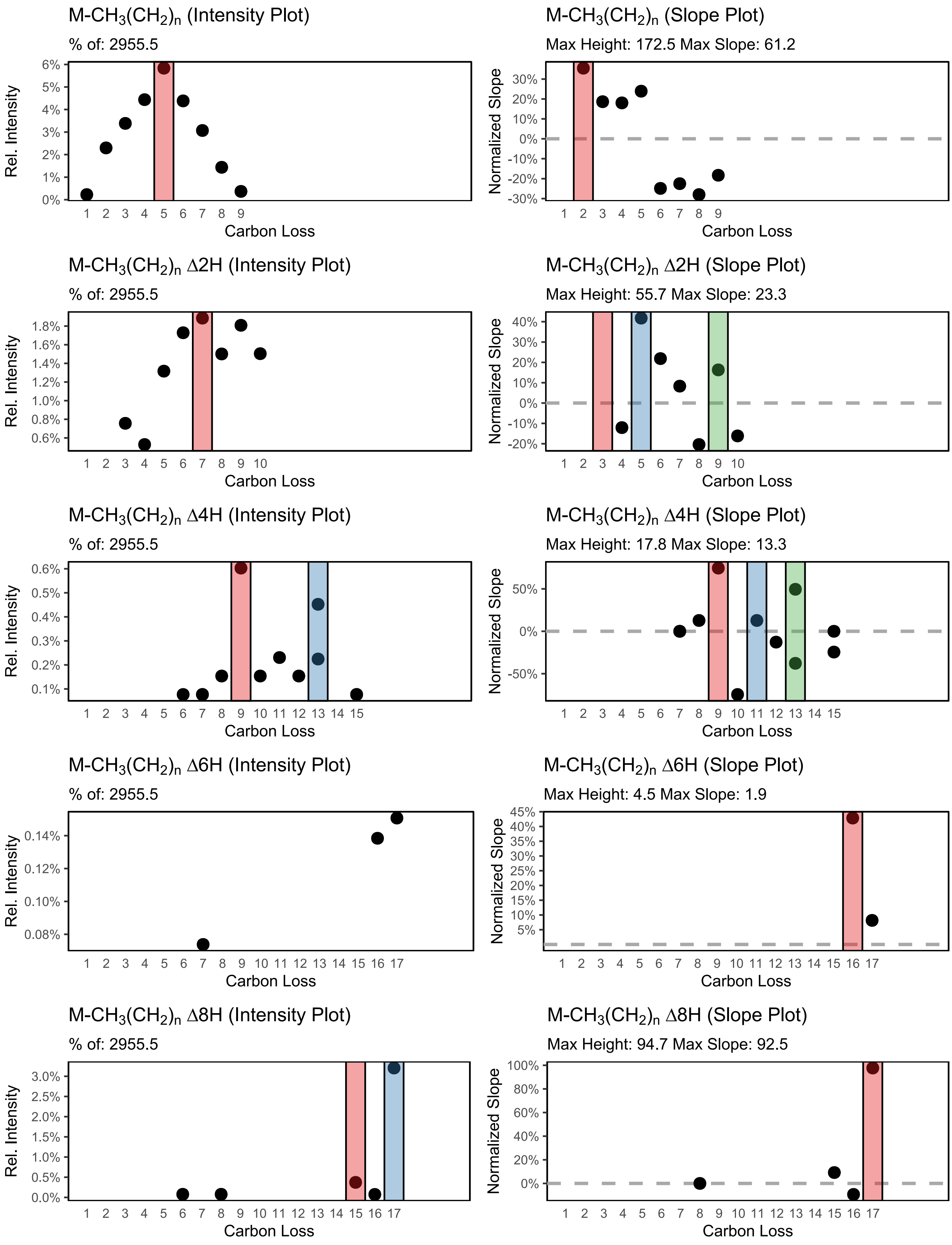

Figure S61: 20 eV CID of 2Ar radical cation with extracted fragmentation series starting from the precursor. Colored bars indicate intensity peak picking results from MsRadaR.

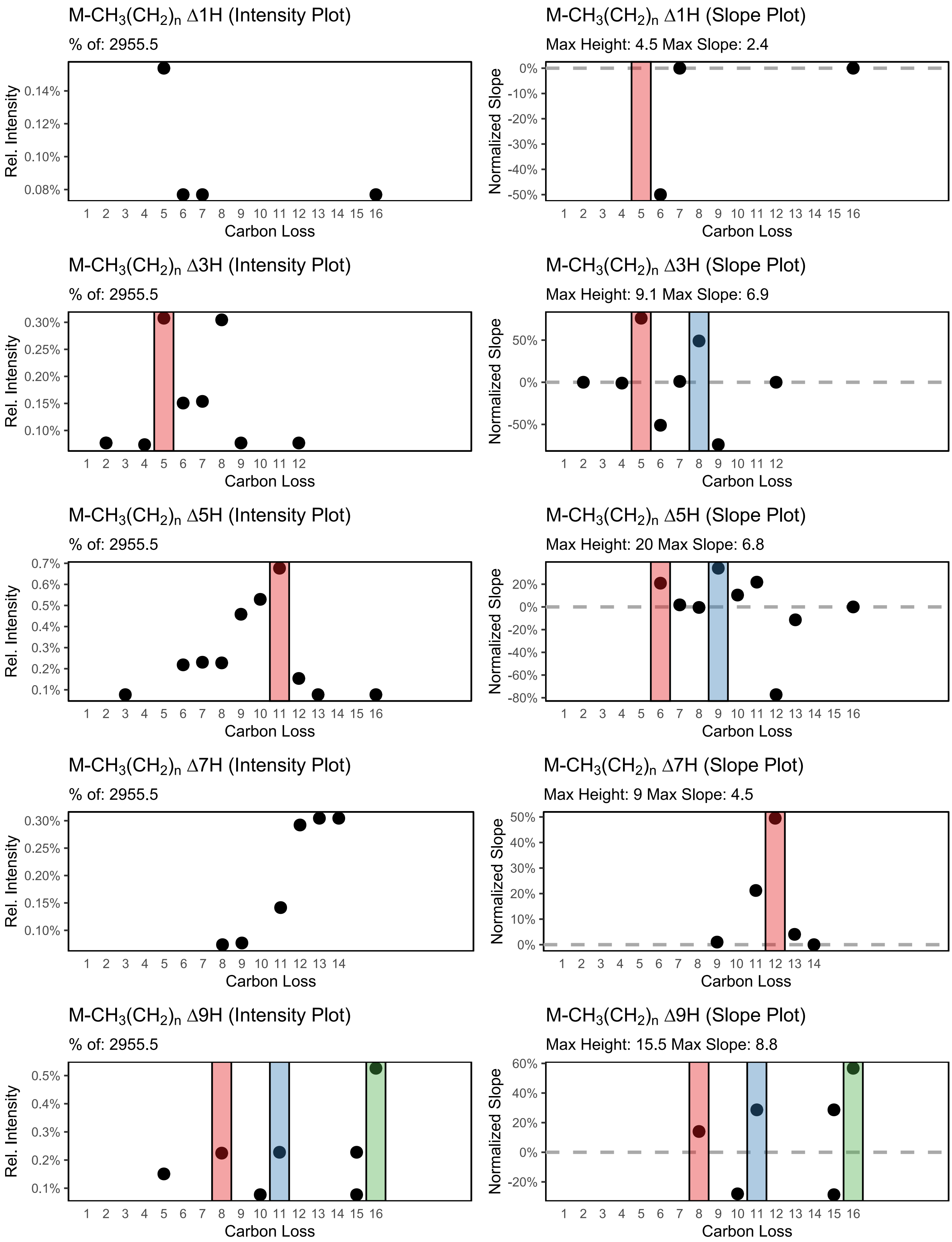

Figure S62: 20 eV CID of 2Ar radical cation with extracted fragmentation series starting from $[M-H_2O]^+$ (*m/z* 358.2449). Colored bars indicate intensity peak picking results from MsRadaR.

A

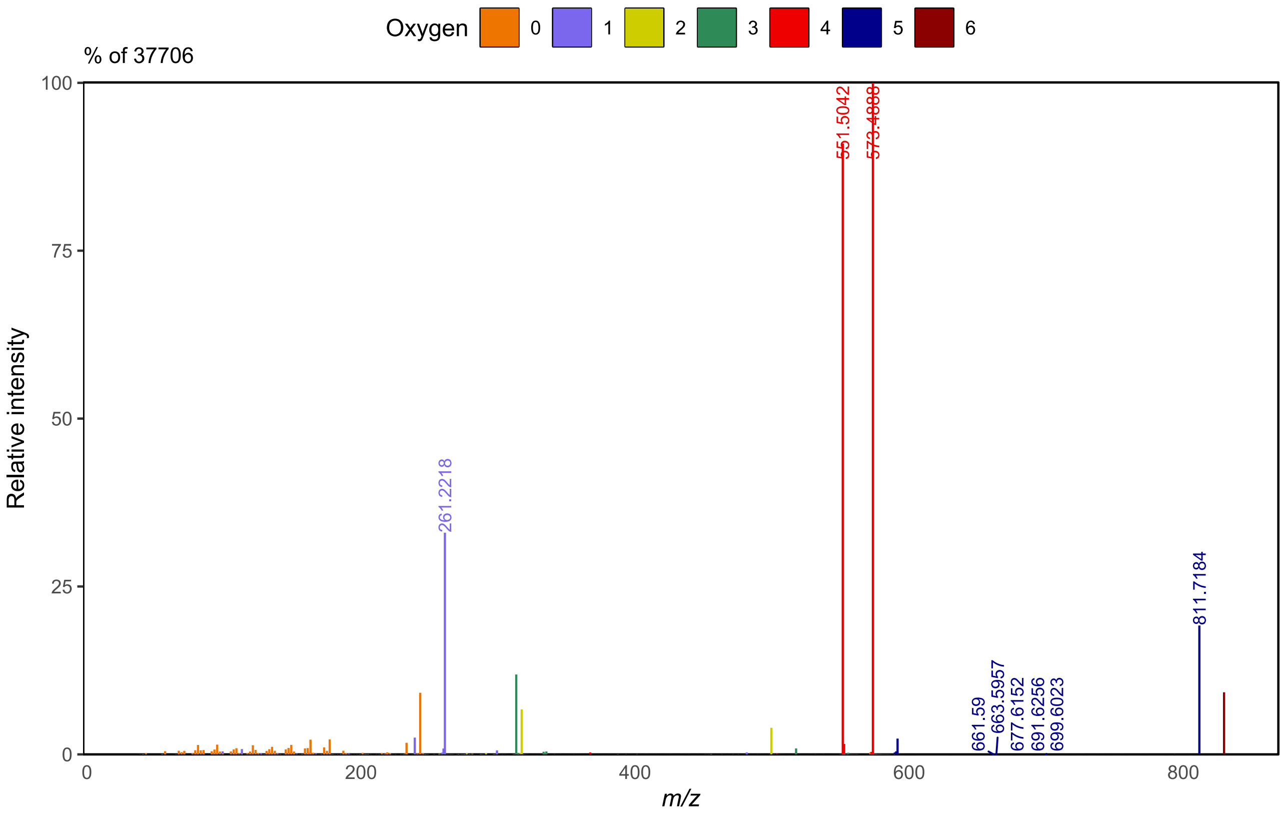

B

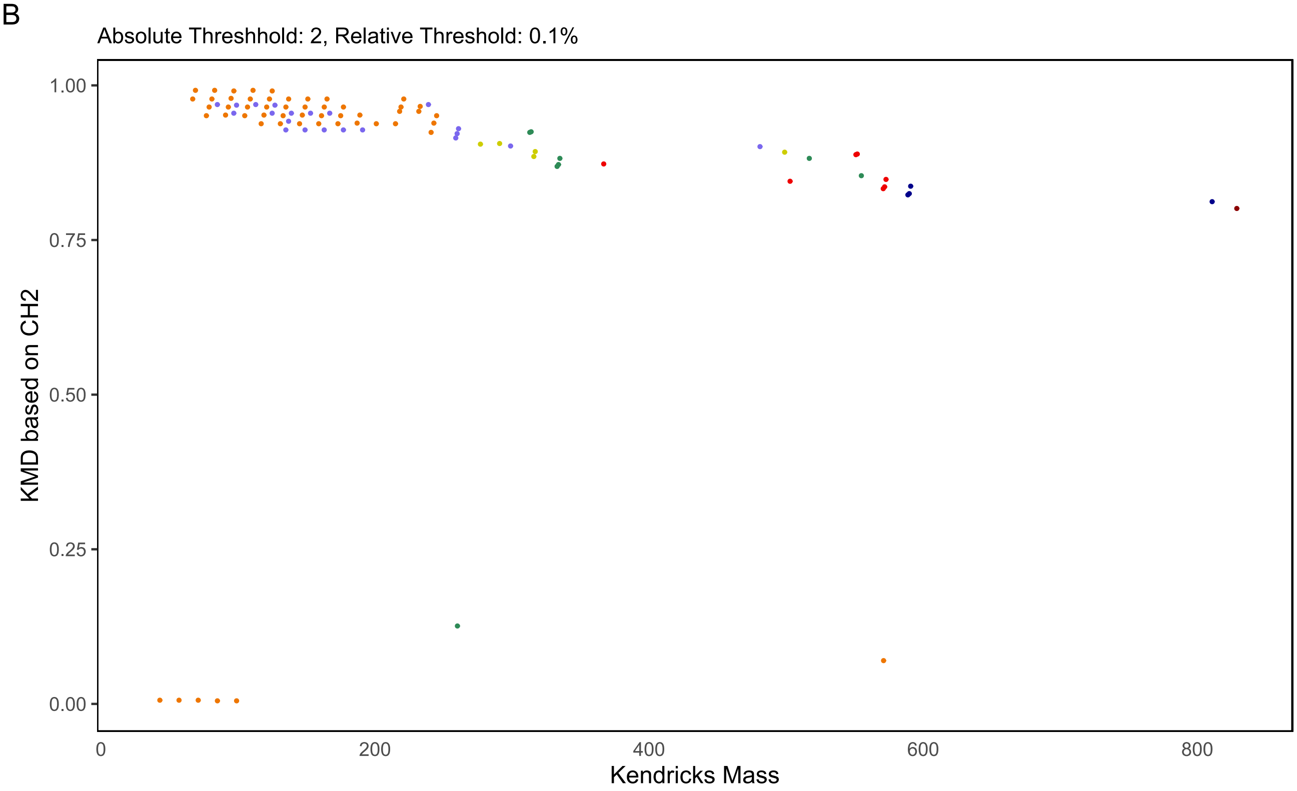

Figure S63: 35 eV CID of PPL $[M-H]^+$ with A) CID spectra and B) Kendricks plot from MsRadaR.

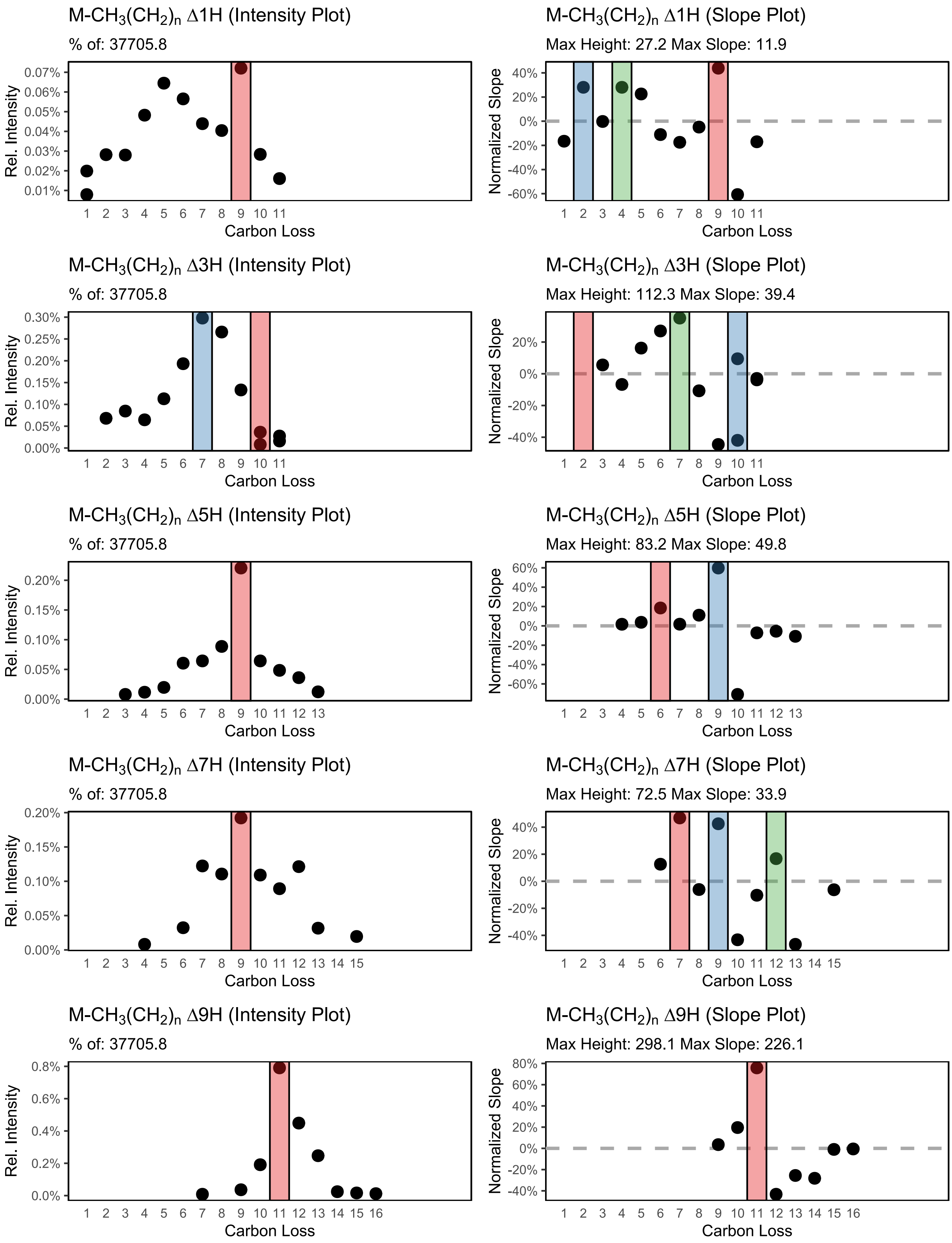

Figure S64: 35 eV CID of PPL $[M-H]^+$ with extracted fragmentation series starting from $[L-H_2O]^+$ (*m/z* 259.2068). Colored bars indicate intensity peak picking results from MsRadaR.

A

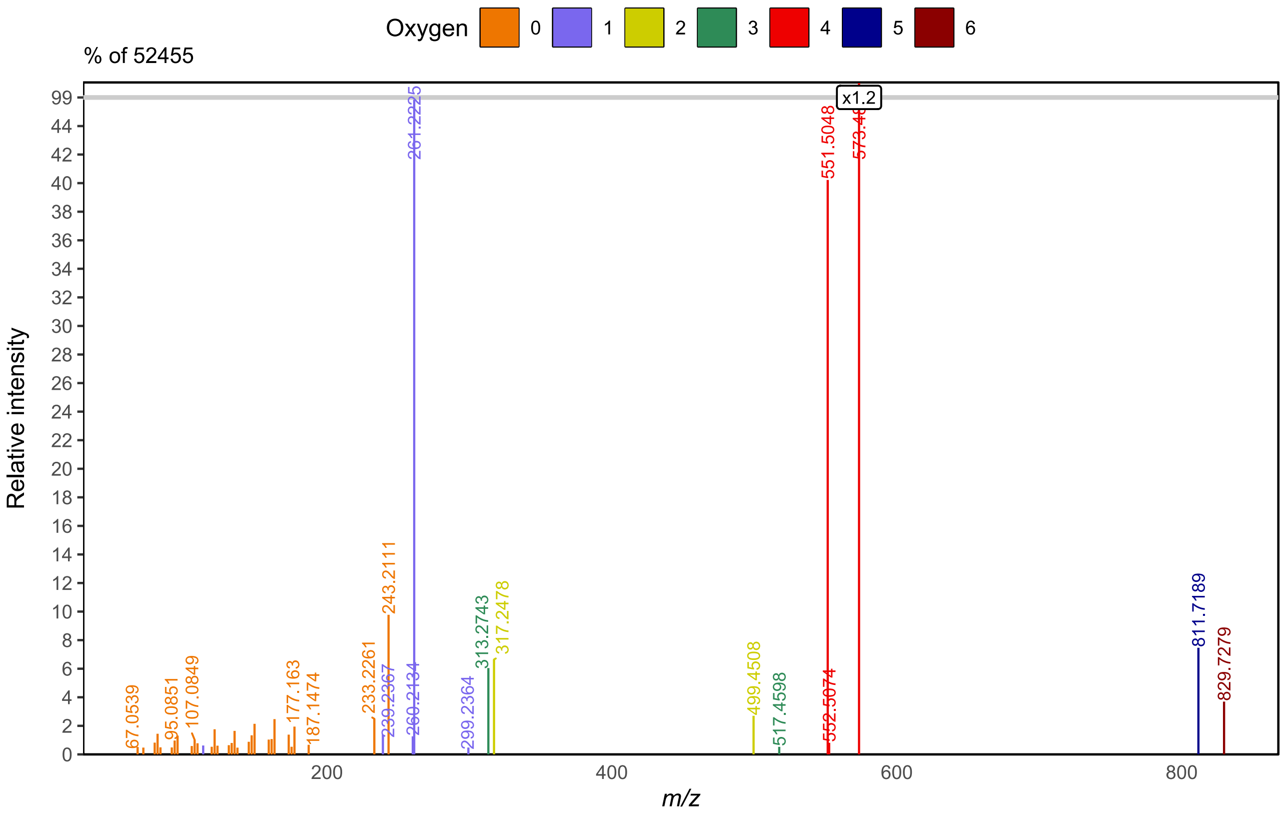

B

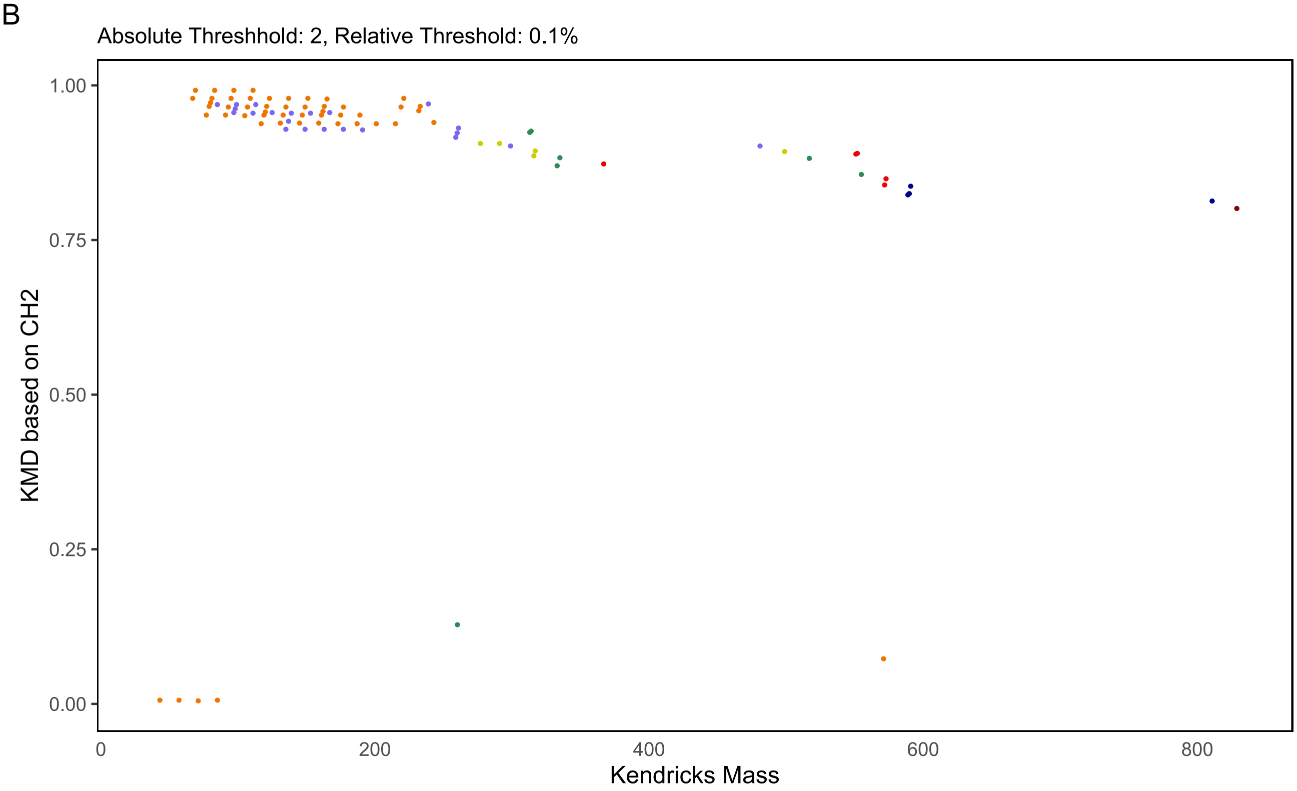

Figure S65: 35 eV CID of PLP [M-H]$^+$ with A) CID spectra and B) Kendricks plot from MsRadaR.

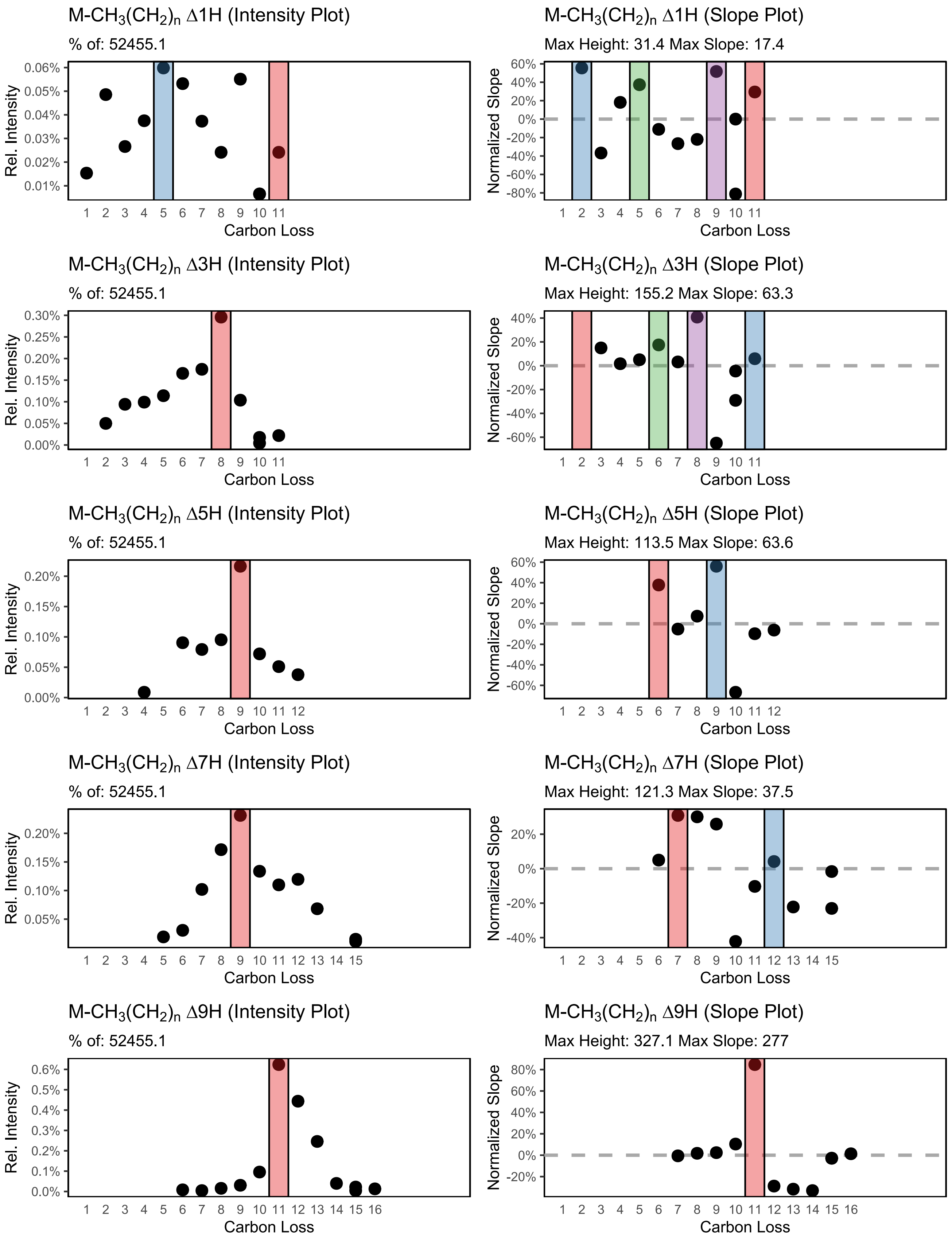

Figure S66: 35 eV CID of PLP [M-H]$^+$ with extracted fragmentation series starting from [L-H_2O]$^+$ (*m/z* 259.2068). Colored bars indicate intensity peak picking results from MsRadaR.

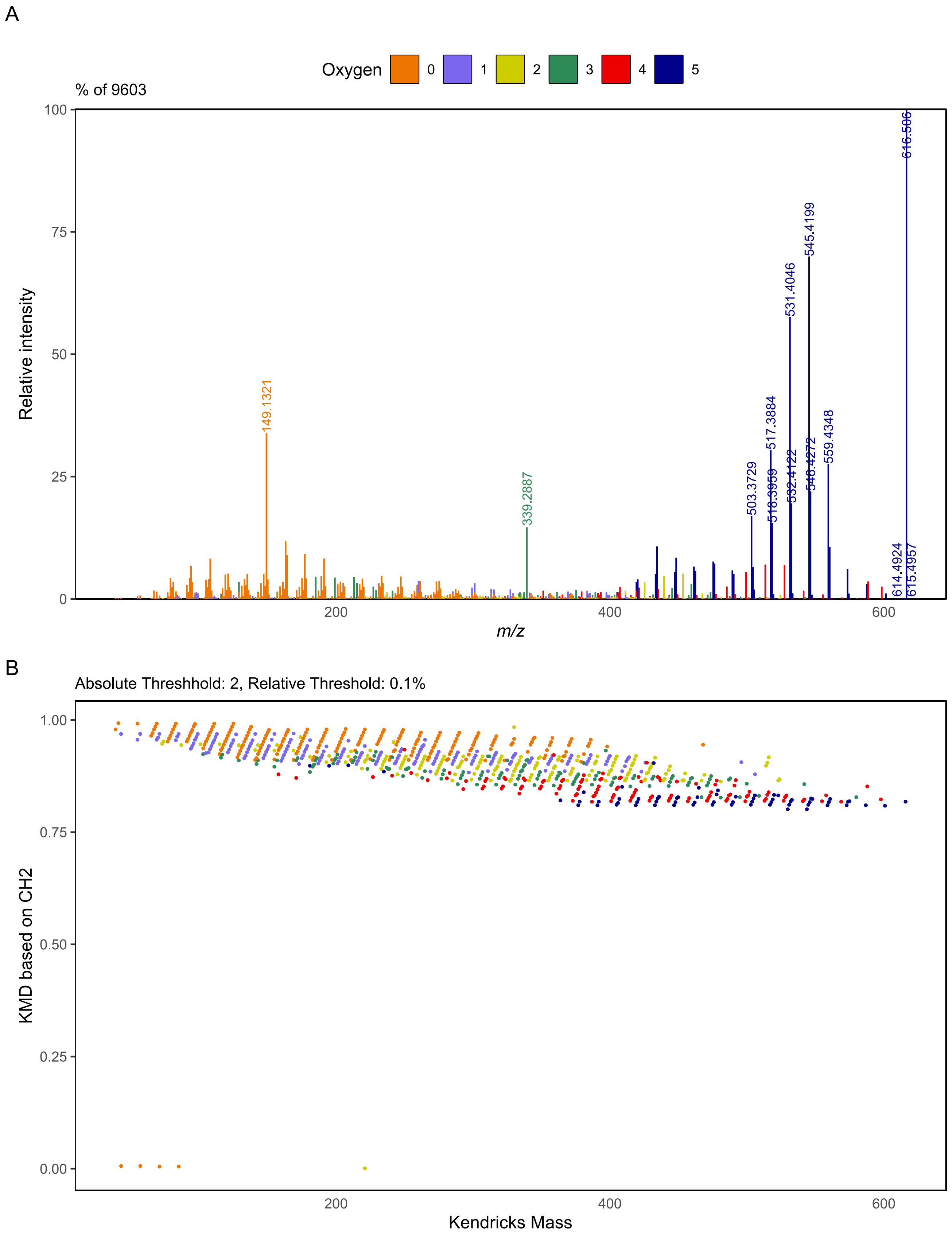

Figure S67: 35 eV CID of 1,2LL radical cation with A) CID spectra and B) Kendricks plot from MsRadaR.

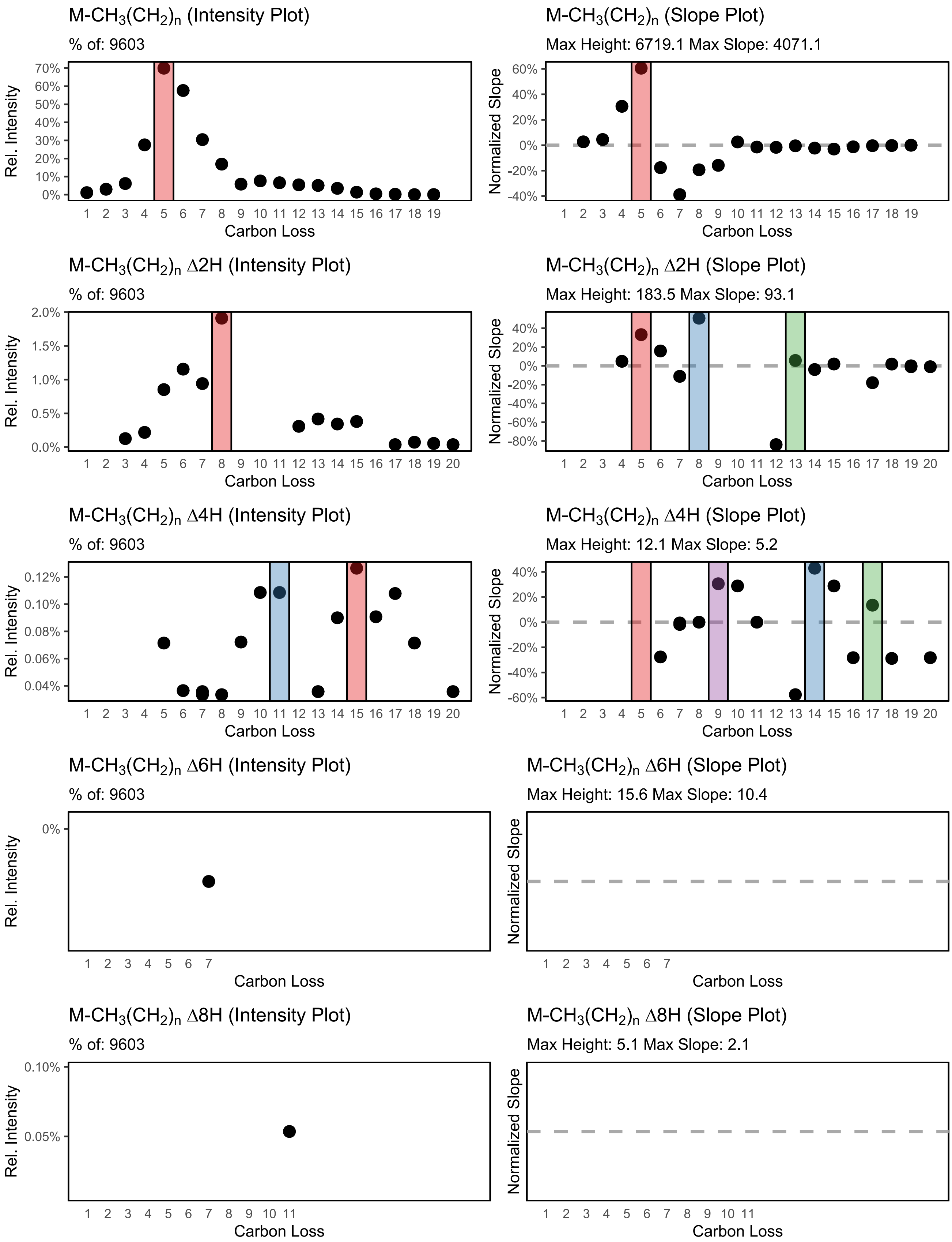

Figure S68: 35 eV CID of 1,2LL radical cation with extracted fragmentation series starting from the precursor. Colored bars indicate intensity peak picking results from MsRadaR.

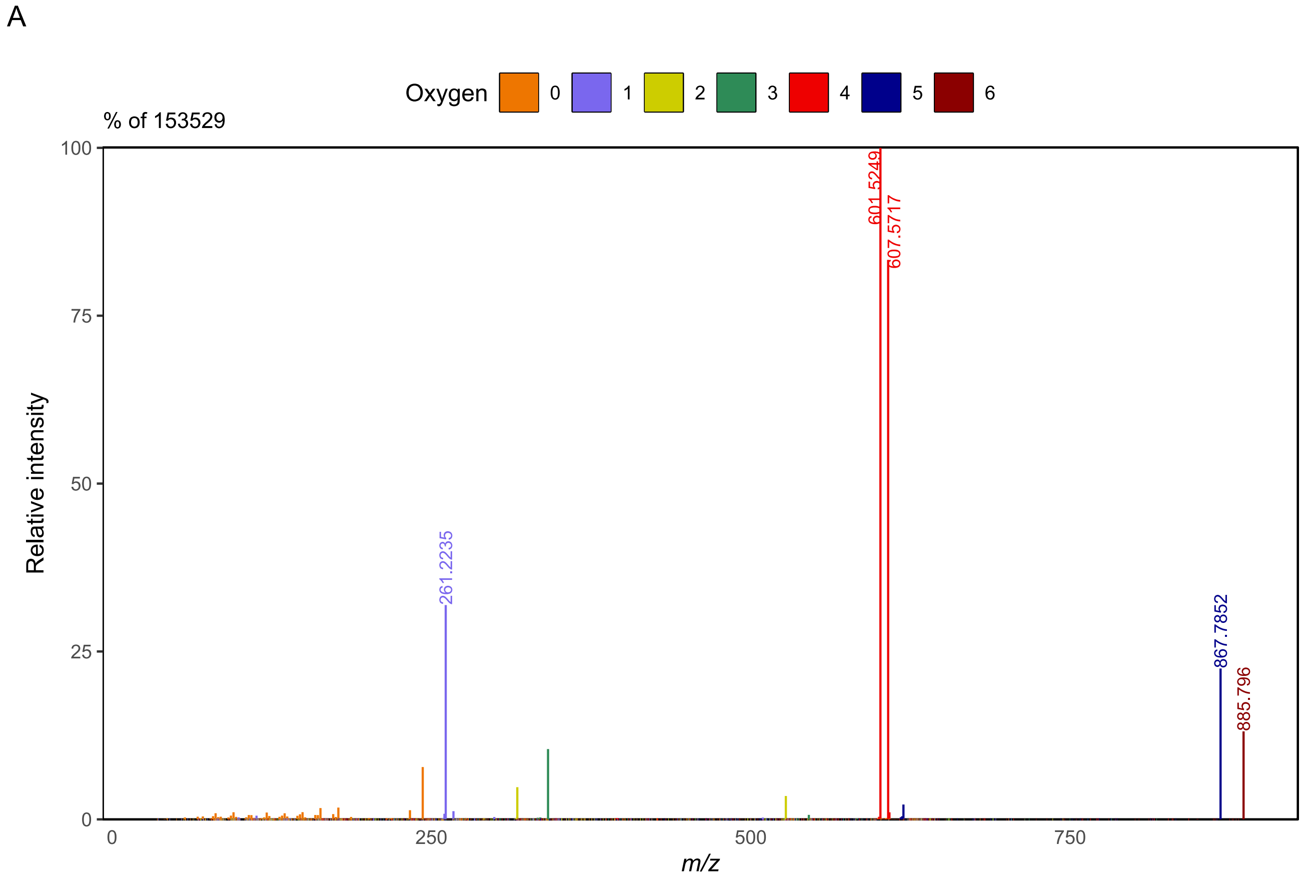

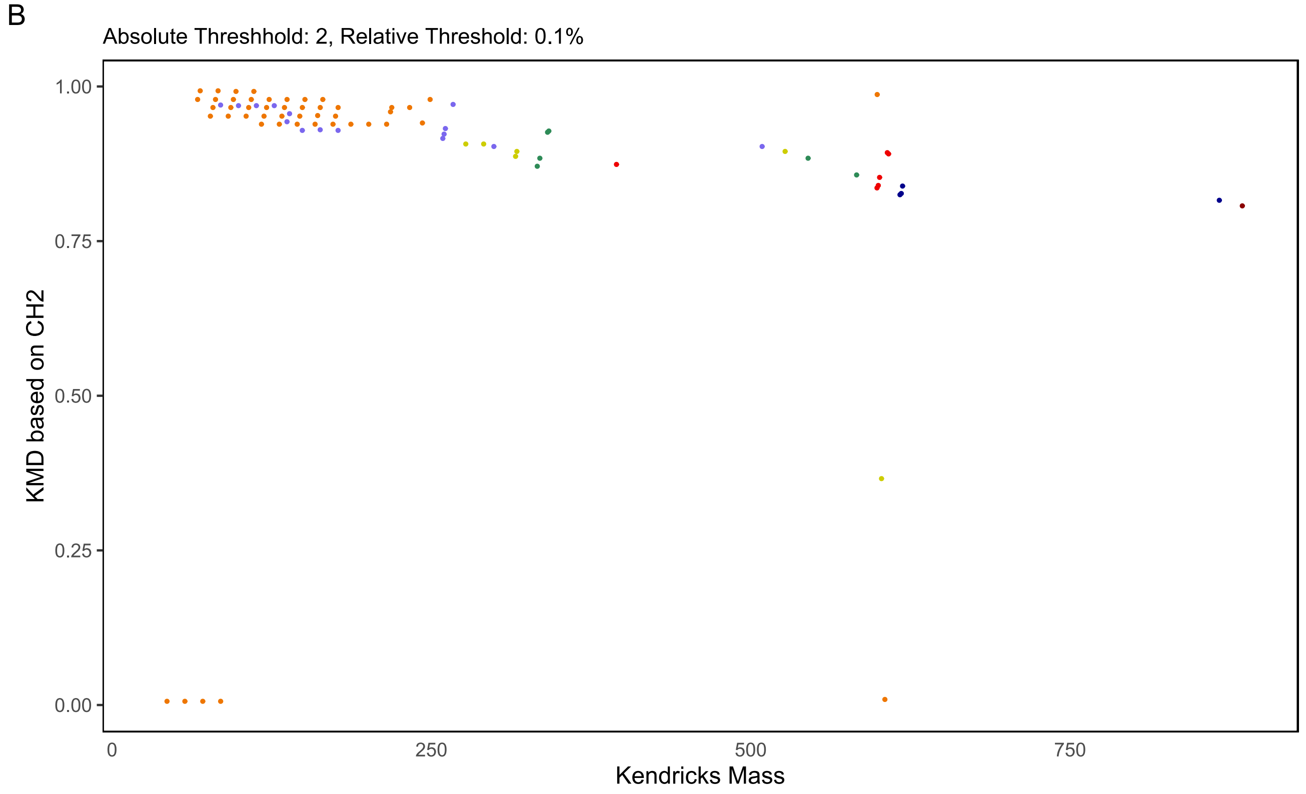

Figure S69: 35 eV CID of SSL $[M-H]^+$ with A) CID spectra and B) Kendricks plot from MsRadaR.

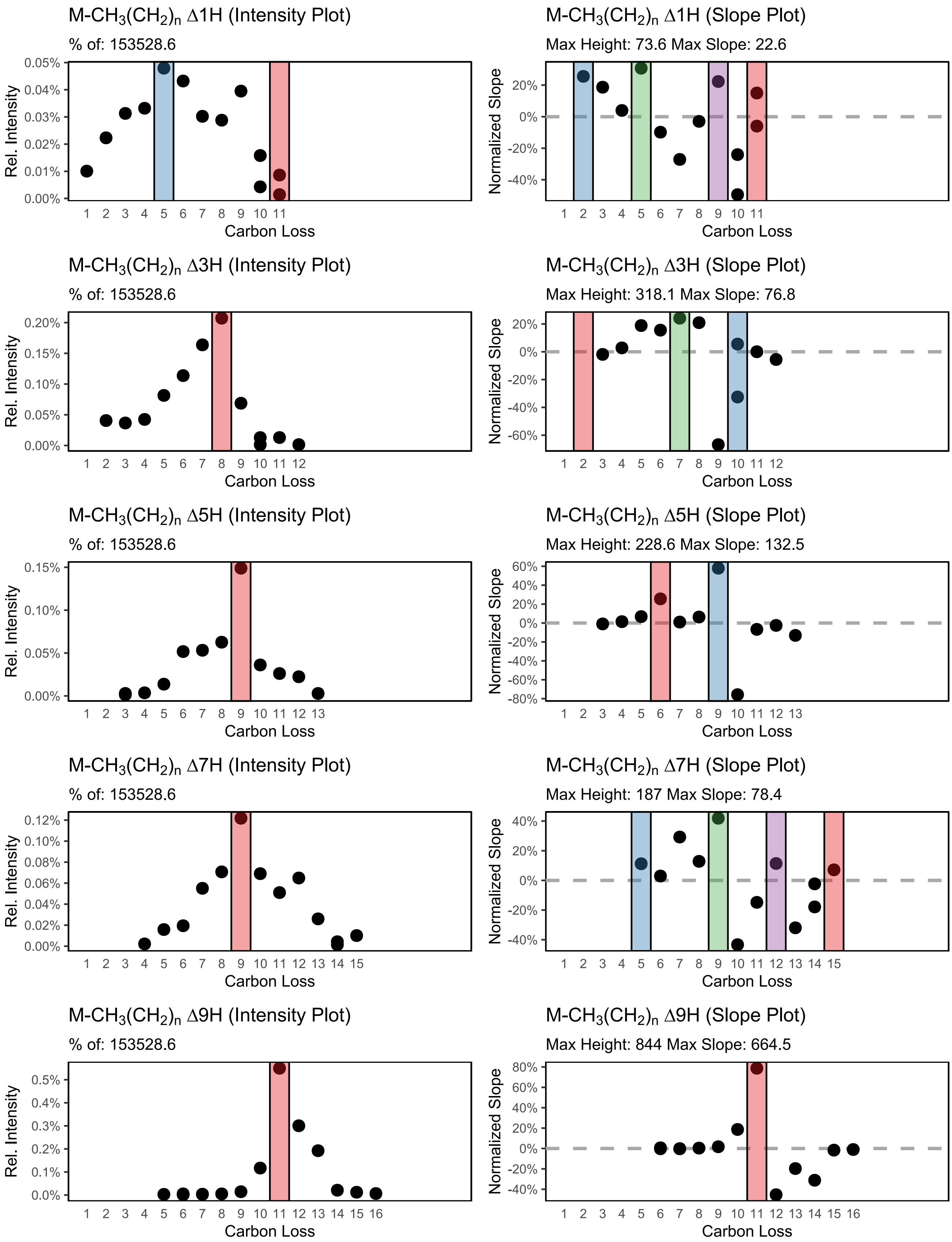

Figure S70: 35 eV CID of SSL $[M-H]^+$ with extracted fragmentation series starting from $[L-H_2O]^+$ (*m/z* 259.2068). Colored bars indicate intensity peak picking results from MsRadaR.

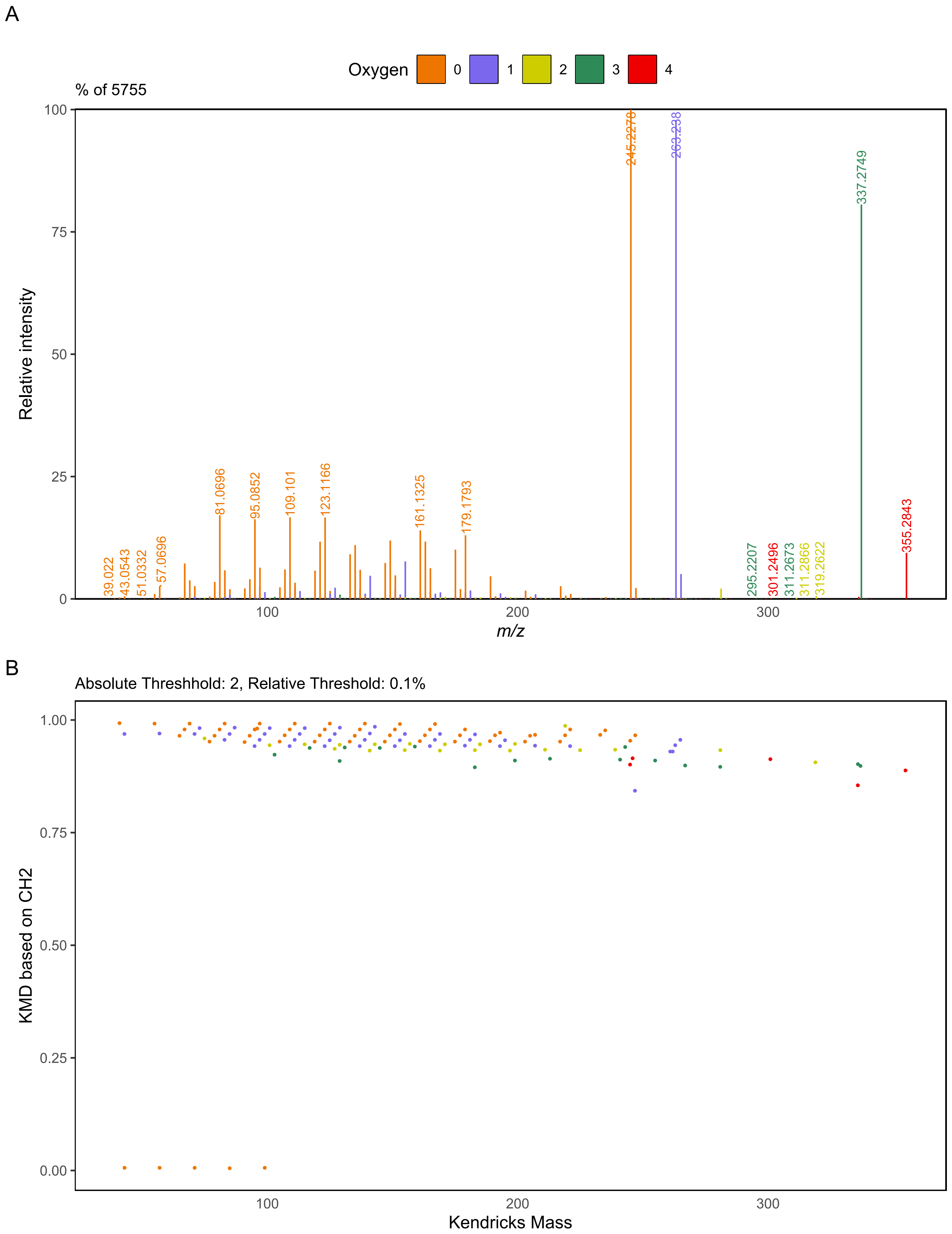

Figure S71: 20 eV CID of 2O [M-H]$^+$ with A) CID spectra and B) Kendricks plot from MsRadaR.

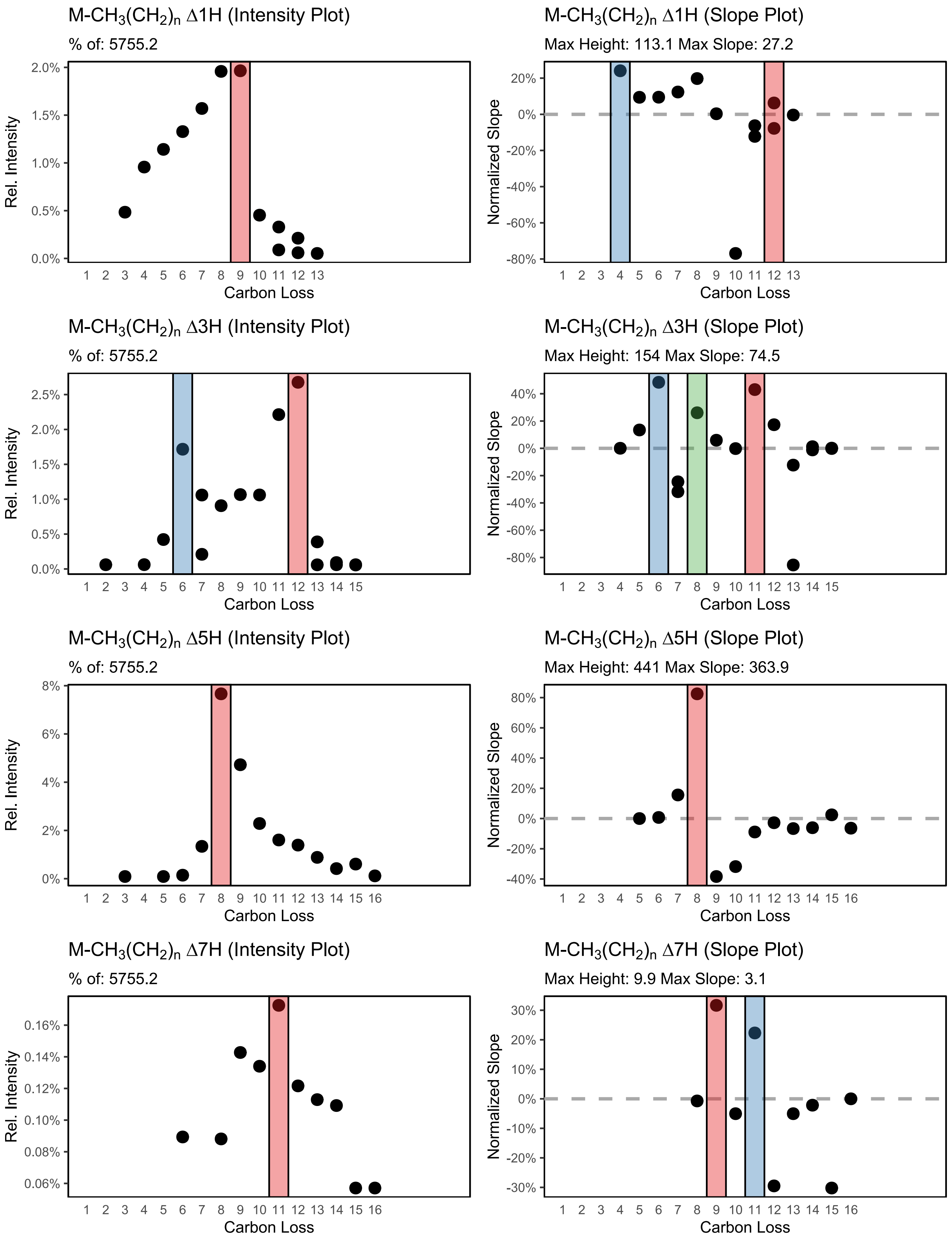

Figure S72: 20 eV CID of 2O $[M-H]^+$ with extracted fragmentation series starting from $[O-H_2O]^+$ (*m/z* 263.2380). Colored bars indicate intensity peak picking results from MsRadaR.

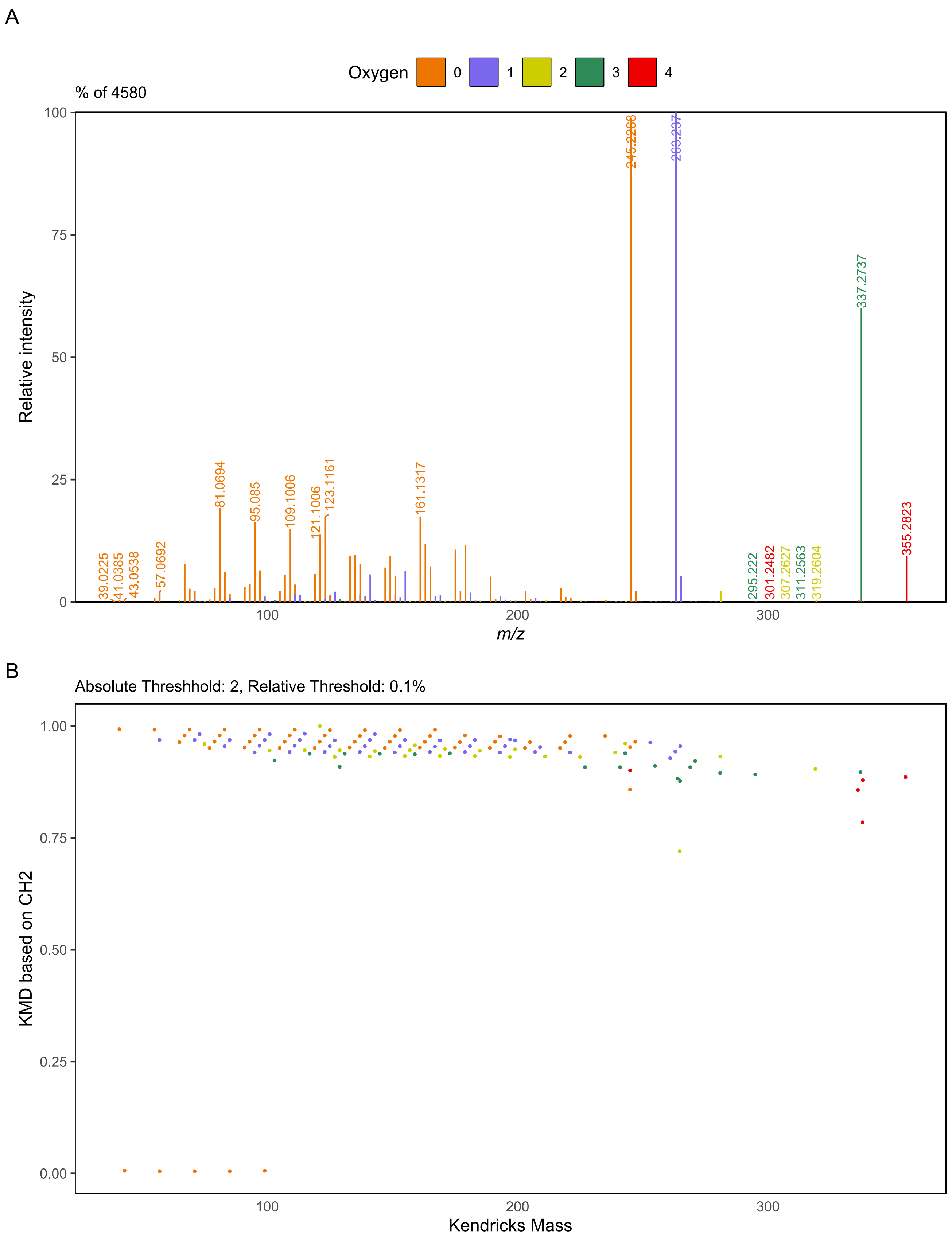

Figure S73: 20 eV CID of 1O $[M-H]^+$ with A) CID spectra and B) Kendricks plot from MsRadaR.

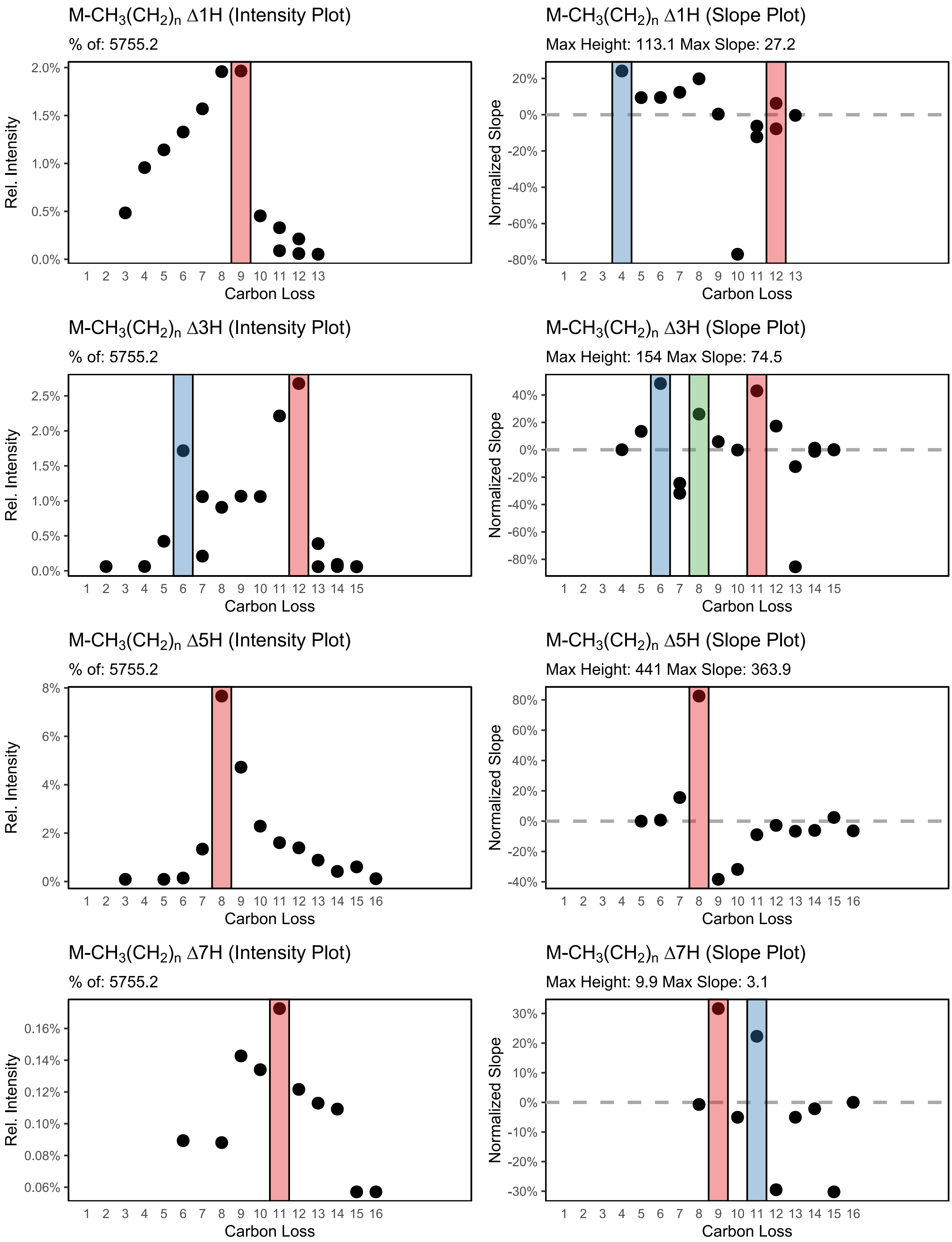

Figure S74: 20 eV CID of 1O $[M-H]^+$ with extracted fragmentation series starting from $[O-H_2O]^+$ (*m/z* 263.2380). Colored bars indicate intensity peak picking results from MsRadaR.

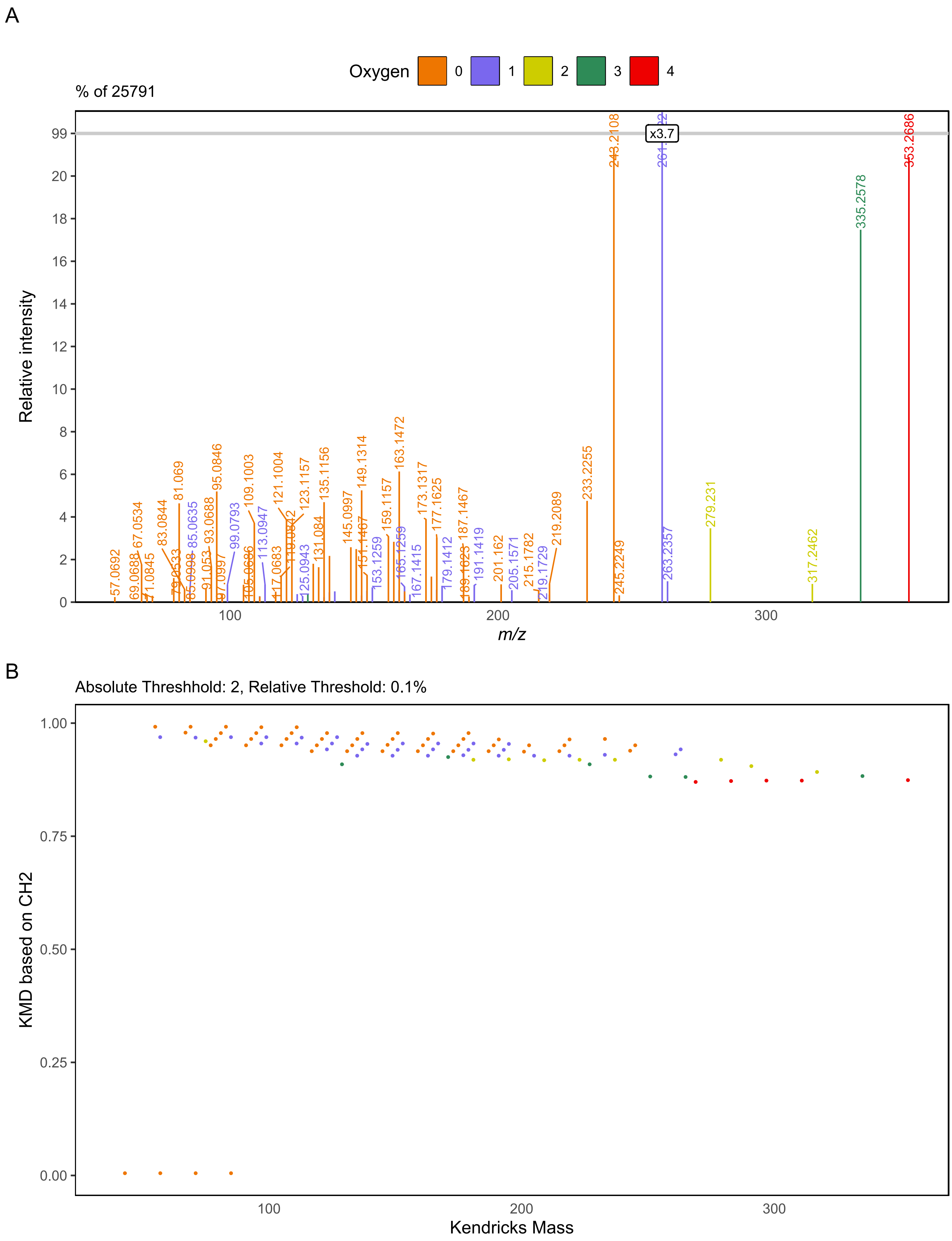

Figure S75: 20 eV CID of 2L [M-H]$^{+}$ with A) CID spectra and B) Kendricks plot from MsRadaR.

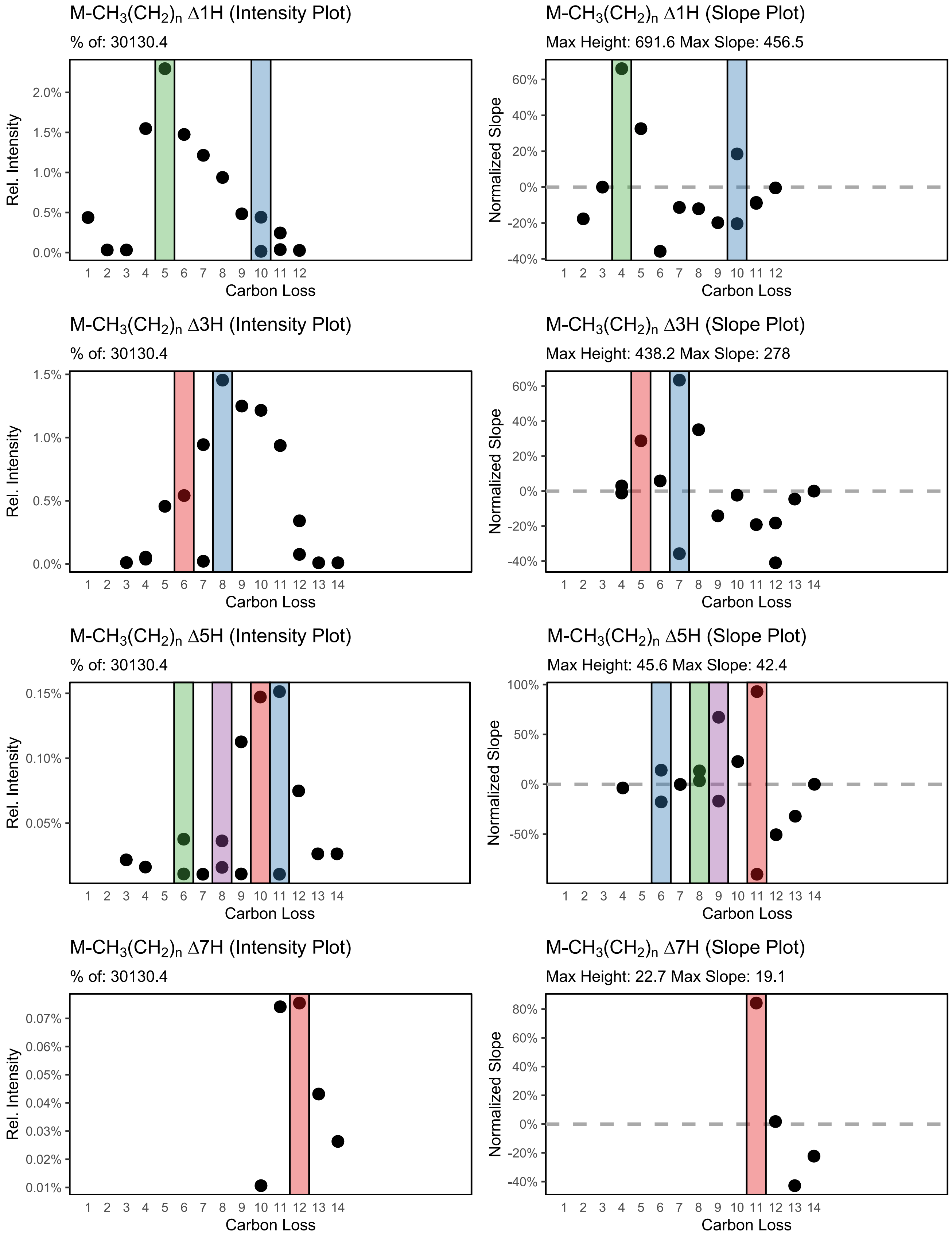

Figure S76: 20 eV CID of 2L $[M\text{-}H]^+$ with extracted fragmentation series starting from *m/z* 233.2247. Colored bars indicate intensity peak picking results from MsRadaR.

A

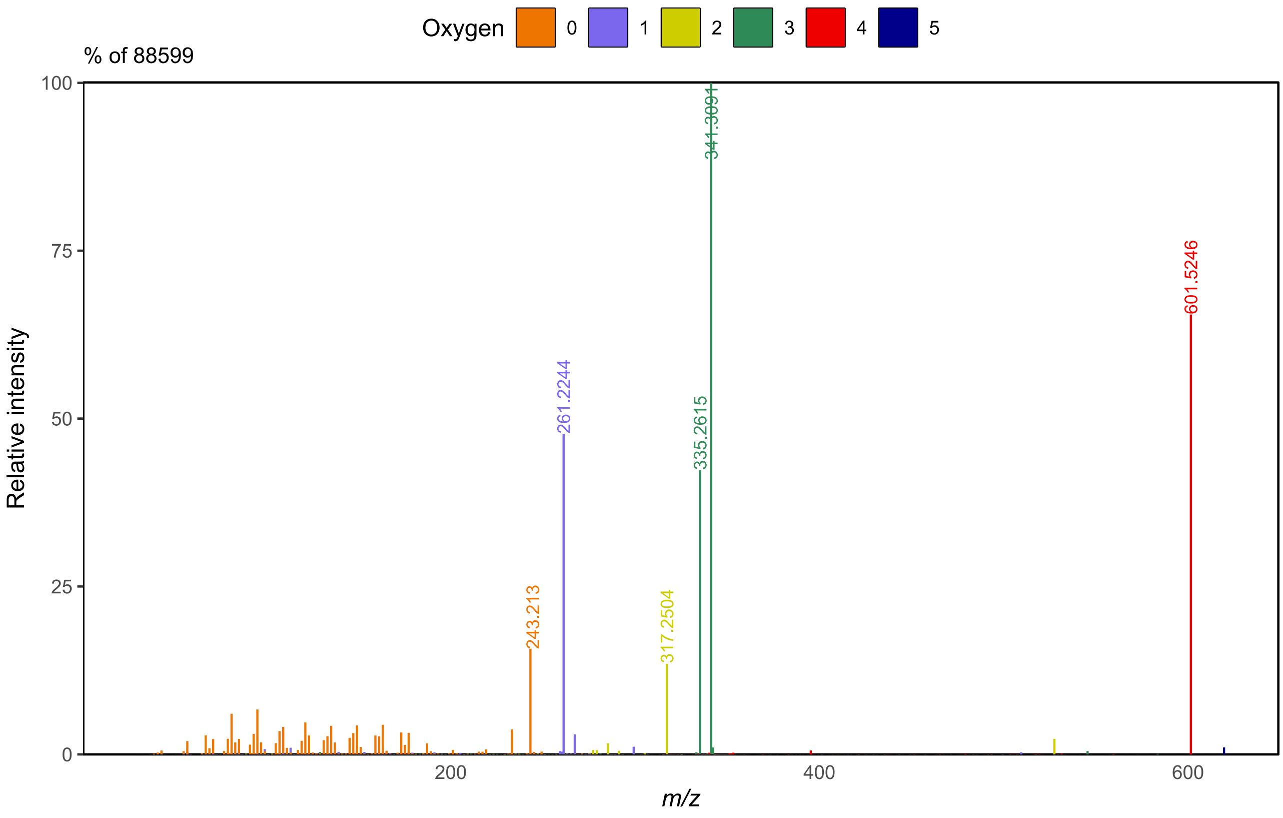

B

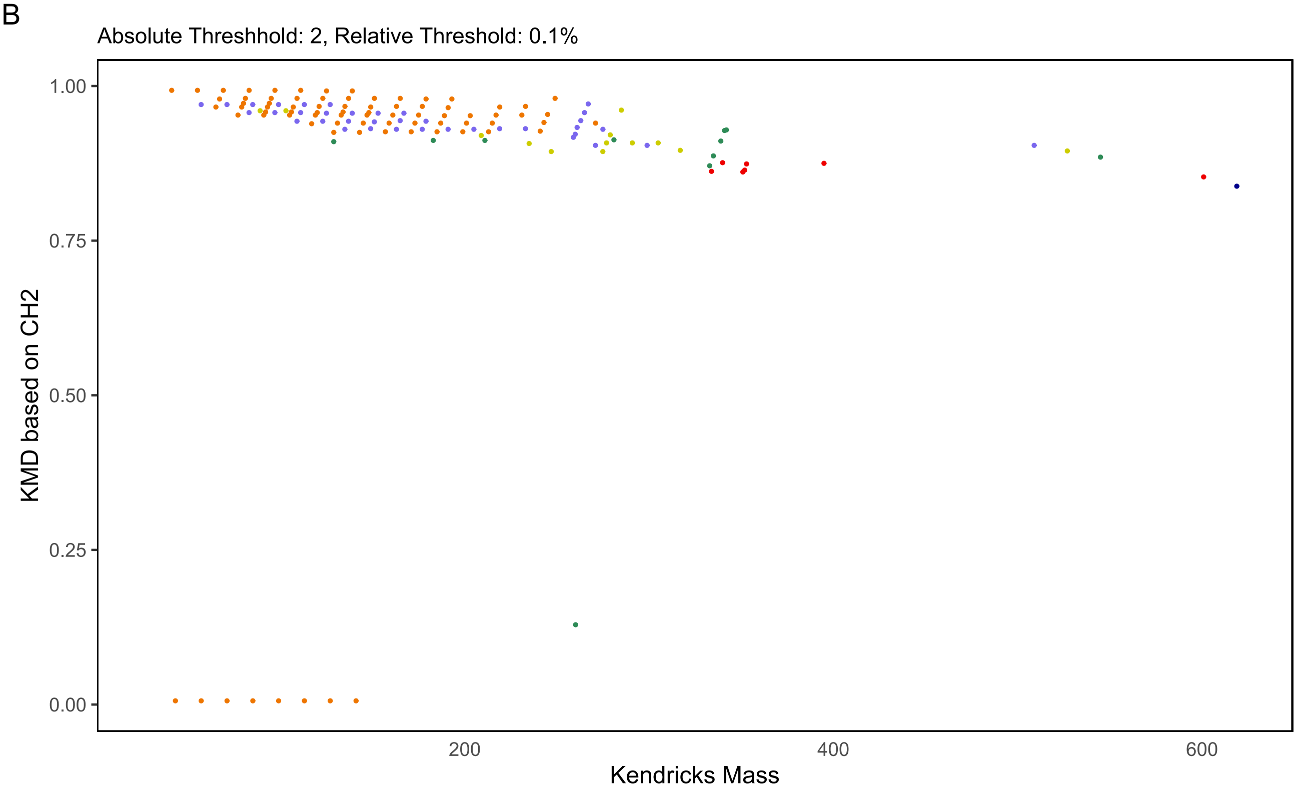

Figure S77: 35 eV CID of 1,3SL [M-H]$^+$ with A) CID spectra and B) Kendricks plot from MsRadaR.

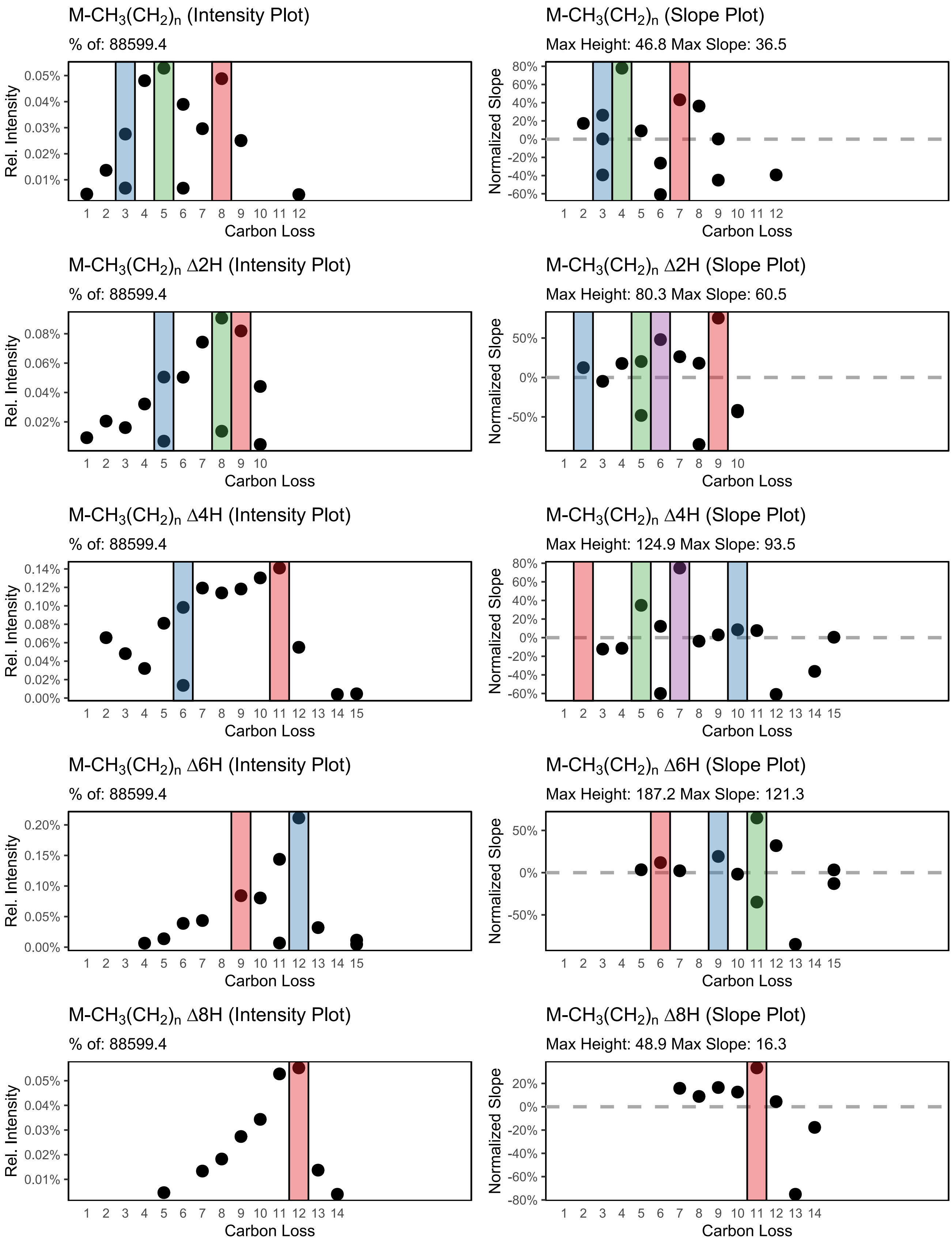

Figure S78: 35 eV CID of 1,3SL [M-H]$^+$ with extracted fragmentation series starting from *m/z* 243.213. Colored bars indicate intensity peak picking results from MsRadaR.

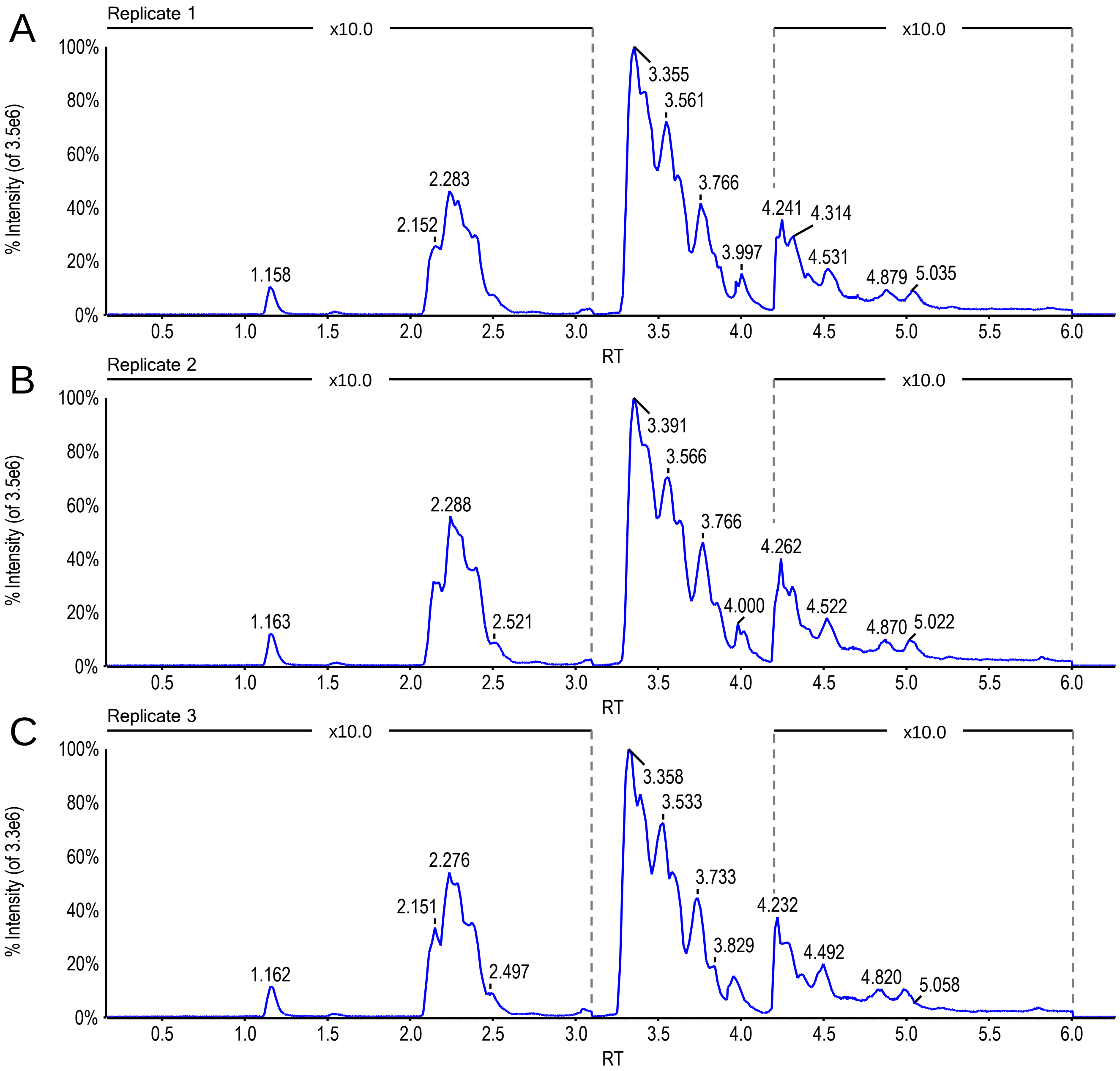

Figure S79: Linseed oil chromatograms using SFC-APPI-EDP-CID with data-dependent acquisition with A) replicate 1, B) replicate 2 and C) replicate 3.

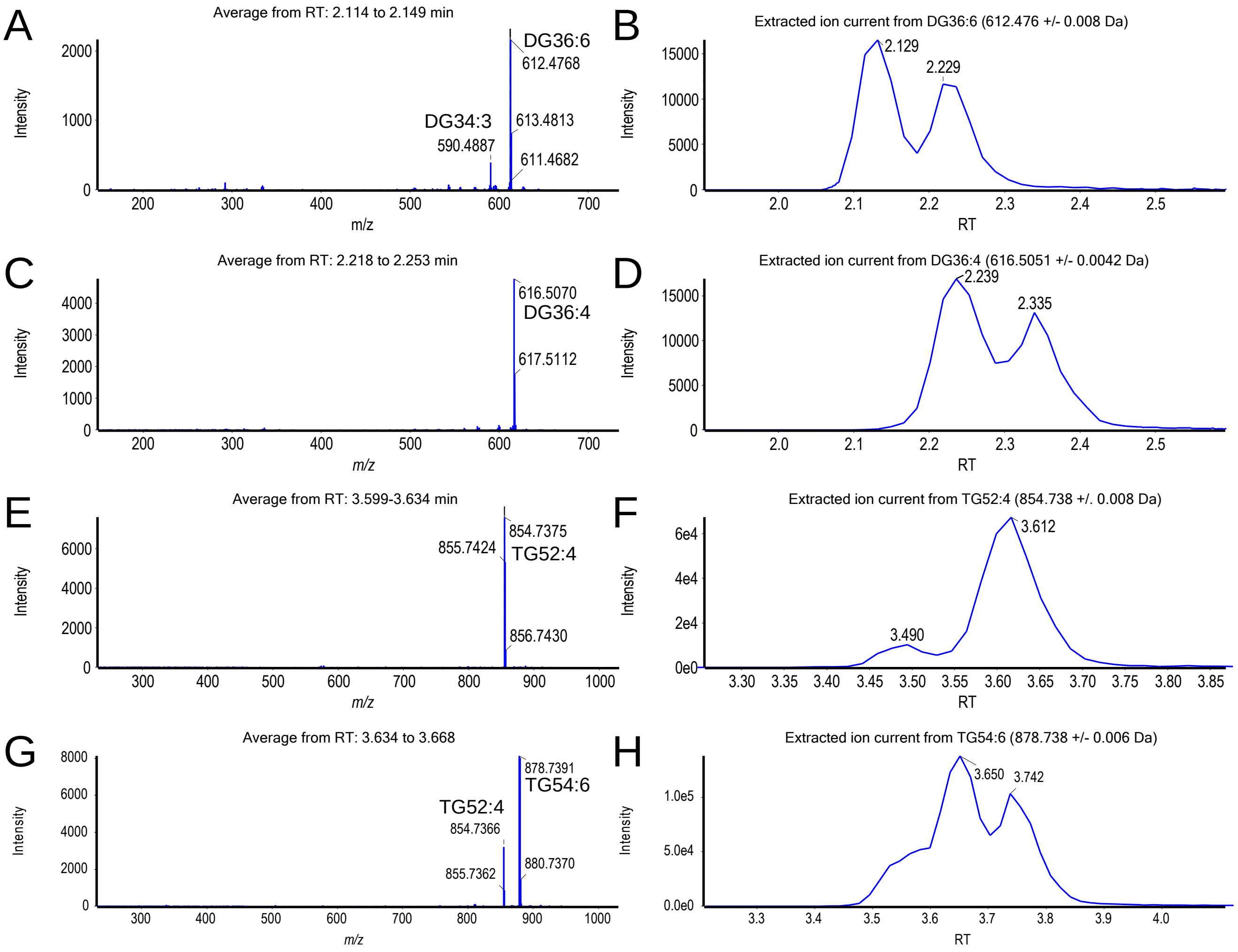

Figure S80: Selected isomeric example MS1 spectra with dynamic background subtraction and corresponding extracted ion-currents with A) MS1 spectra of DG36:6 and B) XIC of DG36:6, C) MS1 spectra of DG36:4 and D) XIC of DG36:6 and E) MS1 spectra of TG52:4 and F) XIC of TG52:4 and G) MS1 spectra of TG54:6 with H) XIC of TG54:6.
